# AMPK phosphorylates WIP1 to promote DNA repair and radioresistance in cancer cells

**DOI:** 10.1038/s41419-025-08141-7

**Published:** 2025-11-28

**Authors:** Manman Lu, Xiaochuan Dong, Chunrui Wu, Guisong Wang, Haiyang Wang, Yingli Pan, Yali Qin, Yushuai Song, Hongming Pan, Shenzhi Liu, Kun Zhang, Xuewu Zhang, Jing Qu, Zhenhua Yang

**Affiliations:** 1 School of Basic Medicine, Tongji Medical College, Huazhong University of Science and Technology, Wuhan, China; 2 Department of Pathology, Union Hospital, Tongji Medical College, Huazhong University of Science and Technology, Wuhan, China; 3 Hubei Key Laboratory of Applied Mathematics, Faculty of Mathematics and Statistics, Hubei University, Wuhan, China; 4 School of Basic Medicine and Forensic Medicine, Henan University of Science & Technology, Luoyang, China; 5 Hepatic Surgery Center, Tongji Hospital, Tongji Medical College, Huazhong University of Science and Technology, Wuhan, China; 6 Hubei Key Laboratory of Hepato-Pancreato-Biliary Diseases, Wuhan, China; 7 Hubei Key Laboratory of Drug Target Research and Pharmacodynamic Evaluation, Wuhan, China; 8 Key Laboratory of Anesthesiology and Resuscitation (Huazhong University of Science and Technology), Ministry of Education, Wuhan, China

**Keywords:** Molecular biology, DNA damage and repair

## Abstract

Cell metabolism has a profound impact on maintaining genomic stability. AMP-activated protein kinase (AMPK) is a crucial regulator of cell metabolism and the maintenance of genomic stability. There is increasing evidence that AMPK plays a crucial role in the efficient response to DNA damage (DDR). However, the underlying mechanism is still unclear. Here, we show that glucose deprivation rapidly reduces γH2AX levels, a hallmark of DNA damage. We then found that WIP1, rather than PP2A or PP4C, is the primary phosphatase responsible for dephosphorylating γH2AX under both normal and damaged conditions. Molecular studies have revealed that AMPK directly binds and phosphorylates WIP1 at Thr25 (T25). This action enhances protein stability and the binding ability of WIP1 with γH2AX, likely promoting the enzyme activity of WIP1 and subsequently reducing the level of γH2AX. These processes facilitate DNA damage repair and contribute to the radioresistance of tumor cells. The findings provide experimental evidence of a novel link between metabolic stress and DDR, suggesting that AMPK may promote the resistance of tumor cells to radiation therapy by phosphorylating WIP1.

## Introduction

Endogenous metabolites (e.g., ROS) and exogenous sources (e.g., ionizing radiation) constantly induce DNA damage. Upon detection of these damage signals, the histone H2A variant H2AX is rapidly phosphorylated at Ser139 by PI(3)K (phosphatidyl-inositol-3-OH kinase)-like kinases, ATM (ataxia telangiectasia mutated), ATR (ATM- and Rad3-related) and DNA-PK (DNA-dependent protein kinase) to form gamma H2AX (γH2AX), which is essential for efficient recognition and repair of DNA double-strand breaks (DSBs) [1, 2]. It has been reported that the pleiotropic phenotypes of H2AX-null mice are associated with repair defects, suggesting that γH2AX plays a vital role in maintaining genomic stability [3]. Upon activation, γH2AX rapidly serves as a platform by recruiting numerous factors, such as MDC1, 53BP1, and the downstream MRE11-RAD50-NBS1 (MRN) complex, to DNA break sites, facilitating precise DNA repair [4]. Immunofluorescence staining (IF) shows that this highly amplified process generates nuclear foci that are widely recognized as markers for DSBs [5].

The DNA damage signal triggers cell cycle arrest to ensure efficient repair [6]. After successful repair of DSBs, damage/repair signals are terminated by efficiently deleting γH2AX to maintain H2AX/γH2AX homeostasis. It has been reported that γH2AX is mainly dephosphorylated by PP2A and PP4, of which PP4C is responsible for the removal of γH2AX induced by relatively low levels of DNA damage (basal level), and PP2A might be required for higher levels of damage (e.g., CPT) in mammals [7, 8]. In addition to PP4C and PP2A, protein phosphatase 1 alpha (PP1α) [9] and wild-type p53-induced phosphatase 1 (WIP1) [10] have also been shown to dephosphorylate γH2AX. However, regulating phosphatase enzyme activity and its functional effects on γH2AX remains a complex and intriguing question, with many aspects still unclear.

AMP-activated protein kinase (AMPK) is a heterotrimeric protein kinase consisting of a catalytic (α) subunit and two regulatory (β, γ) subunits. It is activated by liver kinase B1 (LKB1) or calcium-calmodulin-dependent protein kinase β (CAMKKβ) mediated phosphorylation under conditions of a high AMP-to-ATP ratio (low cellular energy) or increased intracellular calcium, respectively [11]. In addition to redirecting metabolism toward increased catabolism, activated AMPK is involved in numerous biological processes, including autophagy, mitosis, and transcription, by phosphorylating essential proteins [12–14]. Recently, several studies have shown that AMPK plays a role in DNA DSB repair. For example, deleting AMPKα leads to the accumulation of ROS, which can induce DNA breakage in either mouse embryonic fibroblasts (MEFs) or mouse leukemia cells [14–16]. These studies suggest that AMPK maintains genomic stability by limiting the production of ROS. However, research on metformin and AICAR, two well-known activators of AMPK, has shown that they can increase ROS levels while having little effect on cell proliferation in some cell types [17–19]. Therefore, the mechanism by which AMPK participates in and regulates DNA damage repair remains unclear.

Starting with the observation that activating AMPK through glucose deprivation was inversely related to γH2AX levels, we explored the crucial role of AMPK in DNA damage repair by directly phosphorylating WIP1 at Thr25. Phosphorylation of WIP1 at Thr25 enhanced its stability and its ability to bind with γH2AX, both of which likely help dephosphorylate γH2AX. This process is linked to effective DNA damage repair and contributes to the radioresistance of cancer cells. Our findings reveal a new connection between metabolic stress and DDR, which may also shed light on the role of AMPK in tumor development.

## Materials and methods

### Animals

Experiments on BALB/c-Nude mice (Strain NO. D000521) were performed in the animal facility of Huazhong University of Science and Technology (Wuhan, China) with the approval of the Institutional Animal Care and Use Committee (IACUC) of Huazhong University of Science and Technology (IACUC number: 4017). Mice were purchased from GemPharmatech (Nanjing, China) and maintained under specific pathogen-free conditions in individually ventilated cages (IVC) with a 12-h light-dark cycle and free access to food and water.

### Clinical samples

Breast cancer tissue samples were obtained from 13 patients (aged 32–84) who had undergone breast surgery at Union Hospital of Huazhong University of Science and Technology. None of these patients received immunotherapy and had no other pre-existing diseases before surgery. Informed consent was obtained from all participants. The Medical Ethics Committee of Huazhong University of Science and Technology approved this study (Approval number: UHCT-IEC-SOP-016-03-01).

### Cell culture and antibodies

293T, HeLa, and MCF7 cells were maintained in DMEM medium (Invitrogen) containing 10% fetal bovine serum (FBS), respectively. THP1 and MOLM13 leukemia cells were cultured in RPMI 1640 (Invitrogen) medium with 10% FBS. The Stem Cell Bank of the Chinese Academy of Sciences provided all the cell lines. Mouse embryonic fibroblast (MEF) cells expressing wild-type AMPK (WT) and cells with double knock out of AMPK α1/α2 (AMPKa1/a2, KO) were kindly provided by Dr. Jia Li (Shanghai Institute of Materia Medica, Chinese Academy of Sciences) [20]. The antibodies against WIP1 (Cat #: 11901), ACC (Cat #: 3676), p-ACC (Cat #: 11818), H2AX (Cat #: 7631), γH2AX (Cat #: 9718), AMPK (Cat #:9751), p-AMPK (Cat #: 2535), p-P53 (Ser15) (Cat #: 9284) Actin (Cat #:3700) and Flag tag (Cat #:14793) that used for western blotting or IF assays were purchased from Cell Signaling Technology (CST). M2 magnetic beads were obtained from Merck (Cat #: M88233).

### Lentiviruses, gene knockdown, and overexpressing plasmids

pLKO.1-based lentiviral plasmids were constructed, and viral particles were produced in 293T cells by following the recommended protocols (Addgene) as we described [21–23]. Cells were infected with viruses in the presence of 8 µg/ml of polybrene (Sigma). One day after infection, puromycin was added (final concentration is 2 µg/ml) and maintained for 2–3 days to select the infected cells. As indicated in the figure legends, puromycin-resistant cells were used for various assays. The overexpression plasmids were constructed by loading the individual fragments with different tags onto the pcDNA3.1 vector.

### Quantitative PCR (qPCR)

RNA was extracted by TRIzol Reagent (Invitrogen), and cDNA was obtained by reverse transcription using the PrimeScript RT Reagent Kit with gDNA Eraser (Clontech). The qPCR was conducted with TB Green Premix Ex Taq (Clontech) on a Quantagene q225 Real-Time PCR System (Kubotechnology). The primers are listed in the Supplemental Table 1.

### Co-immunoprecipitation (co-IP) assay

The co-IP assay was performed as described in our previous reports [22, 24]. Briefly, cells were collected and lysed in 500 μl of BC300 containing 50 mM Tris (pH 7.4), 300 mM KCl, 20% glycerol, 0.2 mM EDTA, 0.1% NP40, and 1× protease inhibitor cocktail (Invitrogen). After centrifuging at 12,000 × *g* for 10 min at 4 °C, the clarified lysates (400 μl) were transferred to a new tube and diluted with an equal volume of BC0 (BC300 without KCl), resulting in a total volume of 800 μl. One percent (1%) of the samples were saved as input. Co-IP was performed by gently rocking the diluted lysates with 10 μl of anti-Flag M2 magnetic bead slurry (Sigma). For endogenous co-IP, the sample was incubated with 2 μg of the indicated antibodies at 4 °C overnight, followed by thorough washes with 1 ml of BC150 (BC0 with 150 mM KCl). The samples were then incubated with 20 μl of protein A/G bead slurry for 2 h at 4 °C. After washing the beads with BC150 three times in the cold room, they were boiled in 50 μl of 1× SDS gel loading buffer, and the supernatant was used for western blot analysis. Unless otherwise noted, 5% of the input was loaded onto the gel. The original western blots were provided in the Supplemental Material.

### Protein purification and in vitro binding analysis

His-tagged human WIP1 was cloned into pET28a (Novagen), and Glutathione S-transferase (GST) tagged human AMPKα2 cDNA was cloned into pGEX-4T-1(Cytiva), respectively. The two recombinant plasmids were transformed into BL21 STAR DE3 E. coli cells (Invitrogen) individually and induced by Isopropyl β-D-1-thiogalactopyranoside (IPTG) in BL21 E. coli cells (Invitrogen) with the condition of 0.8 mM IPTG at 18 °C for 20 h. Cells were resuspended in 500 μl lysis buffer (50 mM Tris-HCl, pH 7.5, 100 mM NaCl, 1 mM DTT, 1 mM PMSF, and 1 × protease inhibitor) for 10 min on ice. After sonication, the mixture was clarified by centrifugation at 12,000 × *g* for 10 min at 4 °C. While the supernatant containing His-tagged WIP1 protein was purified with Ni-NTA resin (GE Healthcare) as described [25], the GST-tagged AMPKα2 was further captured by 100 μl anti-GST magnetic beads (MedChem Express). The beads were pre-incubated with 500 μl of binding buffer (50 mM Tris, pH 7.4, 300 mM NaCl, 10% Glycerol, 0.1% NP40) for 2 h at 4 °C. The mixture was washed extensively with binding buffer and then resuspended in 100 µl of binding buffer. This was followed by the addition of 500 ng of purified His-WIP1 and incubation at 4 °C overnight with rotation. The beads were then washed extensively with binding buffer, and the bound proteins were eluted in 60 µL of GST elution buffer (50 mM Tris-HCl, pH 8.5, 150 mM NaCl, 40 mM reduced glutathione) for 30 min at room temperature. The eluate was then mixed with an equal volume of 2 × SDS loading buffer and denatured at 100 °C for 10 min in a metal bath. SDS-PAGE and western blotting were used to verify the presence of the indicated proteins. The original western blots were provided in the Supplemental Material.

### ROS detection assay

The ROS detection assay was conducted by adding 5 μM 2’-7’-dichlorofluorescein diacetate (DCFDA) (Invitrogen) to the culture medium and incubating for 15 min as described [26]. The levels of ROS were detected by flow cytometry and quantified by FlowJo software.

### Generation of WIP1 knock-out cells by CRISPR-Cas9

The WIP1 knock-out (KO) MCF7 and HeLa cells were constructed as we recently described [24]. Briefly, eight guide RNAs (gRNAs) targeting the first exon of WIP1 were designed and cloned into pSpCas9(BB)-2A-GFP (Addgene #48138) for each. The genomic DNA was then isolated for sequencing 48 h after transfecting these plasmids into 293T cells. One of the gRNAs, which induces homozygous deletion of 13 bp (5’-AGTACATGGAGGA-3’) upstream of WIP1 T25, was chosen for further study. SgRNA sequences are provided in the supplemental Table 1. We selected 45 GFP-positive single colonies of MCF7 cells and 26 of HeLa cells for sequencing. One colony from each group (1/45 and 1/26), verified by sequencing, was further confirmed by western blotting.

We also confirmed the KO efficiency (count ratio of 99.62%) in MCF cells using next-generation sequencing (NGS) (Tsingke Biotech). Briefly, the genomic DNA was isolated and diluted to 10 ng/µl, and library construction was performed using the S-1.2x-TN5 library enrichment Mix (adapter and primer sequences are listed in the supplemental Table 1). After library preparation, the product was subjected to agarose gel electrophoresis, and the gel was excised for recovery. The recovered product was purified and sequenced using the MiSeq Reagent Micro Kit V2 and the MiSeq sequencer. Raw sequencing data were quality-controlled with fastp, filtering out low-quality reads or reads shorter than 15 bp. Subsequently, bcl2fastq was used for demultiplexing (data splitting).

### Comet assay

Cells were seeded into 6-well plates at a density of 1 × 10^5^ cells per well. Before or after IR exposure (4 Gy), the cells were harvested at the indicated time and then utilized for alkaline comet assays according to the manufacturer’s instructions (Cell Biolabs). When necessary, cells were treated with 2.5 µM GSK2830371 (Selleck, Cat #: S7573) for 2 h prior to IR exposure. For quantification, comets on each gel were observed using a fluorescence microscope. The degree of DNA damage was measured using CASP software and presented as % of tail DNA. Statistical analysis was performed from three biological replicates, with approximately 30 cells (1–2 microscope images) counted in each replicate, totaling around 100 cells.

### In vitro kinase and γH2AX dephosphorylating assay

The AMPK complex was isolated from 293T cells by overexpressing Flag-tagged AMPKα2. Cells were seeded in 10 cm dishes at 70–80% confluence and transfected with 10 μg of the Flag-AMPKα2 plasmid. After 24 h, the culture medium was replaced with glucose-free DMEM for 24 h to activate AMPK. Cells were then harvested by scraping into ice-cold PBS. The cell pellet was washed twice with PBS and lysed in 1 ml of ice-cold lysis buffer (50 mM Tris-HCl, pH 7.5, 150 mM NaCl, 1 mM EDTA, 1% Triton X-100, 1 mM DTT, and 1× protease/phosphatase inhibitor cocktail) for 30 min. Lysates were centrifuged at 12,000 × *g* for 10 min at 4 °C. The supernatant was incubated with 30 μl of anti-Flag M2 magnetic beads for 2 h at 4 °C with end-over-end rotation. Beads were washed three times with 1 ml of lysis buffer and twice with kinase buffer (Cell Signaling Technology, Cat #: 9802). The immunoprecipitates were resuspended in 50 μl of kinase buffer containing 0.5 mg of GST-Vec and 0.5–1 mg of GST-WIP1/GST-WIP1-T25A, and then incubated at 37 °C for 30 min. The kinase reactions were stopped by adding 50 μl of 2× SDS-PAGE loading buffer, and the samples were then analyzed using SDS-PAGE and western blotting.

We employed two strategies to conduct the in vitro γH2AX dephosphorylation assay. (1) We enriched Flag-tagged EGFP, WIPT WT, and T25A mutant from 293 T cells using a co-IP experiment with M2 beads as described [25]. Simultaneously, Myc-tagged H2AX (γH2AX) protein was obtained by transfecting the construct into 293 T cells and subjecting them to CPT treatment (10 µM, 1 h) before enrichment using anti-Myc IgG/A beads. The respective Flag-tagged proteins were then incubated with Myc-tagged H2AX (γH2AX); (2) GST-tagged WIP1 WT and T25A mutant proteins were expressed and purified from E. coli. Histones were isolated from 293 T cells, which were pre-treated with 10 µM CPT for 1 h to induce DNA damage, using the histone extraction kit following the manufacturer’s instructions (Proteintech, Cat #: PK10022). The immunoprecipitates (0.2–1 mg) were resuspended in 50 μl of phosphatase buffer (40 mM HEPES, pH 7.4, 100 mM NaCl, 50 mM KCl, 1 mM EGTA, and 50 mM MgCl_2_) containing 0.5 mg Myc-tagged H2AX or 0.5 mg histone extract, and incubated at 30 °C for 30 min. Subsequently, an equal volume of 2× SDS loading buffer was added to stop the reaction, and the proteins were analyzed using SDS-PAGE and western blotting.

### Salt fractionation of cell nuclei

Salt fractionation of cell nuclei was performed as described [27]. Briefly, cells were harvested and gently resuspended in 2 ml of buffer A [0.32 M sucrose, 60 mM KCl, 15 mM NaCl, 5 mM MgCl_2_, 0.1 mM EGTA, 15 mM Tris-Cl (pH 7.4), 0.5 mM DTT, 0.1 mM phenylmethylsulfonyl fluoride (PMSF), and 1 × protease inhibitor cocktail]. An equal volume of buffer B [0.32 M sucrose, 60 mM KCl, 15 mM NaCl, 5 mM MgCl_2_, 0.1 mM EGTA, 15 mM Tris-Cl (pH 7.4), and 0.1% NP-40] was added and incubated at 4 °C for 10 min. The mixture was then added slowly to the 10 ml of precooled buffer C [1.2 M sucrose, 60 mM KCl, 15 mM NaCl, 5 mM MgCl_2_, 0.1 mM EGTA, 15 mM Tris-Cl (pH 7.4), 0.5 mM DTT, 0.1 mM PMSF, and 1 × protease inhibitor cocktail] and centrifuged at 10,000 × *g* at 4 °C for 20 min. The pellet (nuclei) was then resuspended in 1 ml of buffer D [10 mM Tris-Cl (pH 7.4), 2 mM MgCl_2_, 5 mM CaCl_2_, and 0.1 mM PMSF] and was digested with 50 U of micrococcal nuclease (MNase) (Thermo Fisher Scientific) at 37 °C for 2 h. After washing with 1 ml of buffer D, the pellet was further resuspended with 200 μl of buffer E [10 mM Tris-Cl (pH 7.4), 2 mM MgCl_2_, 2 mM EGTA, 0.1% Triton X-100, and 0.1 mM PMSF] and incubated for 10 min at 4 °C with increasing concentrations of NaCl. The supernatant was collected by centrifugation at 500 × *g* for 1 min at 4 °C. The presence of the indicated proteins was verified using SDS-PAGE and Western blotting.

### Immunohistochemistry (IHC) staining

The IHC staining procedure was carried out as described in our recent report [28]. Briefly, the dewaxed tumor tissue sections were immersed in 10 M sodium citrate buffer (pH 6.0) and heated for 30 min to expose the antigen. The IHC kit (Zsbio, Beijing, China) was used according to the manufacturer’s recommendations. After coloring with DAB reagent, the nuclei were stained with hematoxylin. A fully automated slice scanning system detects protein expression in the tissue slices. The antibodies used for IHC staining target WIP1 (Cat #: 11901), AMPK (Cat #: 9751), and p-AMPK (Cat #: 2535), all of which were purchased from Cell Signaling Technology (CST). The anti-phosphorylated WIP1 (T25) was developed in our laboratory. Tumor regions were manually annotated, and a pathologist scored the staining intensity, assigning values of 0 (negative), 1 (weak positive), 2 (moderate positive), and 3 (strong positive). The positive cell ratio score was defined as: 1 (1–10% positive), 2 (11–50% positive), 3 (51–80% positive), and 4 (81–100% positive). The IHC score was calculated by multiplying the positive cell ratio score by the staining intensity. Correlation analyses were performed using GraphPad software.

### Crystal violet staining assay

Briefly, 500-1000 MCF7 or HeLa cells were plated per well in 6-well tissue culture plates. If necessary, the cells were irradiated with X-rays (6 Gy) on the 6th day after seeding and kept in culture for an additional 10 days. At times or treatments indicated, cells were washed with PBS and fixed for 10 min in a 10% formalin solution. Cells were then rinsed with distilled water and stained with 100 ml of 0.1% crystal violet solution for 30 min. Stained cells were rinsed with water to remove the excess traces of the staining solution. The colony number or size was recorded.

### Xenografted tumor models and X-ray irradiation

The MCF7 or HeLa cells (5 × 10^6^) were suspended in 200 μl of PBS and then injected subcutaneously into 4-week-old female nude mice. The mice with tumors measuring ~0.1 cm in diameter were randomly and blindly divided into four groups (*n* = 5 per group). Two groups received five local X-ray treatments with a dose of 2 Gy every other day, starting the 6th day after implantation. The other two groups served as the control. Following the completion of the experiment, the mice were euthanized, and their tumors were collected and analyzed. The tumor volume was calculated using the formula (length × width × height)/2.

### Statistical analysis

Experimental results were obtained from biological replicates and averaged to generate a single value, which is recorded as the mean ± SD or +SD under each condition. Two or three experimental groups were compared using the Student’s *t*-test or the Student’s *t*-test with Welch’s correction for unpaired data. More than two comparisons were compared using one-way ANOVA with Bonferroni’s correction. Statistical significance was determined at *P* < 0.05 (*); *P* < 0.01 (**); *P* < 0.001 (***). Other statistical details were indicated in the figure legends.

## Results

### Activation of AMPK promotes dephosphorylation of γH2AX

**Fig. 1:**
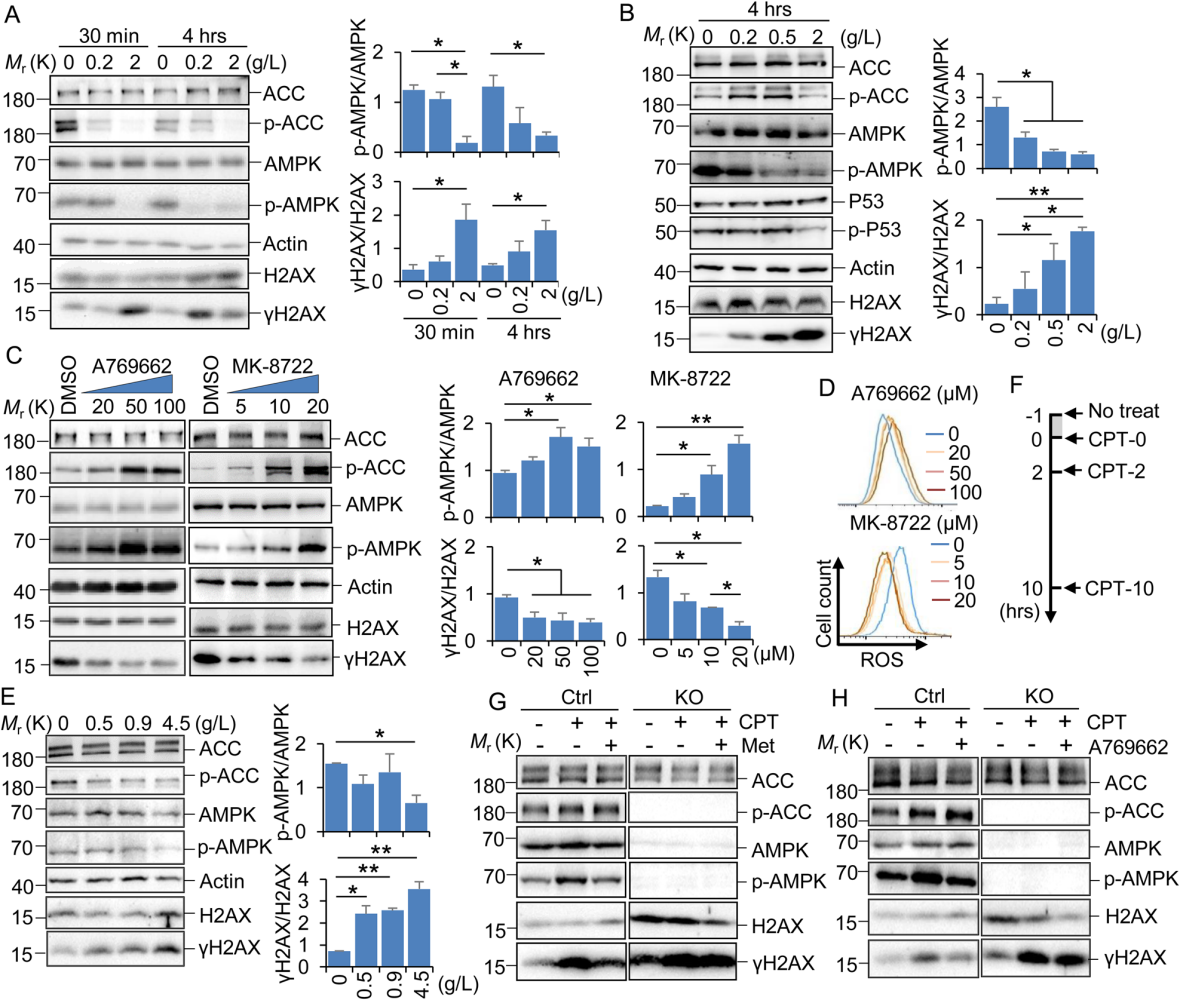
Activation of AMPK reduced γH2AX levels. **A**, **B** MOLM13 (**A**) and 293 T (**B**) cells were incubated with increasing doses of glucose in RPMI 1640 and DMEM medium containing 10% FBS, respectively. Cells were harvested at different time points. **C** MOLM13 cells were incubated with vehicle or increasing doses of A769662 (μM) and MK-8722 (μM) for 24 h. **D** The ROS level in MOLM13 cells shown in (**C**) was detected by flow cytometry. **E** MEF cells were incubated with increasing doses of glucose in DMEM medium containing 10% FBS for 24 h. **F** Cell treatment protocol. Cells were incubated with 5 µM CPT for 1 h in culture medium and then maintained in fresh medium for the indicated times after complete washing to remove residual CPT. **G**, **H** Control and AMPKa1/a2 double KO MEF cells were treated with 0.5 mM Met (**G**) and 50 μM A769662 (**H**) for 24 h, respectively. The cells were then harvested for analysis after being treated with (CPT-2) or not with CPT(DMSO). **A**–**C**, **E**, **G**, **H** SDS-PAGE and western blotting detected the indicated proteins. Two to three biological replicates were performed for each experiment, and a representative image was presented. **A**–**C**, **E** Quantification was performed with ImageJ from two to three biological repeats. Data were presented as mean + SD (**A**–**C**, **E**). *P < 0.05, **P < 0.01, by 1-factor ANOVA with a post hoc *t*-test. Insignificant comparison (n.s) was not presented.

It has been reported that high glucose levels are associated with increased DNA damage, as evidenced by γH2AX levels [29, 30]. To monitor the effect of glucose on DNA damage, we treated MOLM13 (Fig. 1A) and THP1 (Fig. S1A) leukemia cells with varying doses of glucose in RPMI medium. We found that γH2AX was rapidly reduced under the no-glucose or low-glucose conditions. It is well-known that AMPK serves as an energy sensor in mammals. Consistent with the previous report that AMPK can be activated by glucose deprivation within 15 min [31], glucose starvation rapidly activated AMPK (Figs. 1A and S1A). A negative correlation between active AMPK and γH2AX was consistently observed in adherent 293 T and MCF7 cells (Figs. 1B and S1B), suggesting that this relationship is not specific to cell type. Additionally, we demonstrated that glucose levels have a minor effect on DNA damage (Fig. S1C). To further verify the correlation between activated AMPK and γH2AX, MOLM13 cells were treated with increasing concentrations of A769662 and MK-8722 for 24 h to activate AMPK (Fig. 1C). We found that both A769662 and MK-8722 can activate AMPK and inhibit the protein level of γH2AX in a dose-dependent manner. Because ROS is one of the primary sources of DNA damage, we measured ROS production spontaneously in the above treatments. We found that A769662 slightly increased cellular ROS levels, whereas MK-8722 reduced ROS levels in a dose-dependent manner, suggesting that ROS is not the primary cause of AMPK-mediated dephosphorylation of γH2AX (Fig. 1D).

To determine if glucose deprivation and activator treatments that inhibit γH2AX are AMPK-dependent, we repeated these treatments in MEFs with a double KO of the two catalytic subunits of AMPK, α1 and α2 (AMPKa1/a2 KO), as described elsewhere [20]. The efficiency of KO and the response to the topoisomerase I inhibitor camptothecin (CPT) were validated before the treatments (Fig. S1D). Consistent with the phenomenon observed in Fig. 1A, no or low-level glucose reduced γH2AX in control MEFs but not in AMPKa1/a2 KO cells (Figs. 1E and S1E). Moreover, we found that the CPT treatment induced γH2AX compared with vehicle (DMSO), and the presence of metformin and A769662 significantly reduced γH2AX levels in control MEFs (Fig. 1F–H). In contrast, the γH2AX level enhanced by CPT was not reduced by metformin or A769662 in AMPK KO cells (Fig. 1F–H). Additionally, the treatment with A769662 decreases γH2AX level in a dose-dependent manner in control cells, while having only a minor effect in AMPK KO cells (Fig. S1F). Collectively, these results indicate that activation of AMPK promotes the dephosphorylation of γH2AX.

### WIP1 is the primary phosphatase responsible for maintaining the γH2AX level

**Fig. 2:**
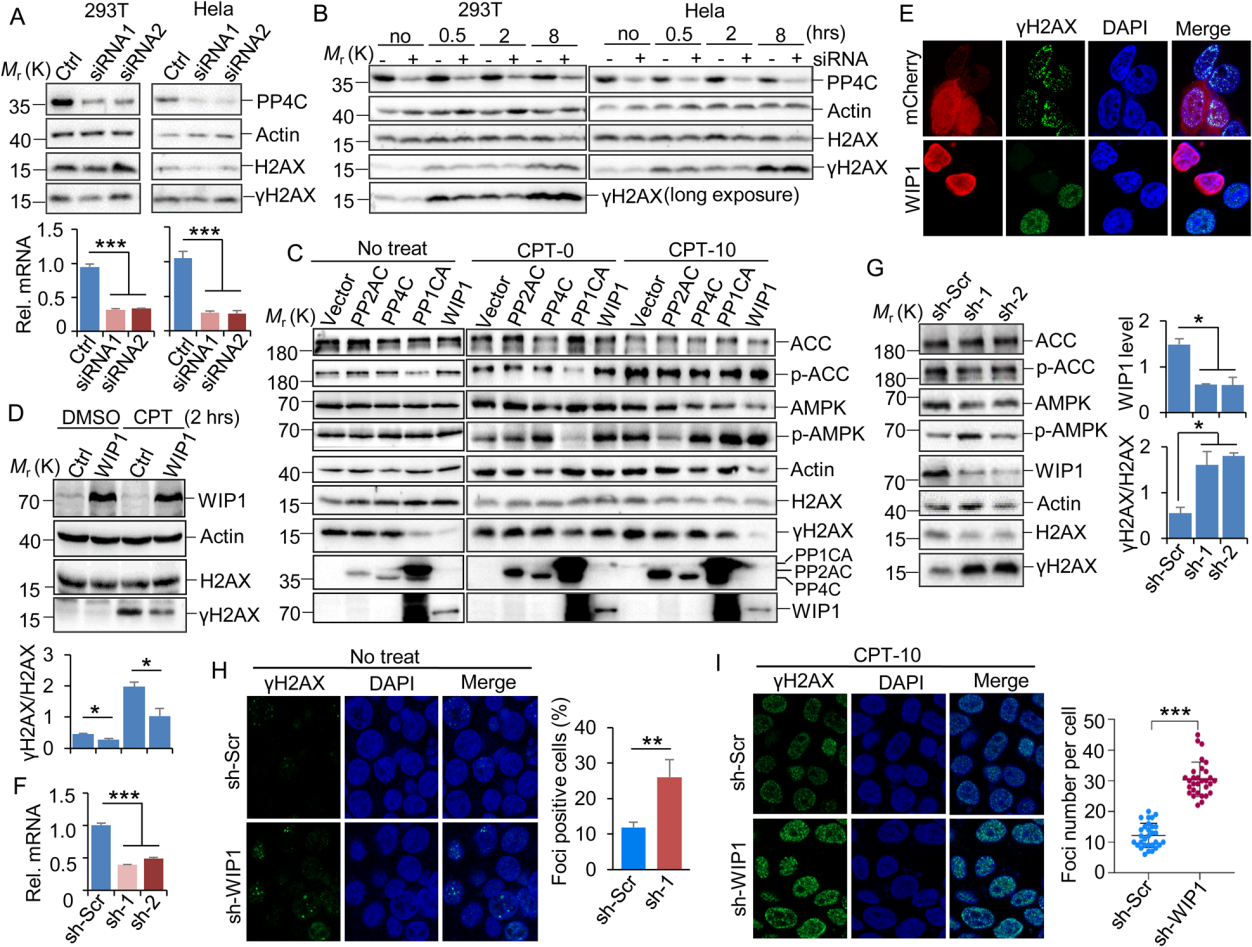
WIP1 dephosphorylates γH2AX in intact and damaged conditions. **A** 293 T (left) and HeLa (right) cells were transfected with control or PP4C siRNAs and harvested after 48 h. Two different siRNAs targeting PP4C were used individually. **B** 293 T (left) or HeLa (right) cells transfected with control or PP4C siRNA (siRNA-1) were further treated or not with CPT (5 µM), and cells were harvested at the indicated times for analysis. **C** Flag-tagged control (Vector), PP2AC, PP4C, PP1CA, and WIP1 were transfected into 293 T cells. The cells were further treated or not with CPT (5 µM) for the indicated times at 48 h post-transfection. **D** MEF cells were infected with lentivirus expressing either a control or a mouse WIP1 fragment. The cells were treated with DMSO and CPT (5 µM, 2 h). The indicated proteins were detected by western blotting. Quantification (bottom) was performed with ImageJ from 2 biological repeats. **E** 293 T cells transfected with mCherry control or mCherry fused WIP1 were further treated with CPT (5 µM, 2 h), and γH2AX foci were visualized by IF staining. **F**, **G** HeLa cells were transduced with shRNA-scramble (sh-Scr) or shRNA-WIP1 lentivirus and then selected by puromycin for three days. Indicated RT-PCR and western blotting detected mRNA (**F**) and protein (**G**) levels. **G** Quantification (right) was performed with ImageJ from 3 biological repeats. **H**, **I** Control or WIP1 KD HeLa cells were treated with DMSO (H) and CPT (5 µM) (**I**), respectively. The γH2AX foci were visualized (left) and analyzed (right) by IF staining at the indicated times post-treatment. **A**–**C**, **F** SDS-PAGE and western blotting detected the indicated proteins. Two to three biological replicates were performed for each experiment, and a representative image was presented. Data were presented as mean + SD (**A**, **D**, **F**–**H**) and ± SD (**I**). *P < 0.05, **P < 0.01, and ***P < 0.001, by 1-factor ANOVA with a post hoc *t*-test. Insignificant comparison (n.s) was not presented.

We hypothesized that AMPK might promote the dephosphorylation of γH2AX by activating (phosphorylating) downstream phosphatases. Previous studies have shown that the phosphatase PP4C can dephosphorylate γH2AX, and silencing of PP4C in HeLa cells increased γH2AX in the presence or absence of exogenous DNA damage [7]. However, in our experiments, although the protein and mRNA levels of PP4C were reduced by transfection of identical siRNAs as described [7], γH2AX levels were not altered in either 293 T or HeLa cells (Fig. 2A). UV radiation-induced γH2AX levels were also not affected by silencing of PP4C (Fig. 2B). To identify the phosphatase responsible for dephosphorylation of γH2AX, we ectopically overexpressed HA-tagged PP2AC, PP4C, PP1CA, and WIP1 in 293 T cells with or without treatment with CPT. Overexpression of PP2CA and PP4C did not alter γH2AX levels under either normal or damaged conditions (Fig. 2C), even though we had demonstrated that AMPK can bind with PP2CA and PP4C (Fig. S2A, B). However, overexpression of PP1CA and particularly WIP1 significantly reduced the protein level of γH2AX under both normal (no treatment with CPT) and damaged conditions (CPT treatment) (Fig. 2C, D).

The regulatory subunits of PP1CA and PP2CA are crucial for enzyme activity and substrate recognition/binding [32–35]. We demonstrated that depleting PPP1R12A, a subunit of PP1C that was identified as an AMPK substrate [36], or subunits of PP2C (PPP2R5A-E) [34, 35], had little effect on γH2AX level (Fig. S2C–I). Furthermore, PP1CA inhibited the activation of AMPK soon after CPT treatment (CPT-0), whereas PP2AC acted later (CPT-10) (Fig. 2C). We did not observe any significant effect of PP4C and WIP1 on the phosphorylation of AMPK and its substrate ACC. Notably, IF staining revealed that γH2AX foci completely disappeared upon overexpression of WIP1 (Fig. 2E). Consistently, knocking down WIP1 using lentiviruses in HeLa cells (Fig. 2F, G) resulted in a significant increase in γH2AX levels in normal and damage conditions, as indicated by foci staining (Fig. 2H, I). These results suggest that WIP1, rather than PP2AC and PP4C, is primarily responsible for the dephosphorylation of γH2AX in normal and damaged conditions.

### WIP1 directly binds to AMPK and enhances the accumulation of activated AMPK in the nucleus

**Fig. 3:**
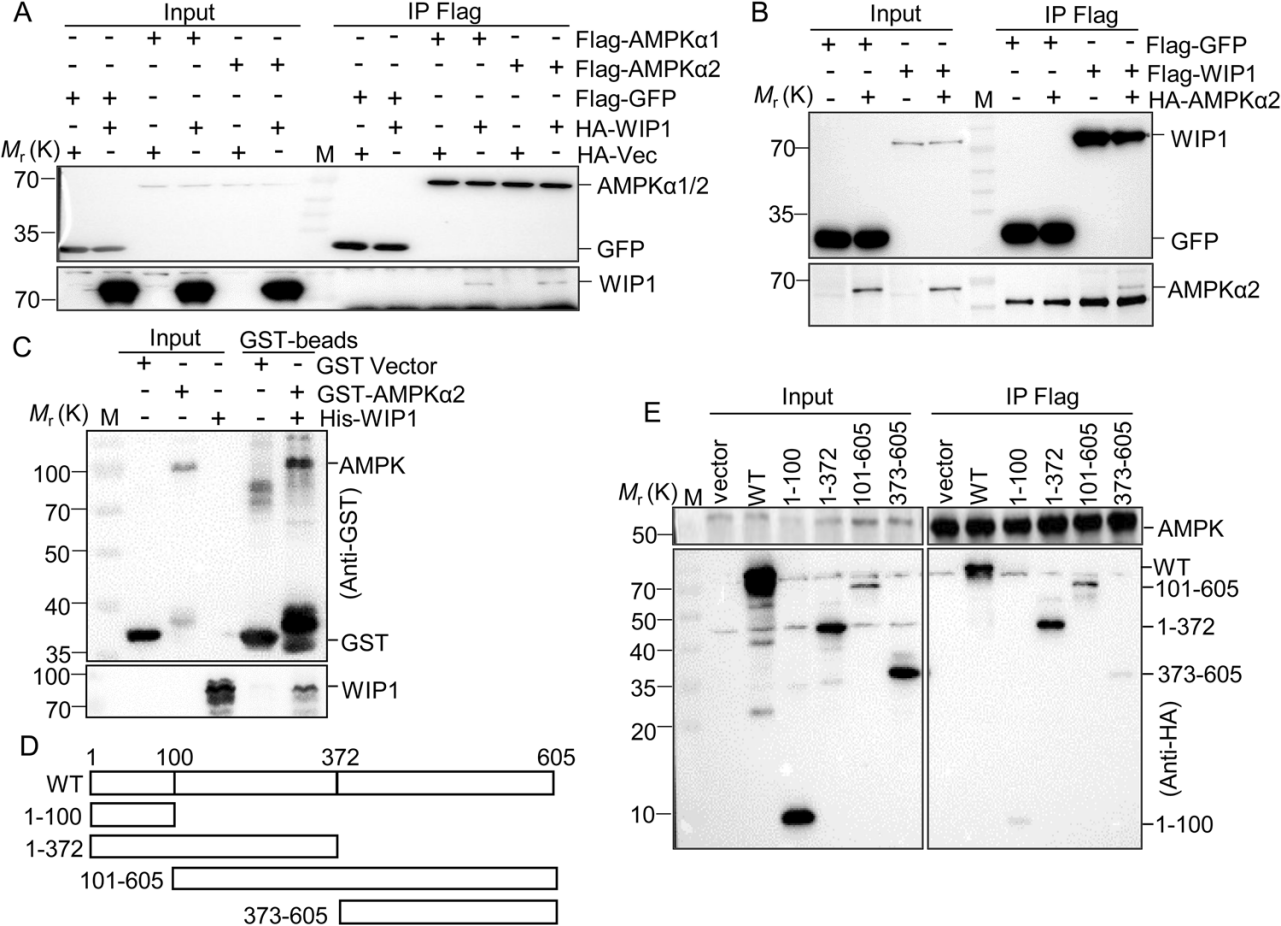
AMPKα directly binds to WIP1. **A** 293 T cells were transfected with individual Flag-tagged AMPKα1 and AMPKα2 alone or with HA-tagged WIP1. **B** 293 T cells were transfected with Flag-tagged GFP control, Flag-tagged WIP1, or in combination with HA-tagged AMPKα2. **A**, **B** Co-IPs were performed with anti-Flag M2 resin. **C** In vitro GST pull-down assay for GST bead-bound GST-AMPKα2 with His-WIP1. **D** Diagram for truncation mutants of WIP1. **E** 293 T cells were transfected with the indicated Flag-tagged AMPKα2 and individual HA-tagged WIP1 mutants or vector control. Co-IP was performed with anti-Flag M2 resin. **A**–**C**, **E** SDS-PAGE and western blotting detected the indicated proteins. Two to three biological replicates were performed for each experiment, and a representative image was presented.

To investigate whether AMPK-promoting dephosphorylation of γH2AX is dependent on WIP1, we performed a co-immunoprecipitation (co-IP) assay in 293 T cells. We found that WIP1 was recovered by both Flag-tagged AMPKα1 and AMPKα2, but not by the vector control, indicating that the shared domain of AMPKα1 and AMPKα2 is responsible for this interaction (Fig. 3A). Since the expression level of AMPKα2 was higher than that of AMPKα1 (data not shown), the AMPKα2 construct was chosen for subsequent experiments. Reciprocal co-IP experiments confirmed the association (Fig. 3B). Additionally, glucose has a limited effect on the interaction between AMPK and WIP1 (Fig. S3A). Furthermore, the in vitro GST pulldown experiment validated the direct interaction between AMPKα2 and WIP1 (Fig. 3C). WIP1 comprises the catalytic domain (N-terminal) and the regulatory domain (C-terminal) [37, 38]. The domain mapping experiments indicated that fragments 1–100 aa and 373–605 aa of WIP1 did not interact with AMPKα2, while both 1–372 aa and 101–605 aa exhibited strong interactions (Fig. 3D, E), suggesting that the WIP1 101–372 aa is crucial for its interaction with AMPK. Moreover, the fragment of WIP1 101–605 aa showed a weak enzyme activity towards γH2AX compared with the entire length of WIP1, suggesting that the WIP1 1–100 aa is critical for its enzyme activity (Fig. S3B).

AMPK shuttles between the nucleus and cytoplasm to regulate various pathways [39]. WIP1 is mainly a nuclear protein, and its interaction with AMPK prompts us to investigate the effect of WIP1 on the subcellular localization of AMPK. We, therefore, overexpressed or knocked out (KO) WIP1 in 293 T and MCF7 cells, respectively, and performed subcellular fractionation (Fig. S4). We found that overexpression of WIP1 has little effect on the subcellular localization of total AMPK but significantly increases the accumulation of activated AMPK in the nucleus (Fig. S4A). Consistently, WIP1 KO reduced the distribution of activated AMPK in the nucleus (Fig. S4B). These results suggest that WIP1 binds to AMPK and accumulates activated AMPK in the nucleus.

### Thr25 phosphorylation by AMPK increases the phosphatase activity of WIP1 towards γH2AX

**Fig. 4:**
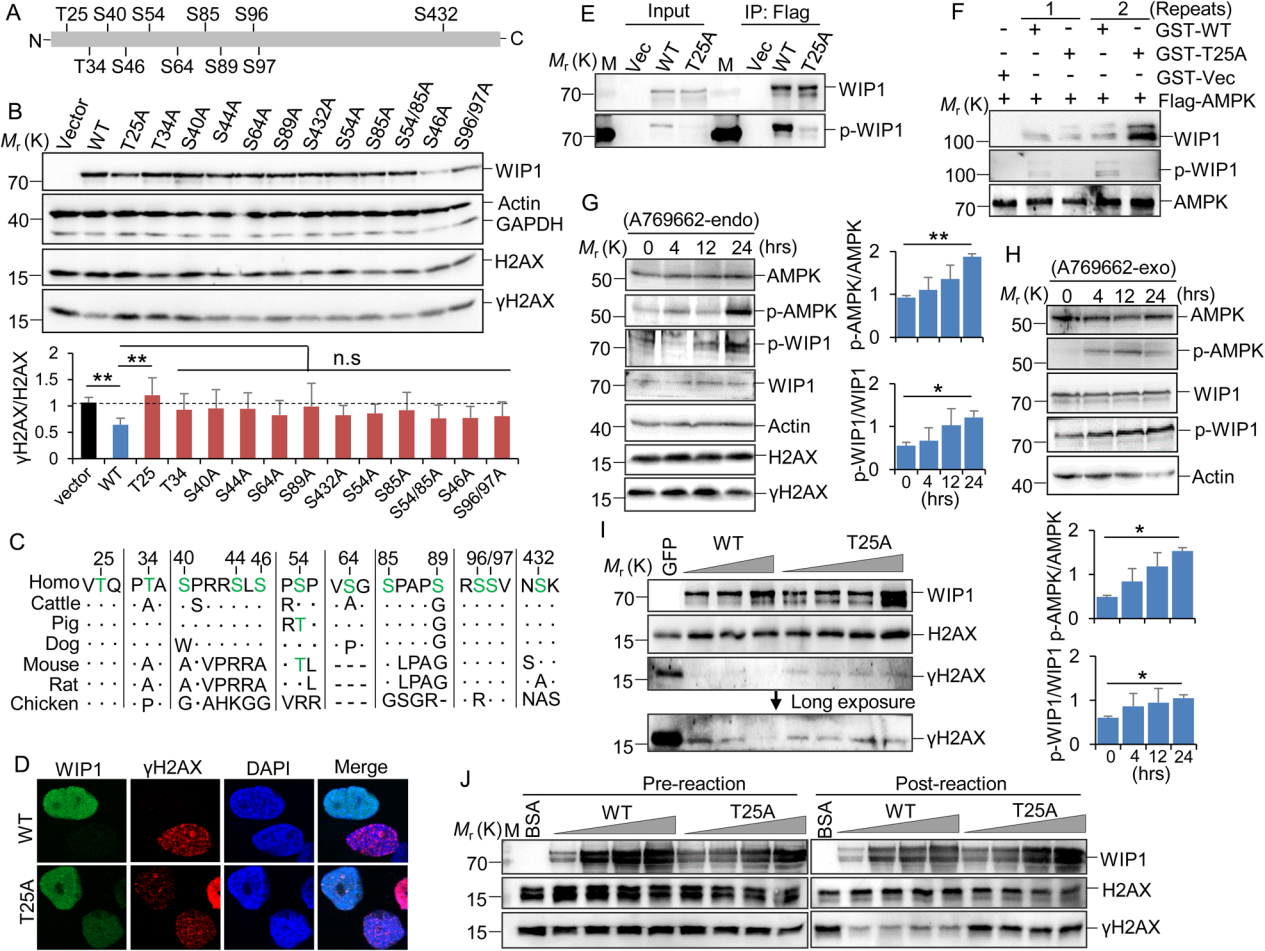
AMPK phosphorylates WIP1 at Thr25. **A** Diagram of phosphorylation sites on WIP1 identified by mass spectrometry. **B** 293 T cells were transfected with the indicated Flag-tagged WIP1 mutants or vector control. Cells were harvested 48 h post-transfection, and western blotting (top) detected the indicated protein level. The ratio of γH2AX vs H2AX was calculated from 3–4 biological repeats (bottom). **C** Protein sequence alignment of WIP1 among vertebrate species. **D** WIP1 KO MCF7 cells were infected with lentivirus expressing GFP-fused WIP1 WT and T25A mutant, respectively. Cells were further incubated with CPT (5 µM, 2 h) to induce DNA damage. WIP1 (green) and γH2AX foci (red) were visualized through IF staining and a fluorescence microscope. **E** Flag-tagged WIP1 WT and T25A were transfected into 293 T cells. The total and phosphorylated WIP1 (T25) levels were detected by western blotting, respectively. **F** Evaluation of the phosphorylation of WIP1 by AMPK kinase assay in vitro. Flag-tagged AMPKα2 (complex) was isolated from 293 T cells by M2 beads. **G** MCF7 cells were incubated with A769662 for the indicated time, and endogenous protein level (endo) was detected by western blotting. Quantification (right) was performed with ImageJ from 2 biological repeats. **H** 293 T cells were transfected with an equal amount of Flag-tagged WIP1 and then incubated with A769662 for the indicated time. Western blotting was used to detect protein expression, and quantification (bottom) was performed using ImageJ from 2 biological repeats. **I**, **J** In vitro dephosphorylating assay to evaluate the effect of WIP1 WT and T25A mutant on γH2AX, respectively. **I** Flag-tagged GFP, WIP1 WT, and T25A were incubated with Myc-tagged H2AX (γH2AX) in vitro, respectively. The tagged proteins were isolated from 293 T cells by M2 beads. **J** GST-tagged WIP1 WT and T25A isolated from E.coli were separately incubated with endogenous histones extracted from 293 T cells. Triangles represent an increasing dose of WIP1 WT and T25A mutant. **D**–**J** Two to three biological replicates were performed for each experiment, and a representative image was presented. Data were presented as mean + SD (**B**, **G**, **H**). *P < 0.05, **P < 0.01, by 1-factor ANOVA with a post hoc *t*-test. Insignificant comparison (n.s) was not presented.

To investigate whether AMPK phosphorylates WIP1, we conducted a comprehensive mass spectrometric (MS) analysis of phosphorylation sites using highly enriched, Flag-tagged WIP1 captured by M2 magnetic beads (Fig. S5A). Multiple phosphorylation sites on WIP1 were identified, including Ser54 and Ser85, which were previously reported (Figs. 4A and S5B–K) [40]. We then constructed point mutations of WIP1 that covered all phosphorylated sites identified by MS and transfected each of these constructs into 293 T cells. We found that the γH2AX level was reduced to approximately half by overexpression of wild-type WIP1 (WT) compared with the vector control and T25A mutant (Fig. 4B). Moreover, sequence alignment showed that T25 was highly conserved in different species (Fig. 4C). These results suggest that WIP1 T25 may be necessary for maintaining the phosphatase activity of WIP1 towards γH2AX.

Consistent with the result shown in Fig. 2E, overexpression of WIP1 WT, but not T25A mutant, prevented the formation of γH2AX foci (Fig. 4D). To investigate whether AMPK phosphorylates WIP1 at T25, we developed a rabbit polyclonal antibody that could specifically recognize WIP1 T25 phosphorylation (Fig. 4E). The in vitro kinase assay revealed that after incubation with the AMPK complex, a phosphorylation signal could be detected only on the WIP1 WT, but not on the T25A mutant (Fig. 4F). Furthermore, we observed that activation of AMPK by A769662 significantly increased phosphorylation signals on both endogenous and exogenous WIP1 (Fig. 4G, H). Additionally, the in vitro dephosphorylating assays confirmed that the dephosphorylating ability of the T25A mutant towards γH2AX is significantly reduced compared to WIP1 WT (Fig. 4I, J). Notably, the T25D phosphomimetic mutant exhibited enhanced enzymatic activity towards γH2AX compared with WIP1 WT (Fig. S6A, B). These findings indicate that AMPK can directly phosphorylate WIP1 at Thr25, reducing γH2AX levels.

### The phosphorylation of Thr25 by AMPK increases WIP1 stability and enhances its binding affinity with γH2AX

**Fig. 5:**
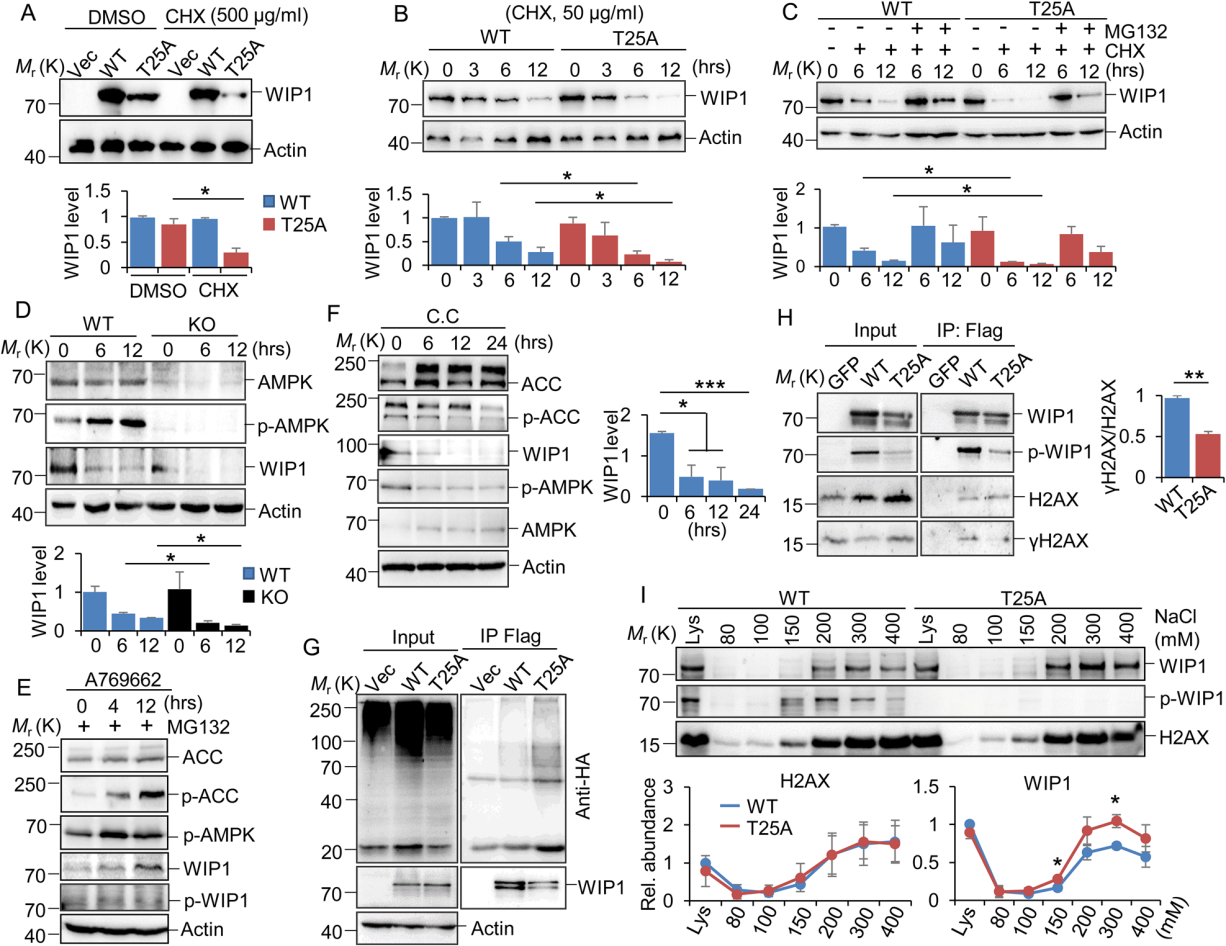
AMPK phosphorylates WIP1 Thr25, enhancing its protein stability and binding affinity with γH2AX. **A**–**C** 293 T cells were transfected with WIP1 WT and T25A expression constructs, respectively. The cells were then treated with 500 µg/ml CHX for 3 h (**A**) or 50 µg/ml CHX in the absence (**B**) and presence of 20 µM MG132 (**C**) for the indicated times. Quantification (bottom) was performed with ImageJ from 3 biological repeats. **D** Control and AMPKa1/a2 double KO MEF cells were incubated with 50 µg/ml CHX for the indicated times. Quantification (bottom) was performed with ImageJ from 2 biological repeats. **E**, **F** MCF7 cells were incubated with A769662 in the presence of MG132 (**E**) and C.C (**F**) for the indicated times to activate and inhibit AMPK activity, respectively. Quantification (**F**, right) was performed with ImageJ from 2 biological repeats. **G** 293 T cells were transfected with HA-tagged ubiquitin and Flag-tagged vector, WIP1 WT and T25A, respectively. Co-IPs were performed using anti-Flag M2 resin. Three biological replicates were performed, and a representative image was presented. **H** 293 T cells were transfected with plasmids expressing Flag-tagged GFP, WIP1 WT, and T25A. The cells were further incubated with 20 µM MG132 for 4 h and harvested for co-IP experiments. Quantification was performed with ImageJ from 3 biological repeats. **I** Differential salt fractionation of nuclei was used to analyze chromatin-associated WIP1 proteins. WIP1 WT or T25A mutant cells were digested with MNase and extracted with various salt concentrations for immunoblotting with the indicated antibodies. The relative expression levels were quantified using ImageJ software, with the protein expression values in the lysis set to 1. Data are from two biological repeats. Data are presented as mean + SD (**A**–**D**, **F**–**H**) or ± SD (**I**). *P < 0.05, **P < 0.01, and ***P < 0.001, by 1-factor ANOVA with a post hoc *t*-test. Insignificant comparison (n.s) was not presented.

The cellular WIP1 is maintained at a low level by proteasomal degradation [40]. To investigate the impact of WIP1 phosphorylation at T25 on protein stability, WIP1 WT and T25A were overexpressed and treated with varying cycloheximide (CHX) concentrations for different durations in 293 T cells. We observed a significant decrease in the levels of the WIP1 T25 A protein following treatment with high or low concentrations of CHX, compared to the WT protein levels (Fig. 5A, B). The decrease in both WIP1 WT and T25A after CHX treatment was significantly reversed by MG132 (Fig. 5C). Additionally, we consistently observed a more rapid degradation of WIP1 in AMPK KO MEFs compared to the control cells (Fig. 5D). This suggests that AMPK may maintain WIP1 stability through phosphorylation at T25. To examine whether activating AMPK can prevent the breakdown of WIP1, we measured the levels of endogenous WIP1 protein in the presence of an AMPK activator and inhibitor, respectively (Fig. 5E, F). The activation of AMPK by A769662 notably increased the total and phosphorylated WIP1 protein content (Fig. 5E). Conversely, an AMPK inhibitor, compound C (C.C), reversed this effect (Fig. 5F). Moreover, we observed that WIP1 T25A underwent more polyubiquitination than WT, indicating increased degradation of T25A (Fig. 5G).

WIP1 directly dephosphorylates γH2AX in mammalian cells [10]. AMPK-mediated Thr25 phosphorylation likely promotes the interaction between WIP1 and its substrate, γH2AX. To test this hypothesis, we measured the binding ability of WIP1 WT and the T25A mutant to H2AX/γH2AX in 293 T cells. The results showed that T25A could bind similar amounts of H2AX but bind a lower amount of γH2AX than WIP1 WT, suggesting that WIP1 T25 is essential for interaction with γH2AX (Fig. 5H). Additionally, the formation of γH2AX serves as a platform for recruiting DDR factors to the damage sites on chromatin. To compare the chromatin binding capacity of WT and T25A, we performed differential salt fractionation of nuclei from 293 T cells. We found that at the same salt concentrations as WIP1 WT, more WIP1 T25A mutant was extracted from the insoluble chromatin fractions (Fig. 5I). In contrast, similar amounts of H2AX were removed in WT and T25A-expressing cells, suggesting that WIP1 T25A had a weaker association with chromatin, likely due to the impaired interaction with γH2AX (Fig. 5I). Taken together, these results indicate that AMPK phosphorylates WIP1 at Thr25. This phosphorylation may increase the stability of the WIP1 protein and also enhance the interaction between WIP1 and γH2AX, contributing to efficient dephosphorylation of γH2AX.

### WIP1 Thr25 is critical for DSB repair and radioresistance of cancer cells

**Fig. 6:**
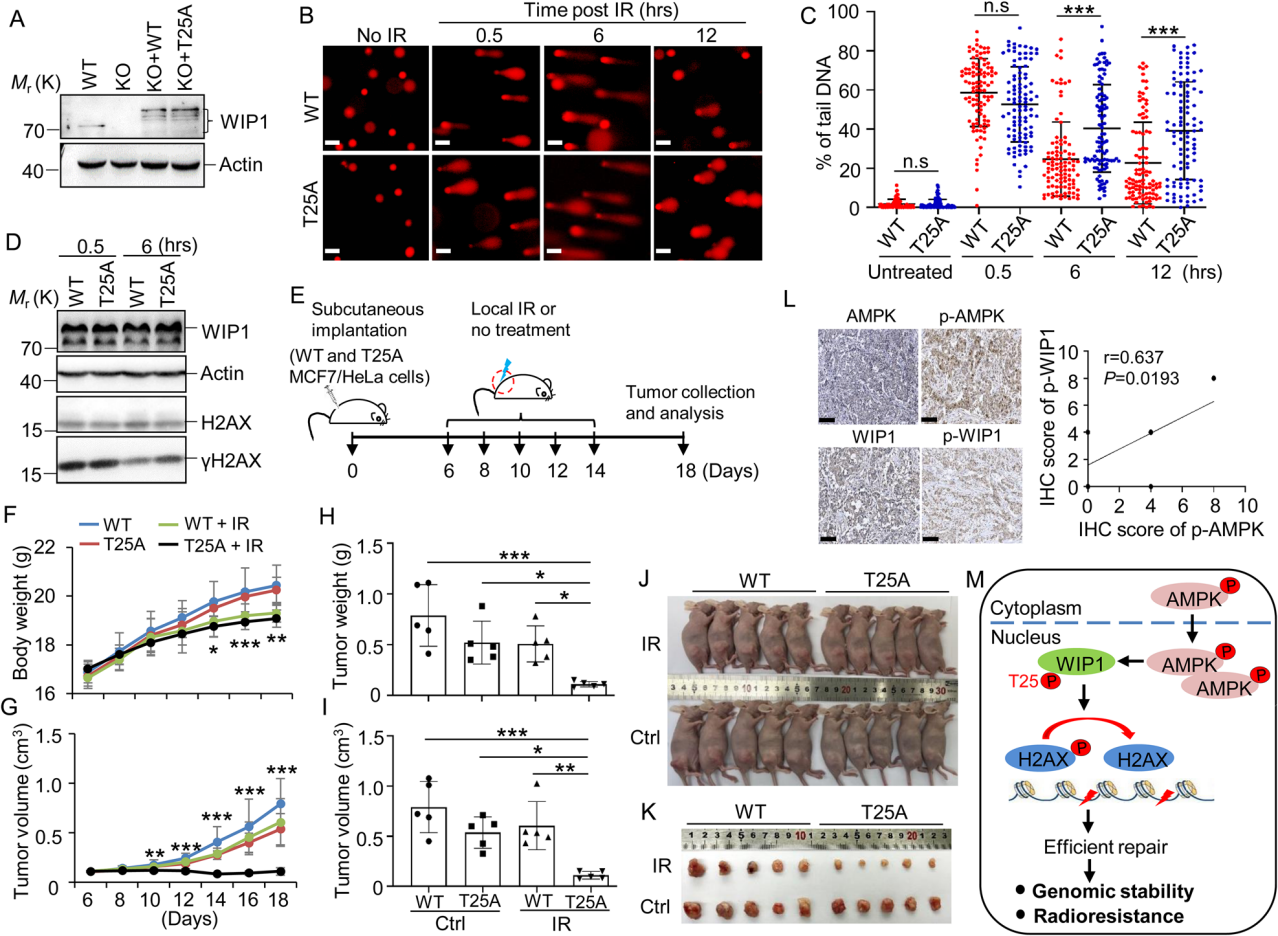
WIP1 Thr25 phosphorylation promotes DSB repair and contributes to the radioresistance of cancer cells. **A** The expression of WIP1 in the indicated cells. **B**–**D** The mCherry-fused WT and T25A mutant of WIP1 were reintroduced into WIP1 KO cells via lentiviral infection. After antibiotic selection, the positive infected cells were used for the comet assay (**B**, **C**) and western blotting (**D**). **B**, **C** The cells were exposed to IR, and the representative comet tails were presented. Scale bars, 20 μm. Quantification from three biological repeats with a total of 100 counts per treatment. **E** Scheme for the animal experiments. WIP1 KO MCF7/HeLa cells rescued by mCherry fused WT and T25A mutant were subcutaneously injected into nude mice. The mice were either treated or not with local IR exposure on the 6th day after injection. **F**–**I** Mice’s body weight (**F**) and tumor volume (**G**) were measured every two days until the end of the experiment. **H**–**K** Mice were killed, and the tumor weight (**H**) and size (**I**) were measured. (**J**, **K**) Representative pictures of xenograft tumors in response to the indicated treatment. *n* = 5 animals per group. **L** Protein levels in human breast cancer tissues. Representative IHC-staining images from a single tissue are shown (scale bar, 100 µm). Correlation analysis was conducted on 13 samples using GraphPad software. **M** A model for the role of AMPK in DNA repair in cancer cells: AMPK phosphorylates WIP1 at Thr25, increasing its protein stability and binding affinity with γH2AX. These processes enhance the dephosphorylation activity of WIP1, promoting the effective dephosphorylation of γH2AX and facilitating efficient DSB repair, thereby contributing to radioresistance. Data were presented as mean ± SD (**C**, **F**–**I**). *P < 0.05, **P < 0.01, ***P < 0.001 by 1-factor ANOVA with a post hoc *t*-test. Insignificant comparison (n.s) was not presented.

Enforced expression of WIP1 dephosphorylates γH2AX excessively and delays DSB repair [10], suggesting that the right amount of WIP1 is critical for effective DSB repair. Given that silencing WIP1 led to sustained γH2AX foci (Fig. 2I), and the enzyme activity of the T25A mutant is significantly reduced compared to WIP1 WT (Fig. 4I-J), we hypothesized that the WIP1 T25A mutant would also impede DNA repair. To test this, we generated WIP1 KO cell lines and reintroduced the full-length WIP1 (WT) and the T25A mutant into the KO cells, respectively (Figs. S8A, B and 6A). Using the comet assay, we measured the level of DSBs at multiple time points after X-ray irradiation (IR). The results showed that while comet tails were similar at the early time point (0.5 h) after IR exposure, the T25A mutant cells exhibited significant comet tails at 6 and 12 h after IR exposure compared to the WT (Fig. 6B, C). The γH2AX levels were similar between WT and T25A at 0.5 h post-IR, but T25A cells exhibited sustained γH2AX at 6 h, signifying impaired DSB repair (Fig. 6D). Similarly, in WIP1 KO MCF7 cells expressing the enzymatically inactive WIP1-D314A mutant (Fig. S8C) or WT cells treated with WIP1 inhibitor (Fig. S8D), comet tails remained elevated at 12 h post-IR, confirming that WIP1’s phosphatase activity is crucial for efficient DSB repair (Fig. S8C, D). These findings suggest that WIP1 Thr25, which is essential for its enzymatic activity, plays a key role in the effective repair of DSBs.

The WIP1 gene is often overexpressed or amplified in various cancers, including breast cancer cells (Fig. S7A) [41, 42], and is linked to poor survival outcomes (Fig. S7B). Our study shows that silencing WIP1 significantly reduces breast cancer cell proliferation (Fig. S8E–G). WIP1 KO and rescue experiments with either wild-type (WT) or T25A mutant in MCF7 (Fig. S8H–J) and HeLa (Fig. S8K, L) cells demonstrate WIP1’s essential role in cell survival, especially under stress conditions like IR exposure. Cells lacking WIP1 or rescued with T25A are more susceptible to IR and form smaller colonies, highlighting that the WIP1 T25 site is crucial for tumor growth and radioresistance.

To investigate the effects of the WIP1 Thr25 site on tumor cell growth in vivo, we reintroduced WIP1 WT and T25A into WIP1 KO MCF7 and HeLa cells, respectively. Then, we examined their tumor-forming ability in a xenograft model (CDX) (Fig. 6E). Animals were exposed to local IR, and their tumor growth was monitored. We observed that IR exposure reduced the body weight of the mice two weeks after implantation (Figs. 6F and S9A). Additionally, it significantly inhibited the tumor growth of T25A cells but had little effect on the WT cells (Figs. 6G–K and S9B–E). The results also showed that, without IR exposure, the weight and size of tumors derived from MCF7 cells decreased slightly (although the HeLa cell-derived tumors showed a significant reduction). However, after irradiation, T25A tumors were significantly inhibited in both the MCF7 and HeLa cell xenograft models (Figs. 6G–K and S9B–E). These findings suggest that WIP1 Thr25 plays a crucial role in sensitivity to IR exposure and promotes the proliferation of breast cancer cells.

To establish the relationship between AMPK activity and WIP1 phosphorylation at T25, we conducted a thorough analysis of their expression levels in MCF7 cell-derived xenograft mouse tumor tissues through IHC staining. We observed a strong presence of WIP1 phosphorylation at T25 in WT tumor tissues, while such signals were notably absent in T25A mutant tissues (Fig. S10A–E). Although initial results did not show a significant correlation between AMPK activity and WIP1-T25 phosphorylation (Fig. S10F), likely due to limited sample size, compelling evidence from clinical patient samples demonstrated a significant correlation (Fig. 6L). These findings strongly support the hypothesis that AMPK activity is closely linked to WIP1 phosphorylation at T25, highlighting its potential importance in tumor biology.

## Discussion

Phosphorylation of H2AX at serine 139 (γH2AX), one of the earliest signals in the DDR, is essential for effective DNA repair. However, after the completion of DNA repair, it is critical to remove or dephosphorylate γH2AX from the damaged site to ensure the efficient completion or initiation of new repair [7, 43]. Optimal γH2AX levels facilitate efficient foci formation, the accurate recruitment of repair factors, timely chromatin remodeling, and the appropriate selection of pathways [43, 44]. Our data elucidate how the AMPK-WIP1 pathway maintains this critical balance to promote radioresistance: (1) Under normal conditions, AMPK activation reduces total γH2AX protein (Fig. 1A) without affecting γH2AX foci number (Fig. S1C). This indicates AMPK specifically promotes dephosphorylation of non-damage-associated γH2AX. (2) Post-damage: following IR, WIP1 WT and T25A mutant cells initially show similar DNA damage (comet tails at 0.5 h; Fig. 6B, C). However, T25A mutants exhibit delayed repair, with persistent foci at 12 h (Fig. 6B, C). This cellular delay aligns with our in vivo finding that WIP1 WT tumors are more radioresistant than T25A mutant tumors (Fig. 6F–K). Therefore, the AMPK-WIP1 pathway promotes radioresistance not by broadly suppressing γH2AX, but through precise spatiotemporal regulation: it removes non-damage-related γH2AX under normal conditions and ensures timely dephosphorylation of damage-related γH2AX after repair. This dynamic control maintains optimal γH2AX levels, allowing for efficient repair while preventing the harmful effects of either insufficient signaling or persistent damage signals, ultimately improving cell survival after irradiation. Moreover, we note that WIP1 has been reported to promote homologous recombination independently of γH2AX by dephosphorylating other substrates [45], which increases complexity and significance in studying WIP1’s role. Although T25A mutant cells exhibit both persistent γH2AX foci and unresolved DNA damage, it is important to recognize that retention of γH2AX may also reflect repair deficiencies unrelated to WIP1-mediated dephosphorylation.

The phosphatases responsible for the efficient dephosphorylation of γH2AX are still controversial [7–10]. Previous studies suggest that PP2AC and PP4C can dephosphorylate γH2AX under stress and intact conditions, respectively [7, 8]. However, in our system, silencing or ectopic expression of PP2AC/PP4C had little effect on γH2AX levels under intact or damaged conditions. Since this phenomenon was observed in different cell lines (HeLa and 293 T), cell type specificity can be excluded. Our parallel experiments confirmed that WIP1 is the primary phosphatase responsible for dephosphorylating γH2AX. Considering that the efficient dephosphorylation of γH2AX plays a crucial role in signal transduction and facilitation of DSB repair, these results highlight the critical role of WIP1 in the DDR.

WIP1 was initially identified as a p53-induced phosphatase [46]. As a known oncogene, WIP1 is amplified or overexpressed in numerous cancers, including breast, ovarian, gastrointestinal, leukemia, and brain cancers [38]. The oncogenic activity of WIP1 involves suppressing p53 through direct dephosphorylation of p53 at Ser15 and MDM2, which can lead to the degradation of p53 protein [47, 48]. Recent studies have shown that targeting WIP1 can significantly inhibit cancer cell growth [49–51] or increase the sensitivity of tumor cells to cytotoxic therapies [52]. We demonstrate that AMPK phosphorylates WIP1, which enhances its stability and binding affinity to γH2AX. Interestingly, total and phosphorylated p53 at Ser15 remain unaffected by AMPK activation (Fig. 1B), suggesting that phosphorylation of WIP1 at T25 may promote its specific binding to γH2AX without significantly impacting its interaction with other substrates such as p53.

Under stress conditions, some activated AMPK translocates to the nucleus to regulate different signaling pathways [53]. WIP1 can increase the accumulation of activated AMPK in the nucleus by several mechanisms. First, WIP1 interacts with AMPK, physically preventing the interaction between AMPK and the dephosphatase PP2A, thereby protecting the Thr172 phosphorylation of AMPK from dephosphorylation. Second, AMPK moves between the cytoplasm and the nucleus along a pathway that depends on the RanGTPase system [54]. The redundant nuclear WIP1 interacts with Ran GTPase activating protein 1 (RANGAP1) [55], which may inhibit the transport of activated AMPK from the nucleus to the cytoplasm. However, we could not exclude the possibility that AMPK translocation between the nucleus and cytoplasm also occurs via other, currently unknown mechanisms.

The AMPK-WIP1 axis represents a critical double-edged sword in cellular fate, with fundamentally opposing outcomes in normal versus cancerous contexts. While AMPK activation is widely recognized as a tumor suppressor in normal cells, its role in established tumors remains complex and context-dependent [15, 56–58]. Crucially, our findings highlight that AMPK’s activation of the phosphatase WIP1 is central to this dichotomy. In normal cells, AMPK-induced WIP1 activity supports genomic stability by facilitating DNA repair and serving as a strong tumor-suppressive mechanism. However, cancer cells perversely utilize this same AMPK-WIP1 axis for their survival. Once tumorigenesis occurs, AMPK activation enables cancer cells to hijack WIP1 activity, thereby evading cell death induced by genotoxic stresses, such as ionizing radiation. This corrupts a protective mechanism into a driver of radioresistance and tumor progression. Therefore, the AMPK-WIP1 axis shifts from guardian in normal tissue to accomplice in cancer, indicating that therapeutic AMPK modulation must be precisely targeted to the cellular context to prevent unintended promotion of tumor resilience.

## Supplementary information

Table 1

Table 2

Figure S1

Figure S2

Figure S3

Figure S4

Figure S5

Figure S6

Figure S7

Figure S8

Figure S9

Figure S10

Checklist

original blots

## Data Availability

All data are available upon request from the corresponding author.
