# Supplementary material for: AMPK phosphorylates WIP1 to promote DNA repair and radioresistance in cancer cells
Source: Cell Death Dis. 2025 Nov 28;16(1):864. doi: 10.1038/s41419-025-08141-7 (PMC12663271; doi:10.1038/s41419-025-08141-7)
Supplement: Supplementary file 1 — Table 1 [file 41419_2025_8141_MOESM1_ESM.docx]

| App. | Species | gene | Forward primer (5’-3’) | Reverse primer (5’-3’) |
| --- | --- | --- | --- | --- |
| RT | human | *ACTIN* | CCTTCAACACCCCAGCCATGTACG | GGCACAGTGTGGGTGACCCCGTC |
| RT | human | *WIP1* | GGGAGTGATGGACTTTGGAATA | ATGCTCACCCATCAGGTATTT |
| RT | human | *AMPKα2* | CTCTAGTCCTCCATCTGGTTCT | GTCTGCTATAAGAGGTGGCATC |
| RT | human | *PPP2R5A* | GCCACTGAAAGCAGAACATAAA | GCTAGCTGAGCATGAAACAAAG |
| RT | human | *PPP2R5B* | CCCTGAATTTGACCCTGAAGA | CTCTCCAAGAAACGCAGGAA |
| RT | human | *PPP2R5C* | CACAGAGCCTATTTACCCAGAAG | GGATTGGAGGAAGGTGGTAATG |
| RT | human | *PPP2R5D* | TCCTCATCCGTGTCCTACTT | GCTCTCCTTCTCCAGGAATTG |
| RT | human | *PPP2R5E* | AACAAATCGCCAAGTGTGTATC | GATGGGAAGGATGACGTTAGAG |
| RT | human | *PPP1R12A* | GCCAAATAGTCTTGTAGGCATAAC | CTTCTCCTTCTTTCTCCTCTTCTC |
| RT | human | *PPP2AC* | GGTTACACCTTTGGGCAAGATA | GGTCATGGCACCAGTTATATCC |
| RT | human | *PP4C* | CTCAAAGAGCTGTTCAGAGTAGG | GGAACGTTTCGACGCTATAGAA |
| RT | human | *PPP1CA* | GCTGCTGGCCTATAAGATCAA | GTCTCTTGCACTCATCGTAGAA |
| siRNA | human | *PP4C-NC* | UUCUCCGAACGUGUCACGUTT | ACGUGACACGUUCGGAGAATT |
| siRNA | human | *PP4C-1* | GGCCAGAGAGAUCUUGGUAUU | UACCAAGAUCUCUCUGGCCUU |
| siRNA | human | *PP4C-2* | GCUACUGCACUGAGAUCUUTT | AAGAUCUCAGUGCAGUAGCTT |
| shRNA  shRNA  gRNA  NGS | human  human  human  human | *WIP1-1*  *WIP1-2*  *WIP1*  *WIP1* | CCGGCCTCAGAAGCACAAGTATATTCTCGAG-  AATATACTTGTGCTTCTGAGGTTTTTG  CCGGCGAGAGAATGTCCAAGGTGTACTCGAG-  TACACCTTGGACATTCTCTCGTTTTTG  CACCGGGGCGGGAGGAAGTACATGG  TCGTCGGCAGCGTCAGATGTGTATAAGAGACAG-  **TTGGCCGGCGAGCGCCTAGTGT**^1^ | AATTCAAAAACCTCAGAAGCACAAGTATATTCT-  CGAGAATATACTTGTGCTTCTGAGG  AATTCAAAAACGAGAGAATGTCCAAGGTGTACT-  CGAGTACACCTTGGACATTCTCTCG  AAACCCATGTACTTCCTCCCGCCCC  GTCTCGTGGGCTCGGAGATGTGTATAAGAGACAG-  **GCTGGGCCTTTCCCCGAGACTT**^1^ |

^1^: The adaptor sequences for NGS are highlighted in underline, and the targeting sequences are highlighted in bold.
