# Supplementary figures and images for: AMPK phosphorylates WIP1 to promote DNA repair and radioresistance in cancer cells
Source: Cell Death Dis. 2025 Nov 28;16(1):864. doi: 10.1038/s41419-025-08141-7 (PMC12663271; doi:10.1038/s41419-025-08141-7)

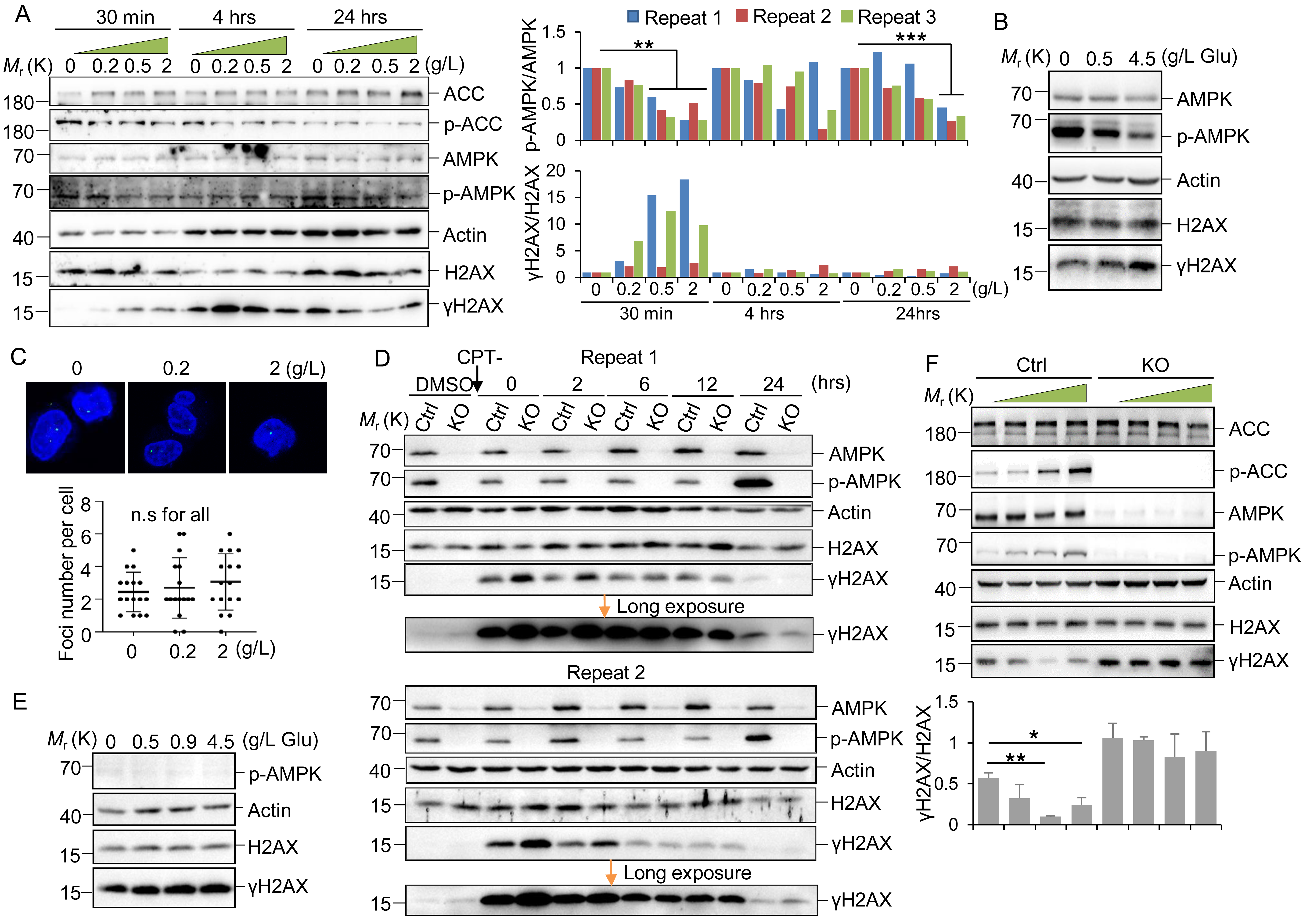

Supplement: Supplementary file 3 — Figure S1 [file 41419_2025_8141_MOESM3_ESM.tif]

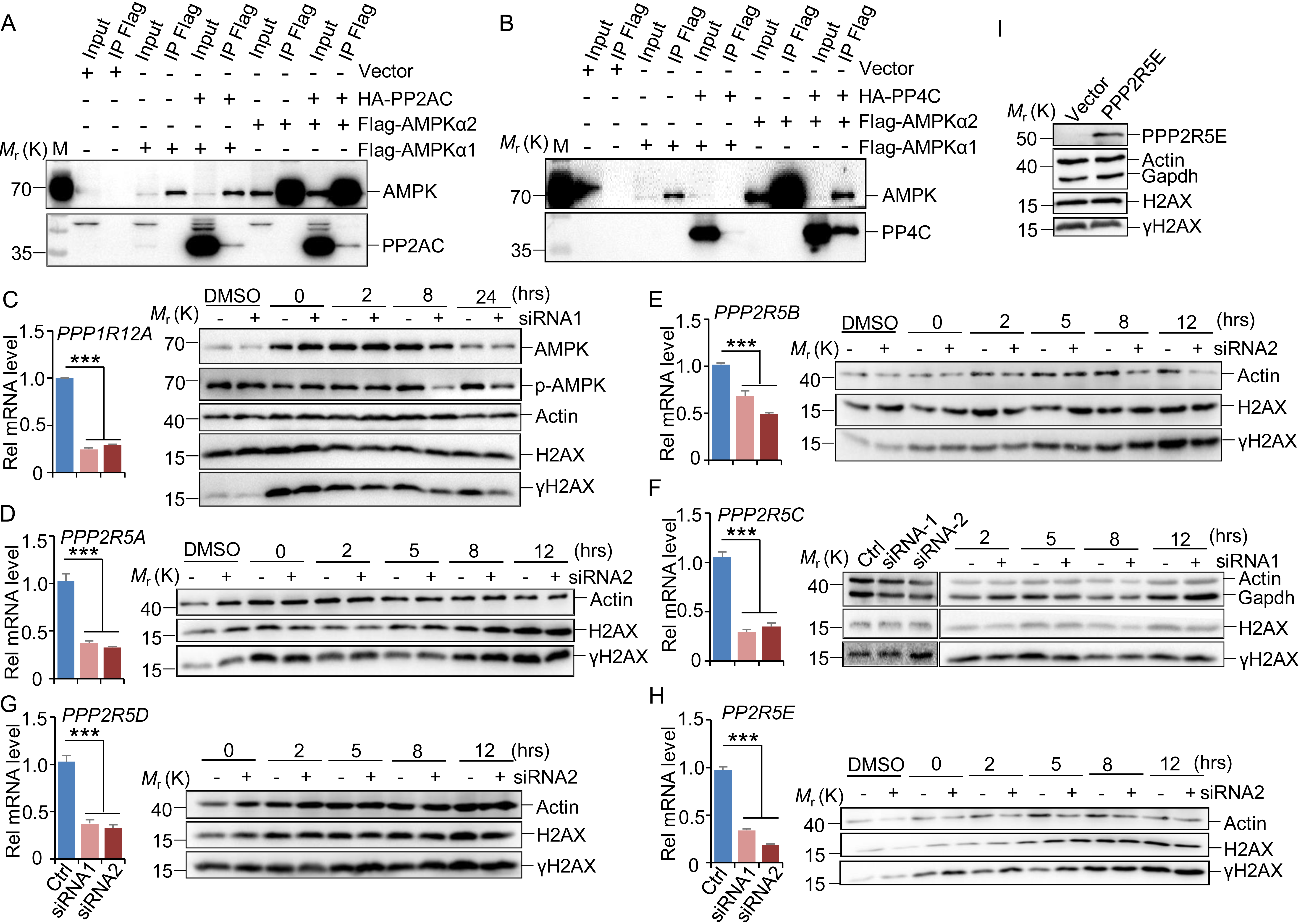

Supplement: Supplementary file 4 — Figure S2 [file 41419_2025_8141_MOESM4_ESM.tif]

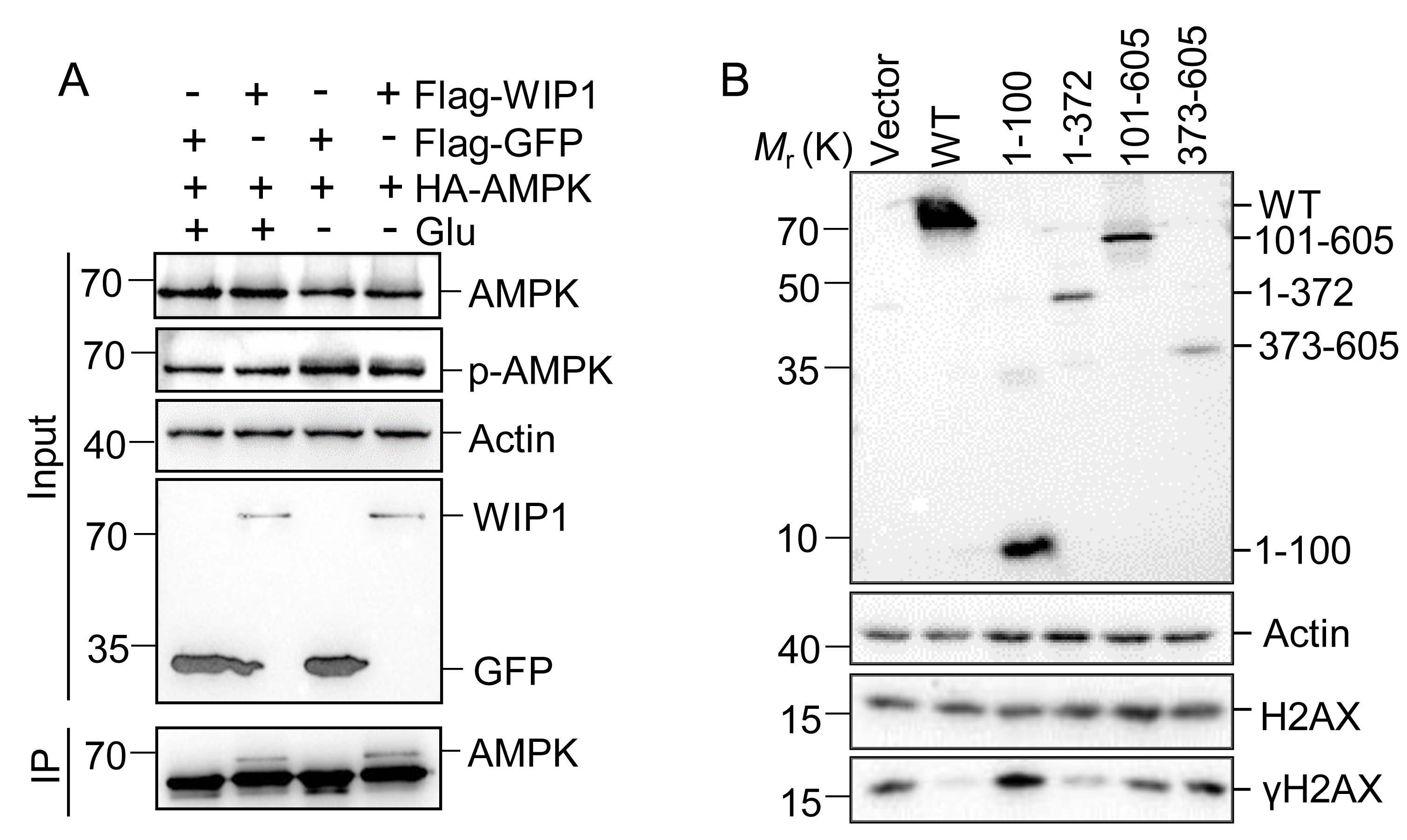

Supplement: Supplementary file 5 — Figure S3 [file 41419_2025_8141_MOESM5_ESM.tif]

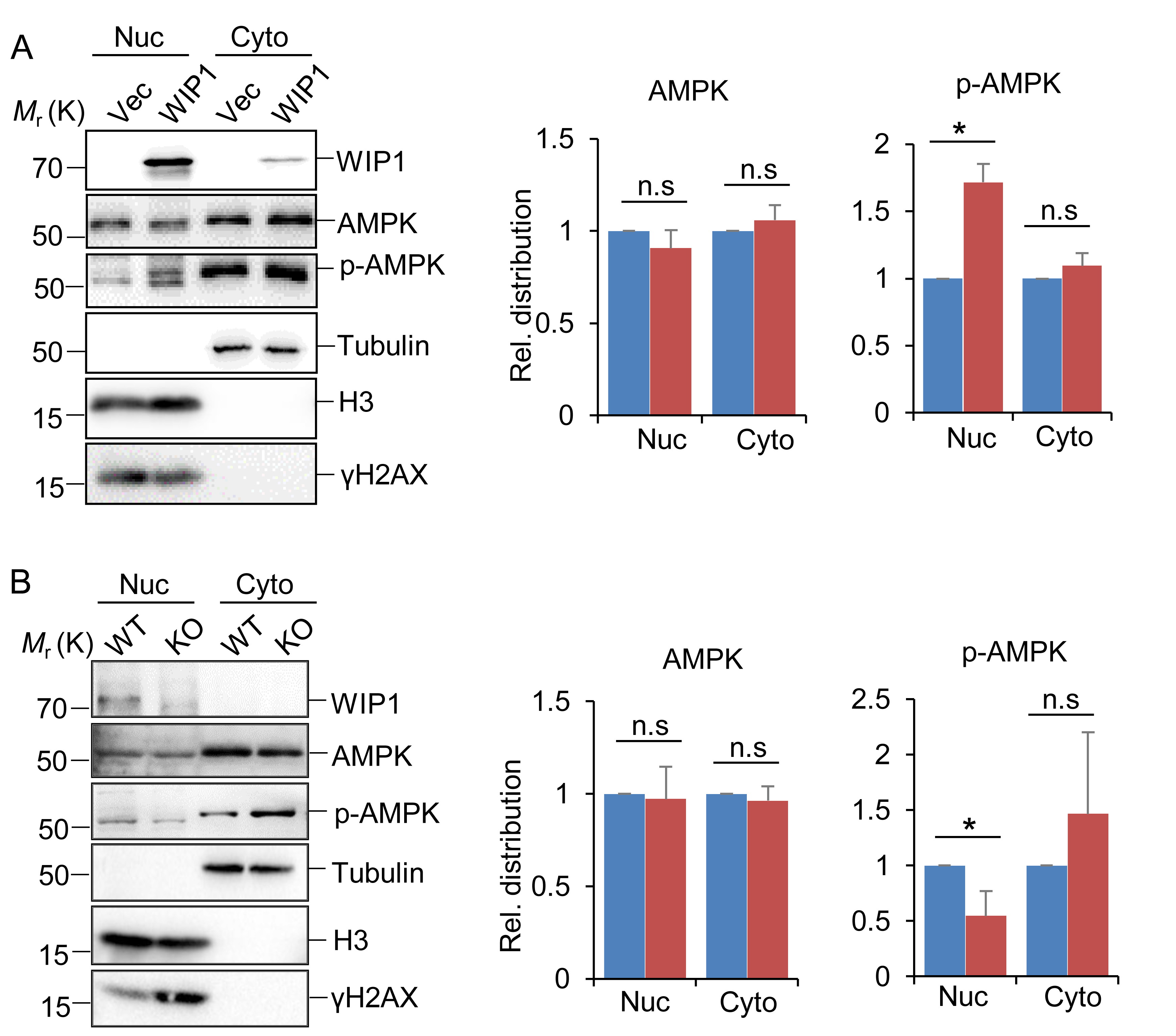

Supplement: Supplementary file 6 — Figure S4 [file 41419_2025_8141_MOESM6_ESM.tif]

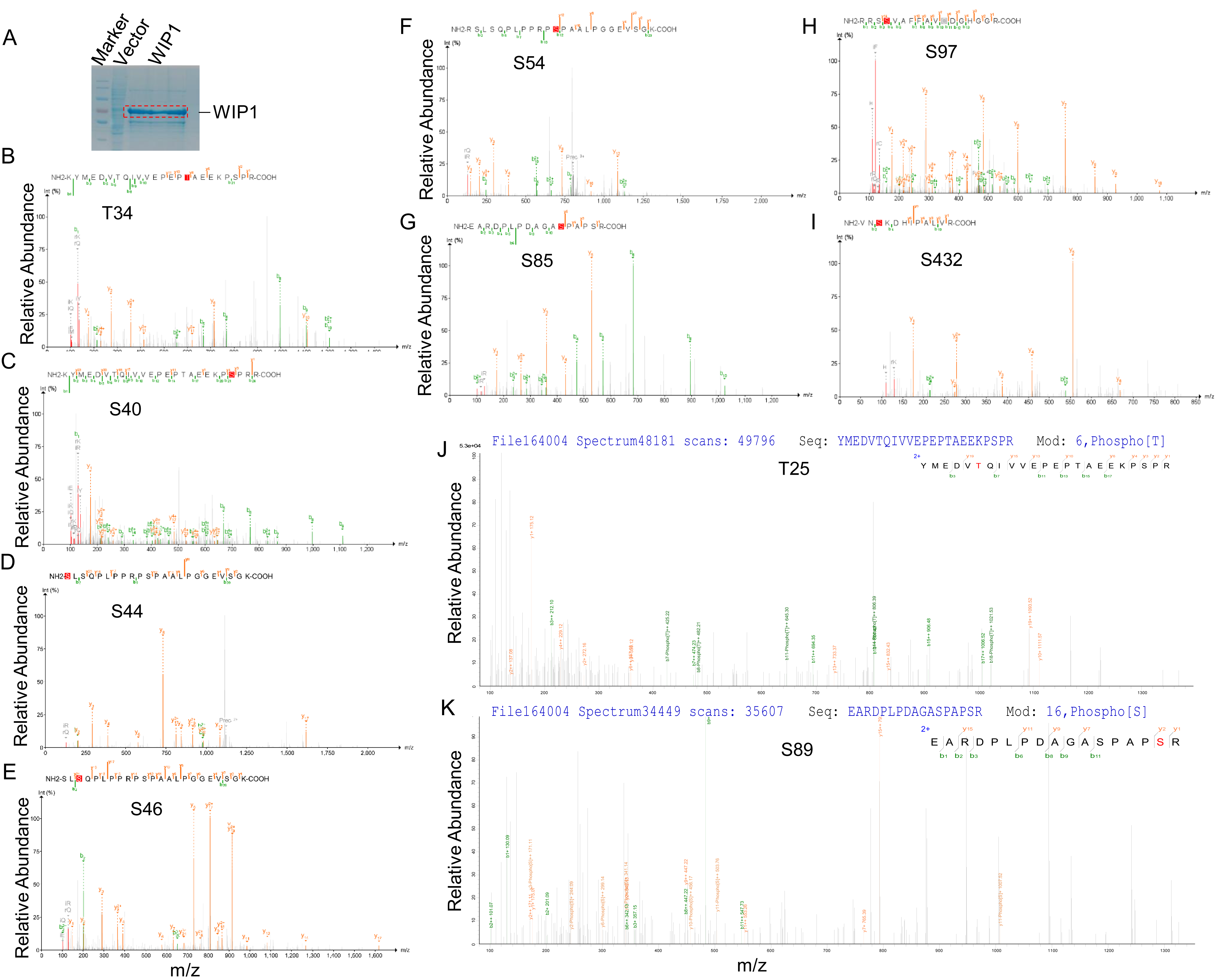

Supplement: Supplementary file 7 — Figure S5 [file 41419_2025_8141_MOESM7_ESM.tif]

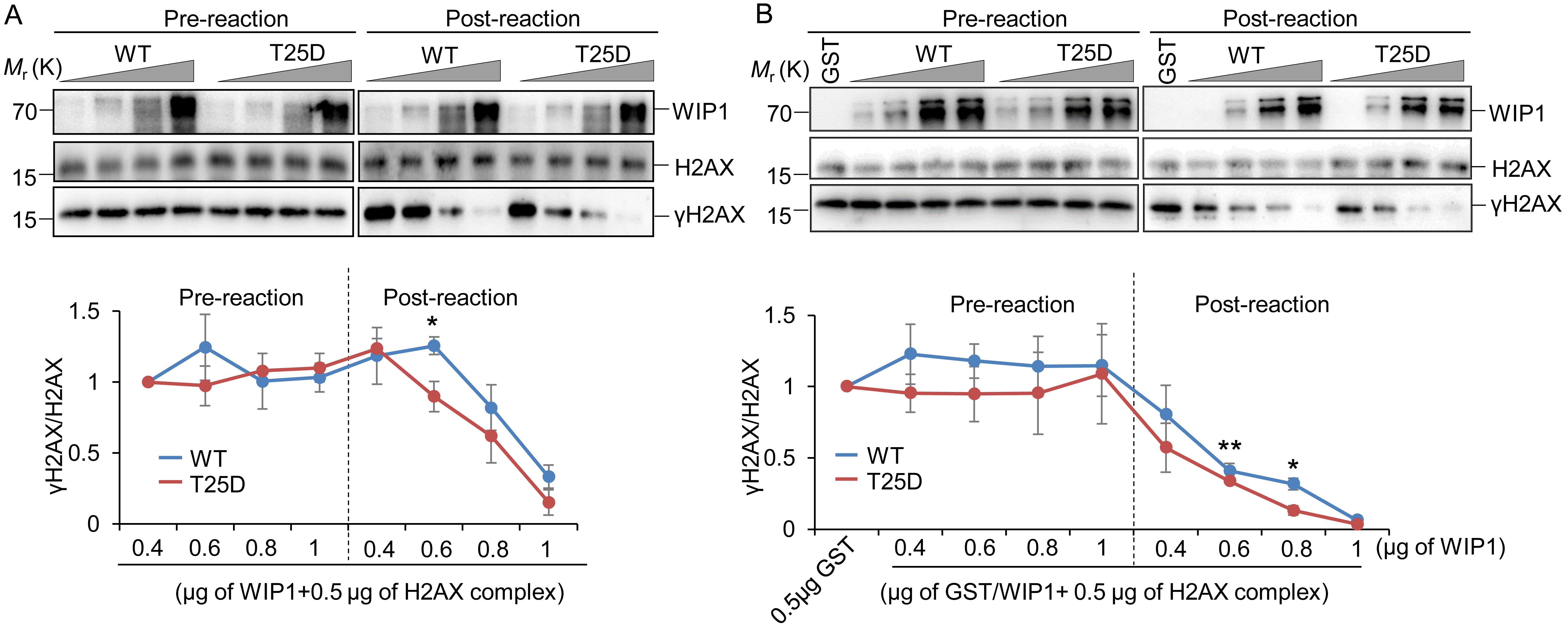

Supplement: Supplementary file 8 — Figure S6 [file 41419_2025_8141_MOESM8_ESM.tif]

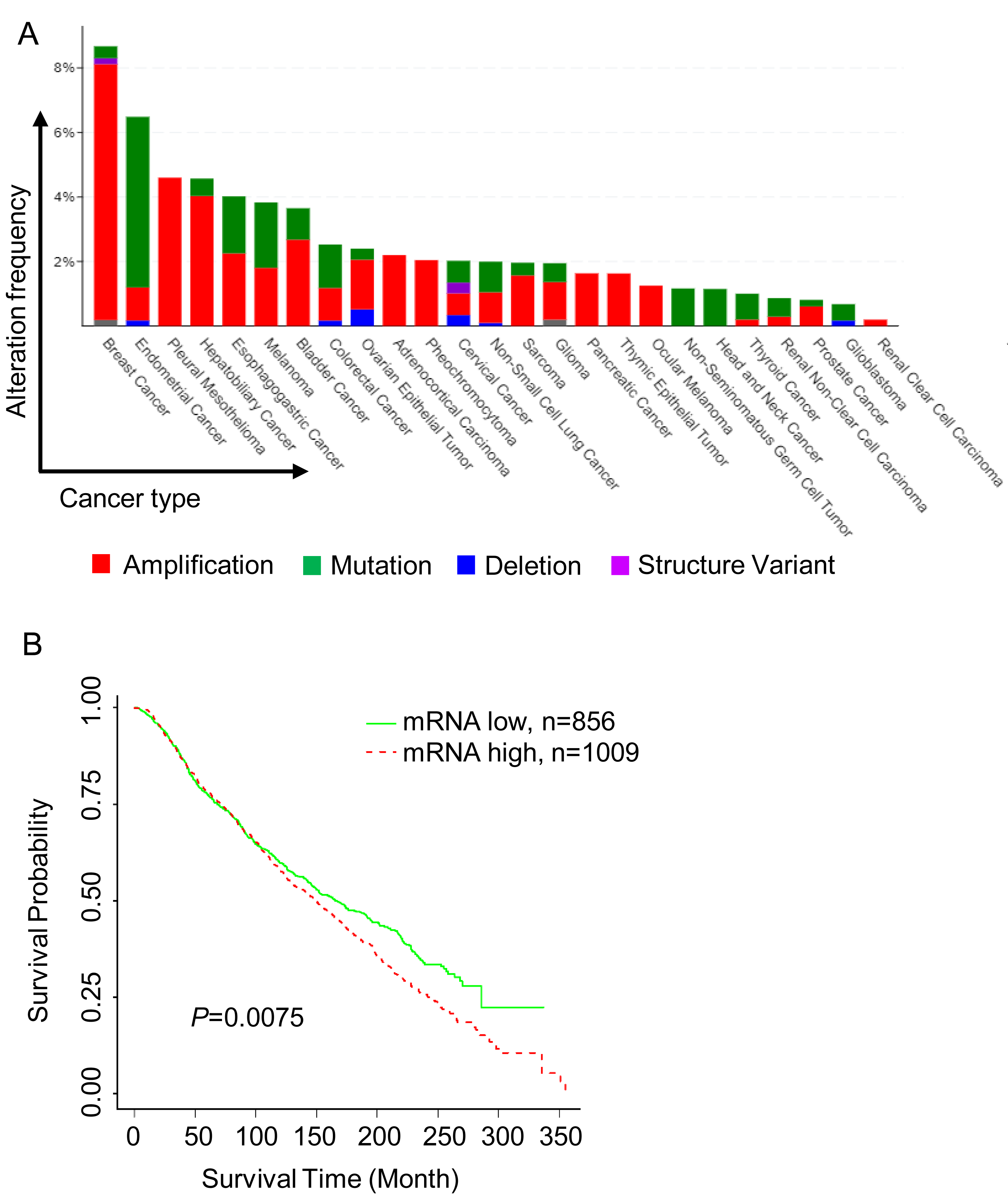

Supplement: Supplementary file 9 — Figure S7 [file 41419_2025_8141_MOESM9_ESM.tif]

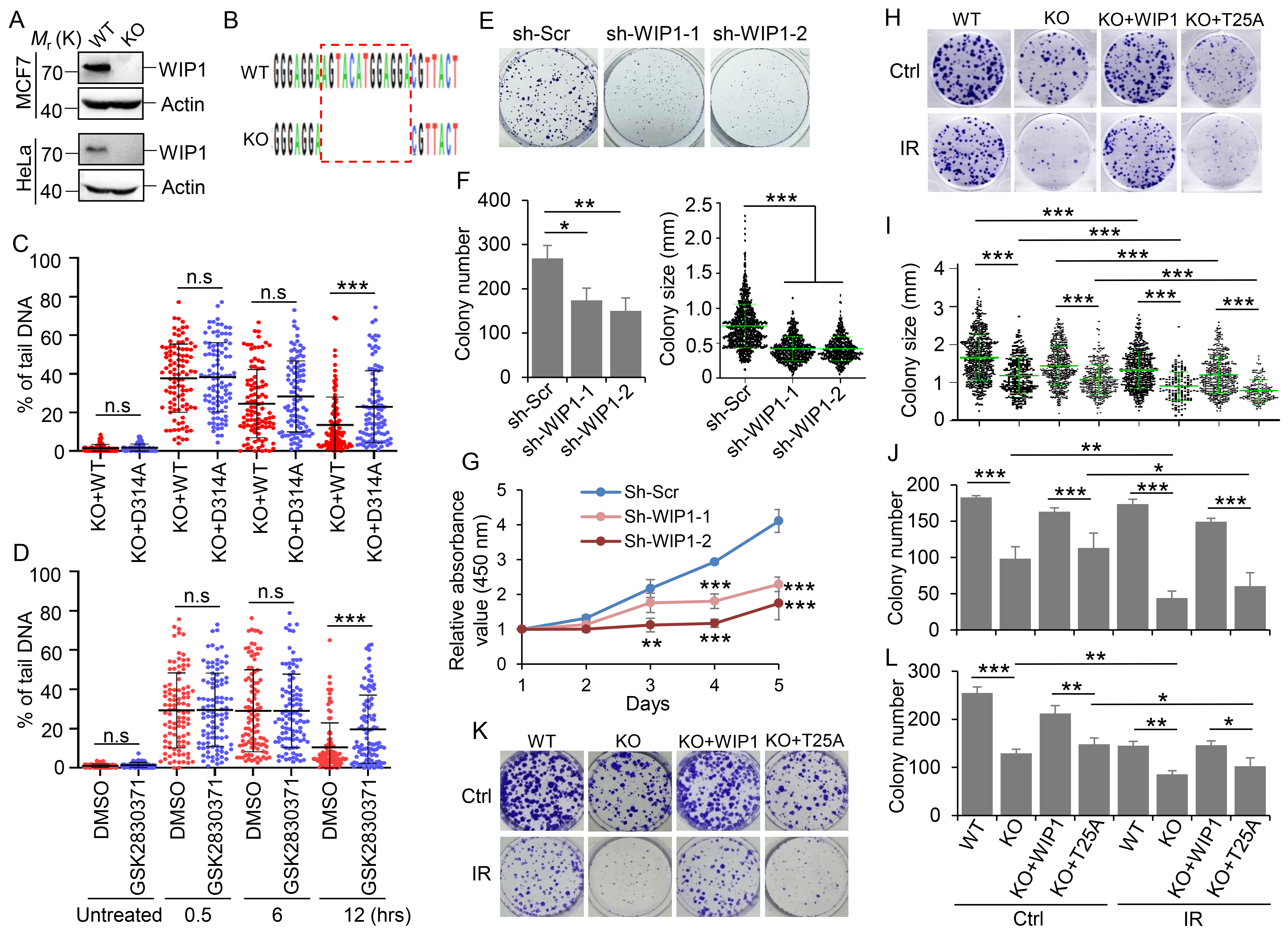

Supplement: Supplementary file 10 — Figure S8 [file 41419_2025_8141_MOESM10_ESM.tif]

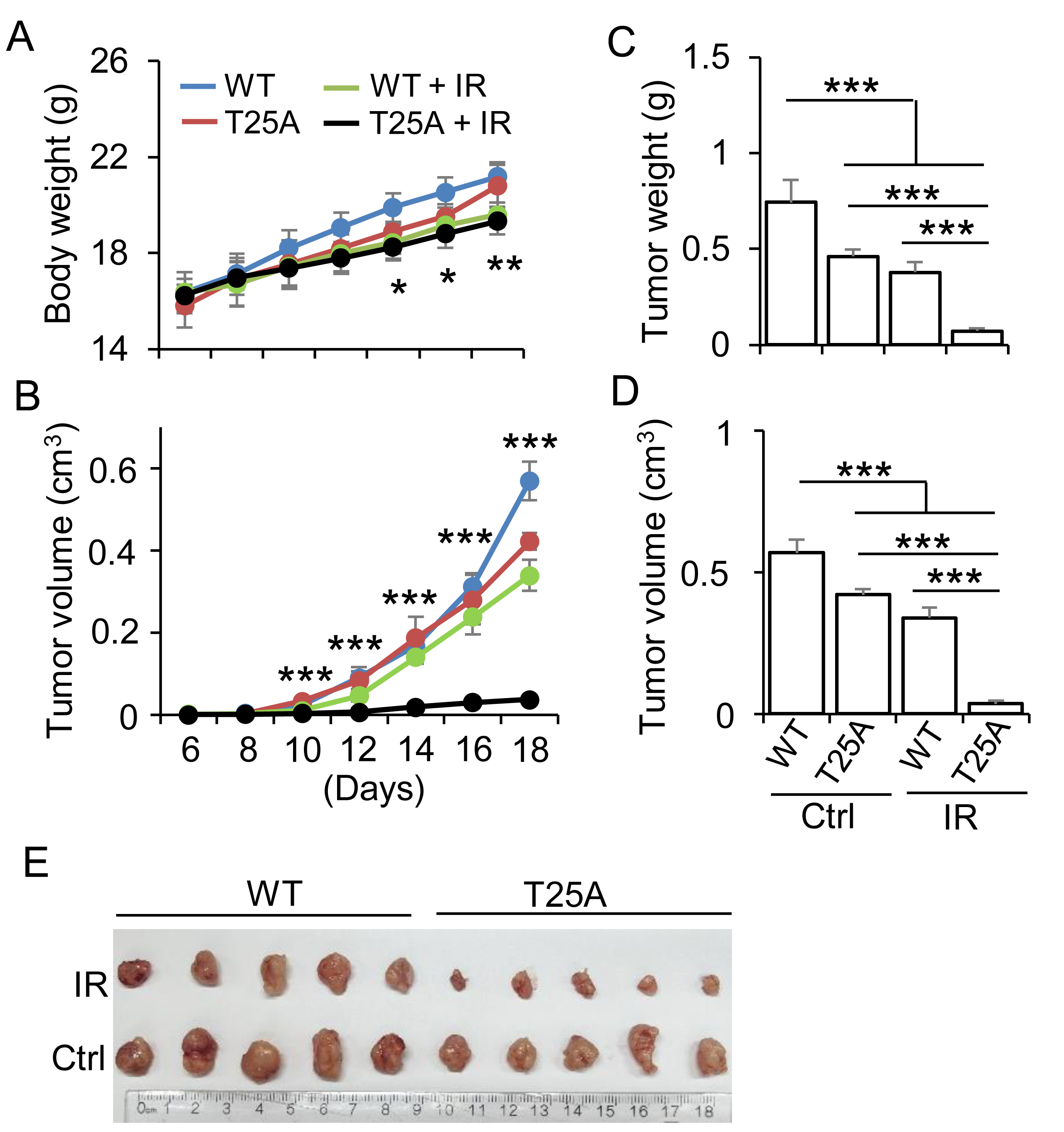

Supplement: Supplementary file 11 — Figure S9 [file 41419_2025_8141_MOESM11_ESM.tif]

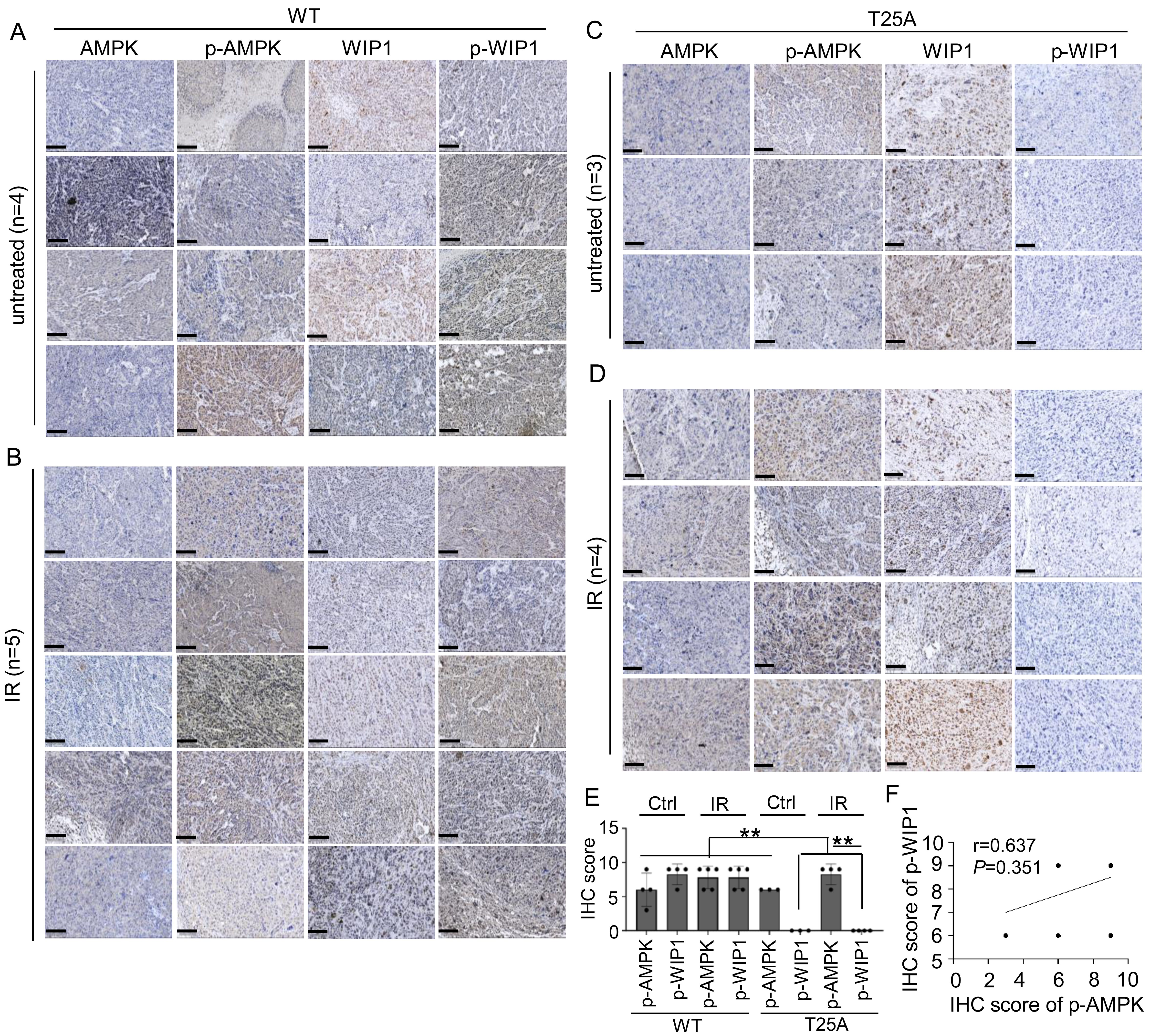

Supplement: Supplementary file 12 — Figure S10 [file 41419_2025_8141_MOESM12_ESM.tif]
