# Supplementary material for: AMPK phosphorylates WIP1 to promote DNA repair and radioresistance in cancer cells
Source: Cell Death Dis. 2025 Nov 28;16(1):864. doi: 10.1038/s41419-025-08141-7 (PMC12663271; doi:10.1038/s41419-025-08141-7)

Figure 1A

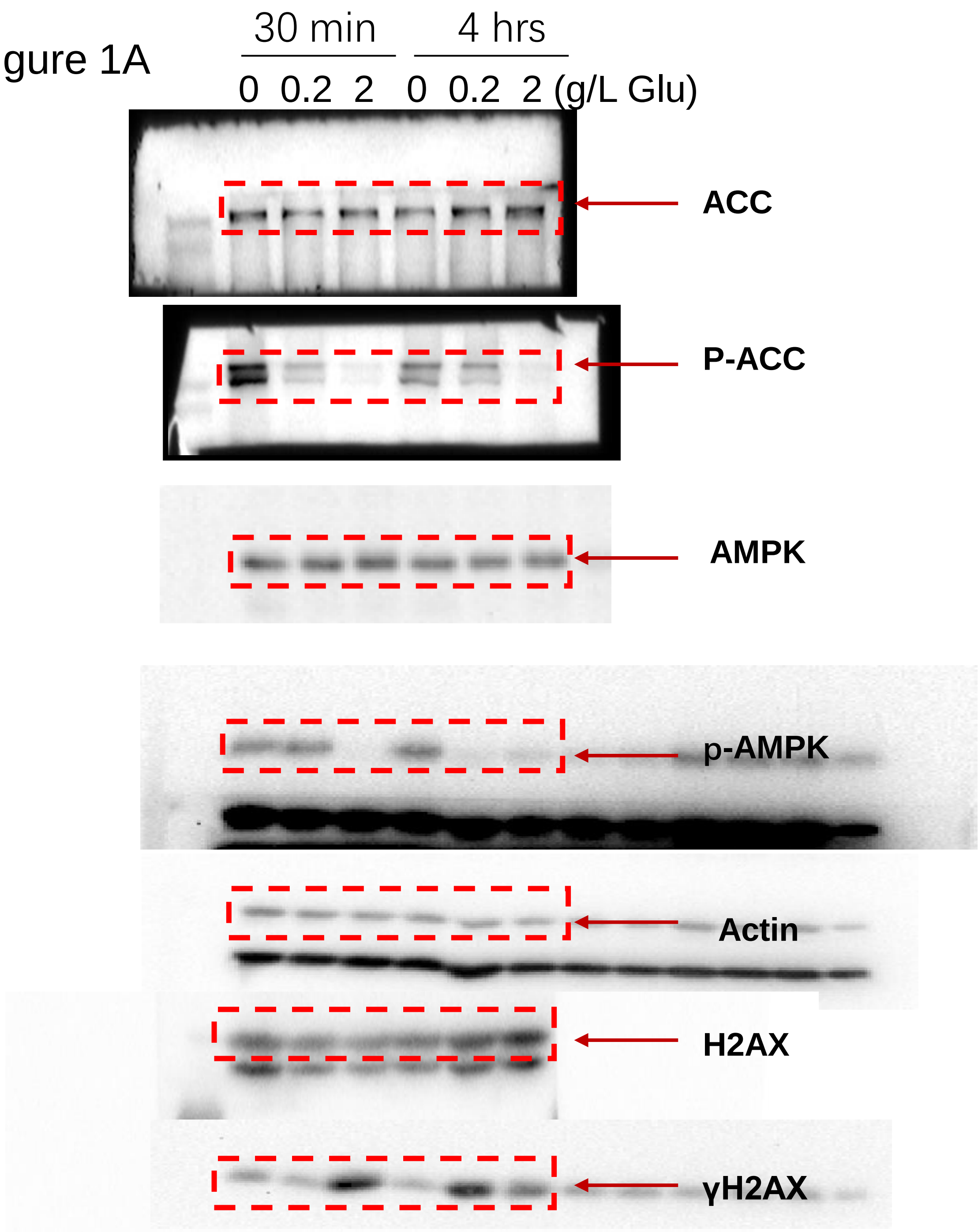
30 min
4 hrs
0 0.2 2 0 0.2 2 (g/L Glu)
ACC
P-ACC
AMPK
p-AMPK
Actin
H2AX
γH2AX

Figure 1B

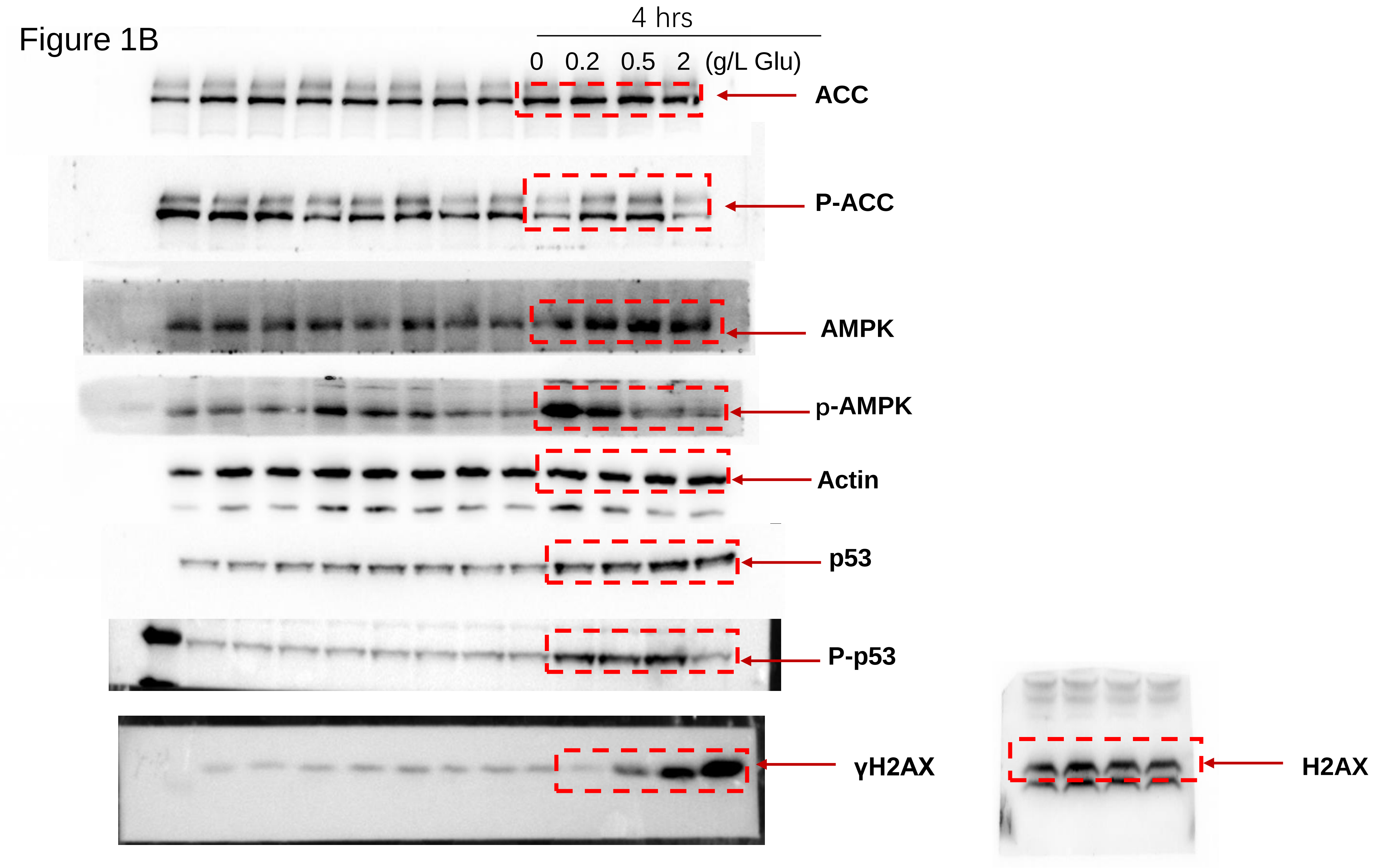
4 hrs
0 0.2 0.5 2 (g/L Glu)
ACC
P-ACC
AMPK
p-AMPK
Actin
p53
P-p53
γH2AX
H2AX

Figure 1C-A769662

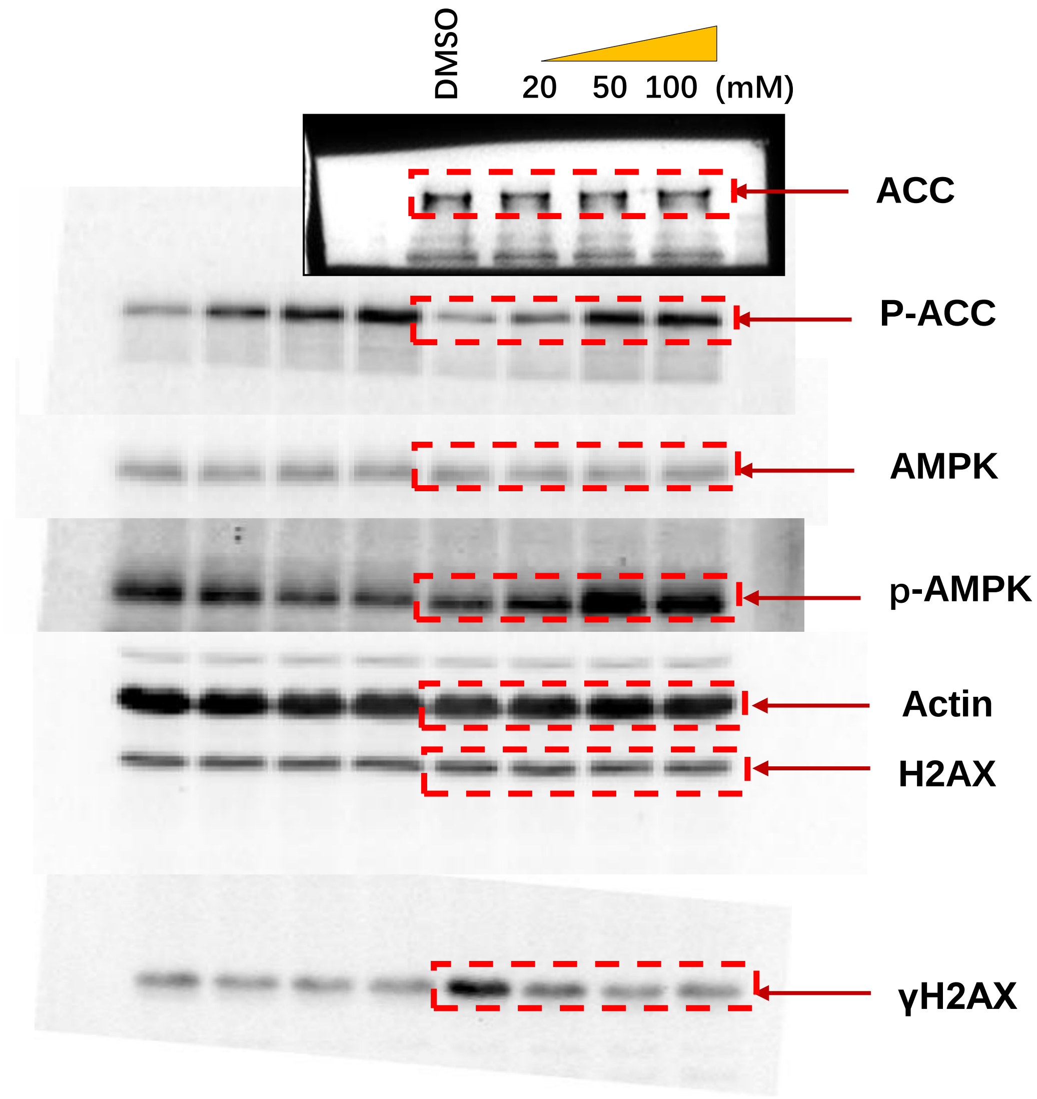

Figure1C-MK-8722

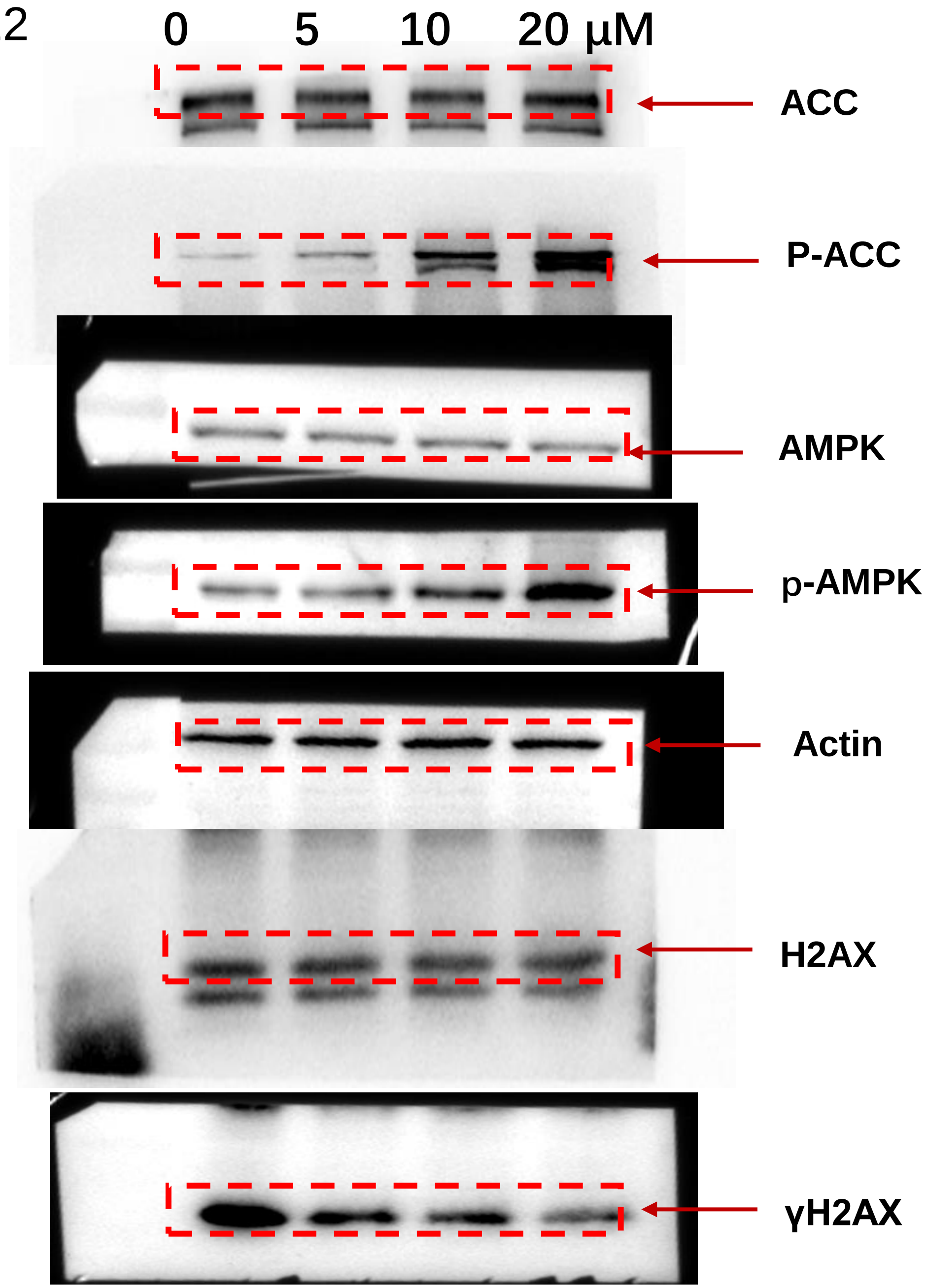

Figure 1E-A769662

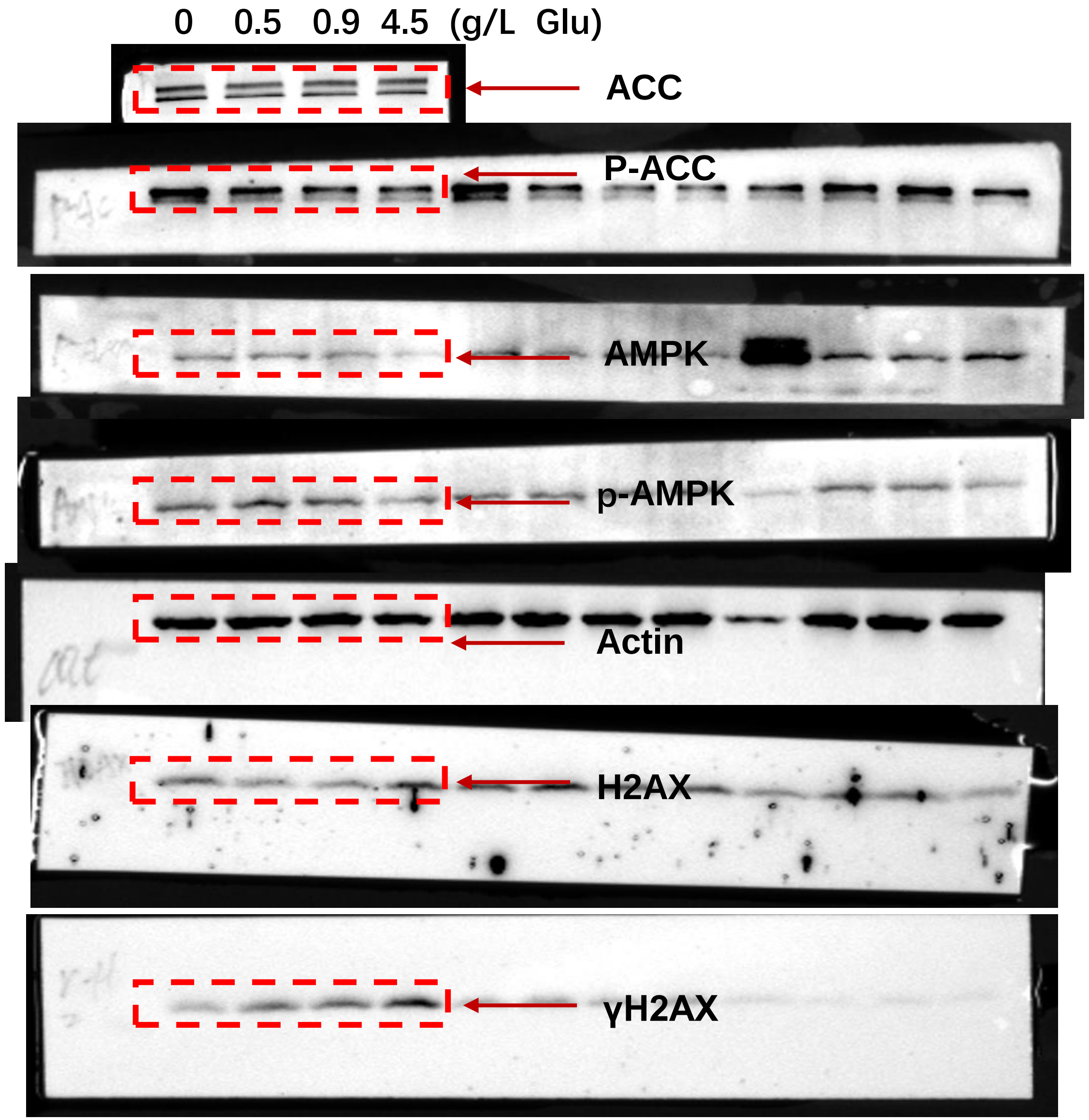

Figure 1G

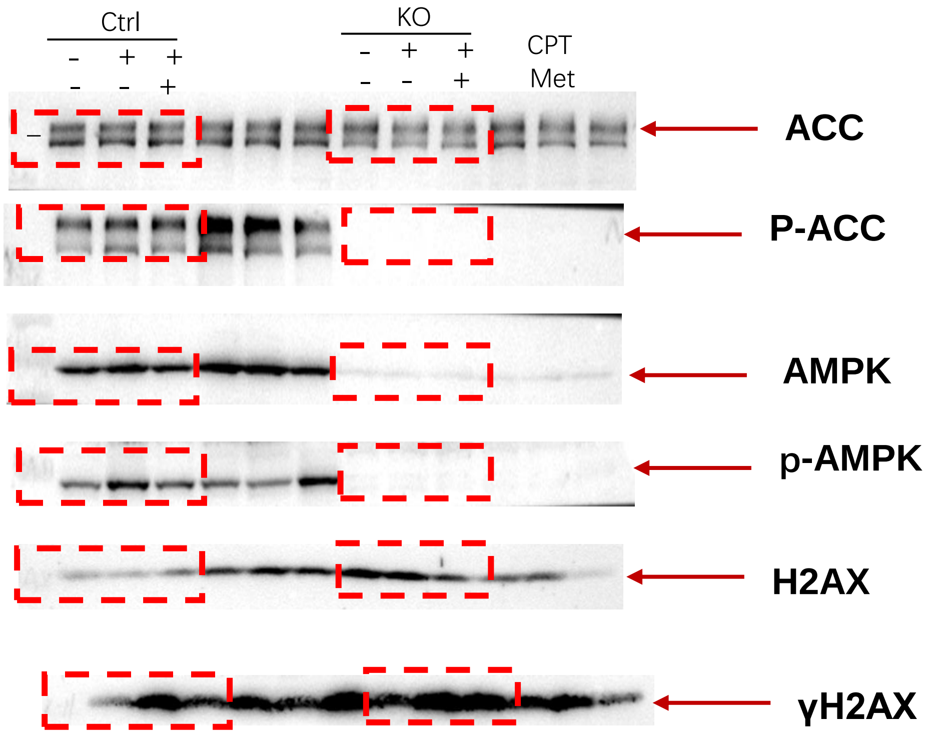

Figure 1H

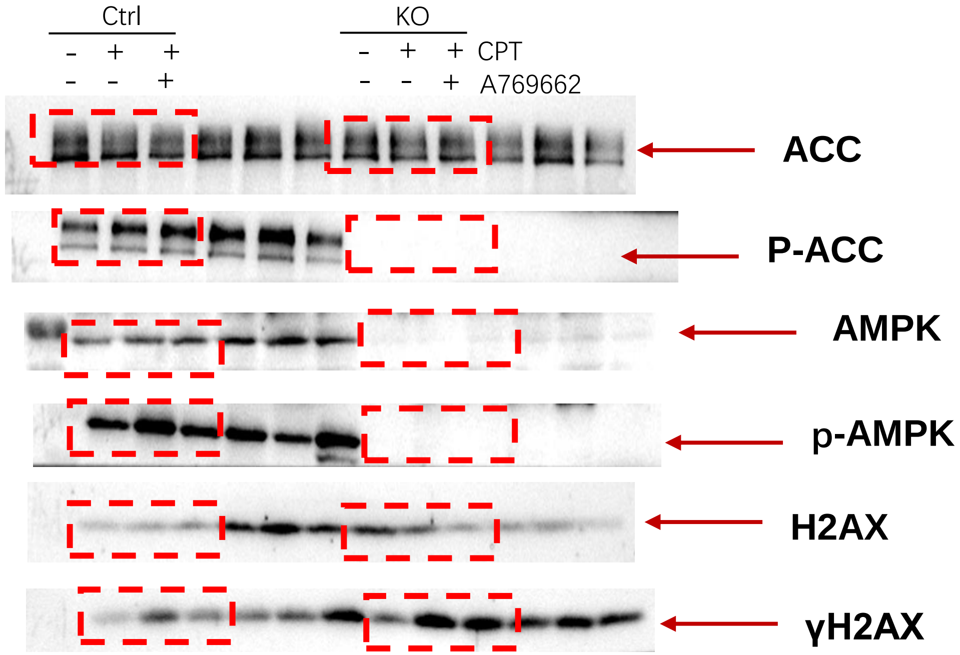

Figure 2A

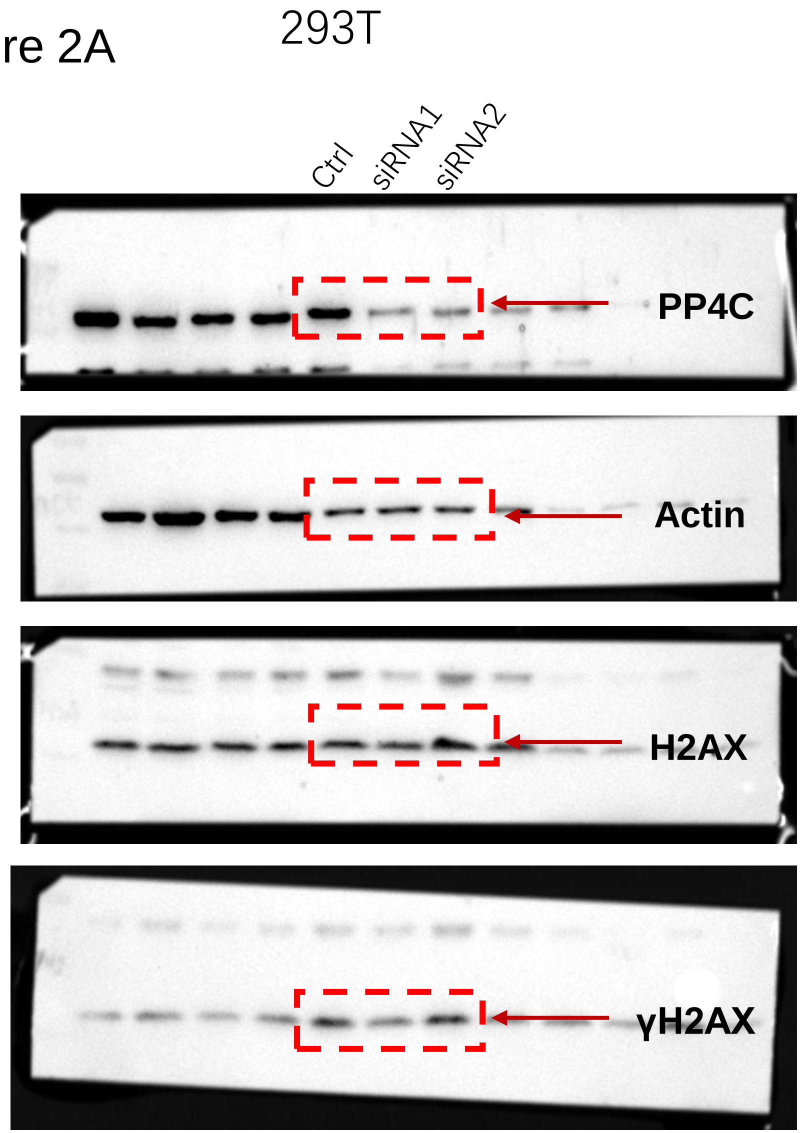
293T
Ctrl
siRNA1
siRNA2
PP4C
Actin
H2AX
γH2AX

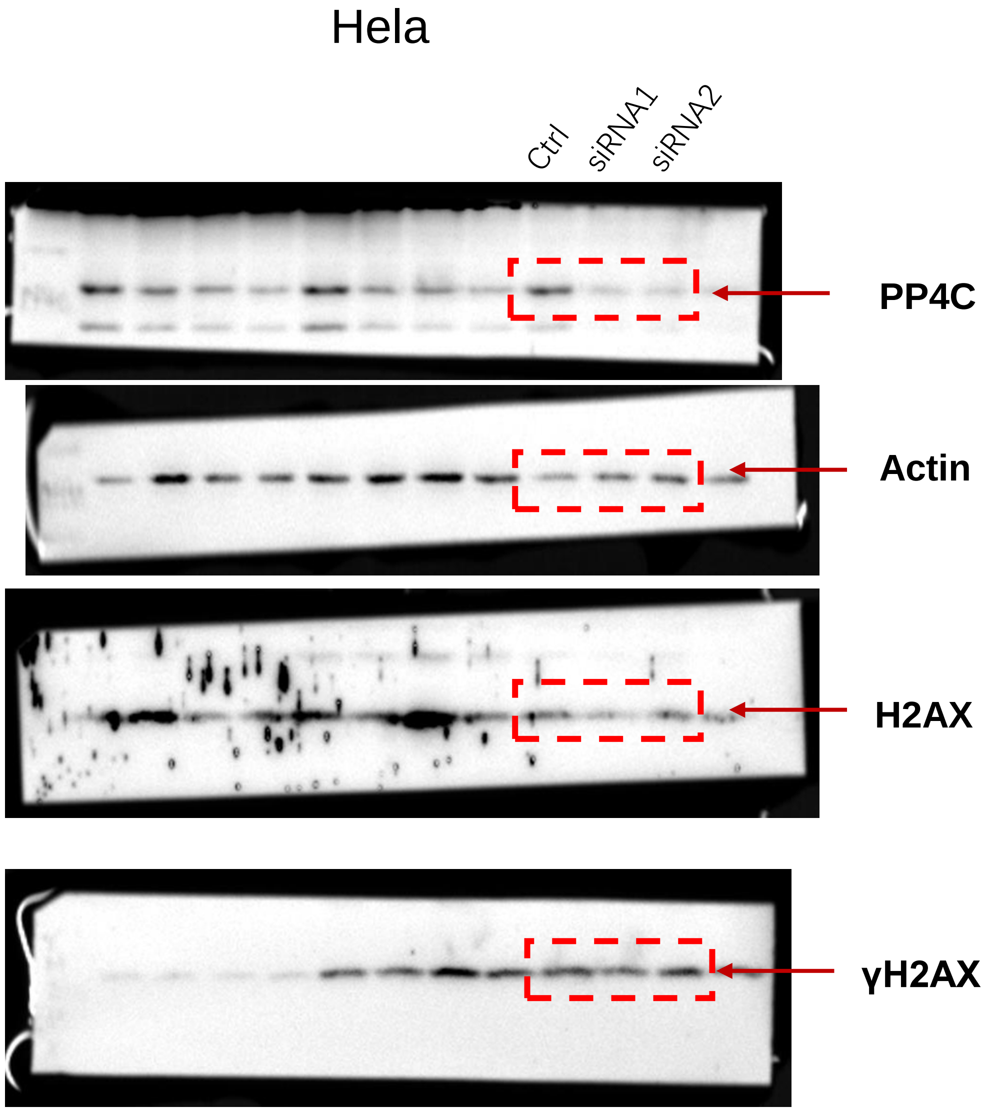
Hela
Ctrl
siRNA1
siRNA2
PP4C
Actin
H2AX
γH2AX

Figure 2B

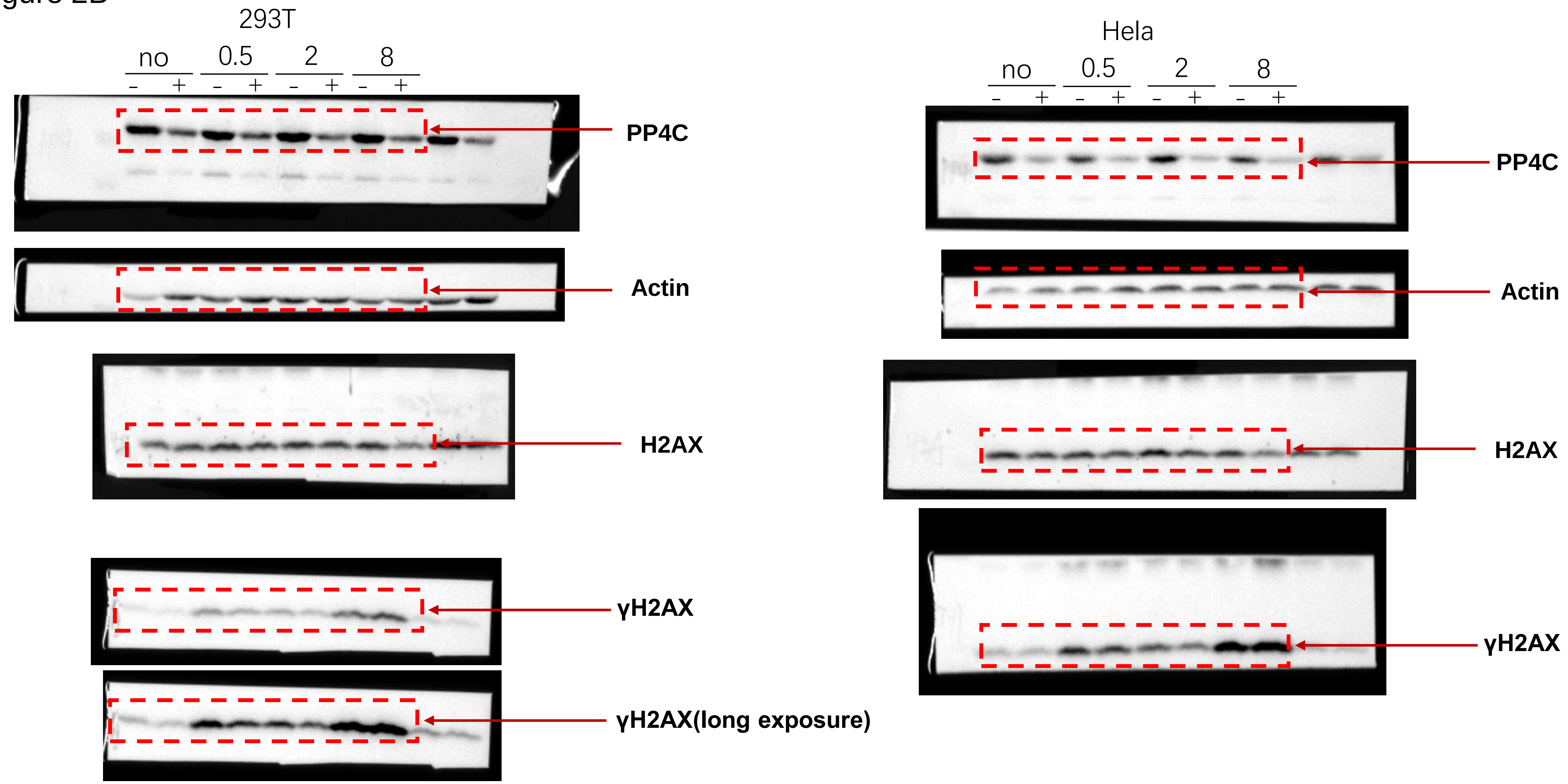

293T
no
0.5
2
8
- + - + - + - +
PP4C
Actin
H2AX
γH2AX
γH2AX(long exposure)
Hela
no
0.5
2
8
- + - + - + - +
PP4C
Actin
H2AX
γH2AX

Figure 2C

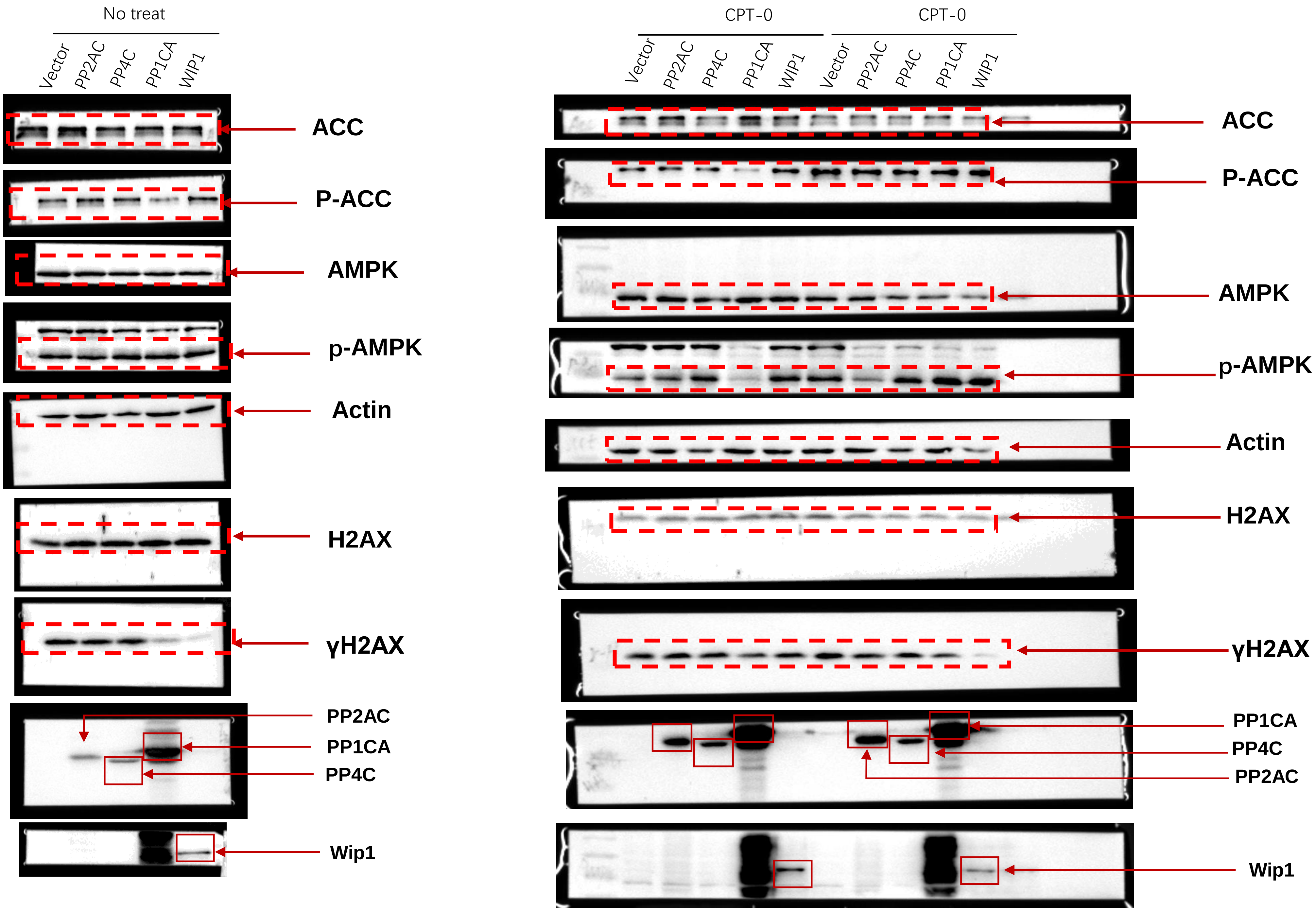
No treat
Vector
PP2AC
PP4C
PP1CA
WIP1
ACC
P-ACC
AMPK
p-AMPK
Actin
H2AX
γH2AX
PP2AC
PP1CA
PP4C
Wip1
CPT-0
CPT-0
Vector
PP2AC
PP4C
PP1CA
WIP1
Vector
PP2AC
PP4C
PP1CA
WIP1
ACC
P-ACC
AMPK
p-AMPK
Actin
H2AX
γH2AX
PP1CA
PP4C
PP2AC
Wip1

Figure 2D

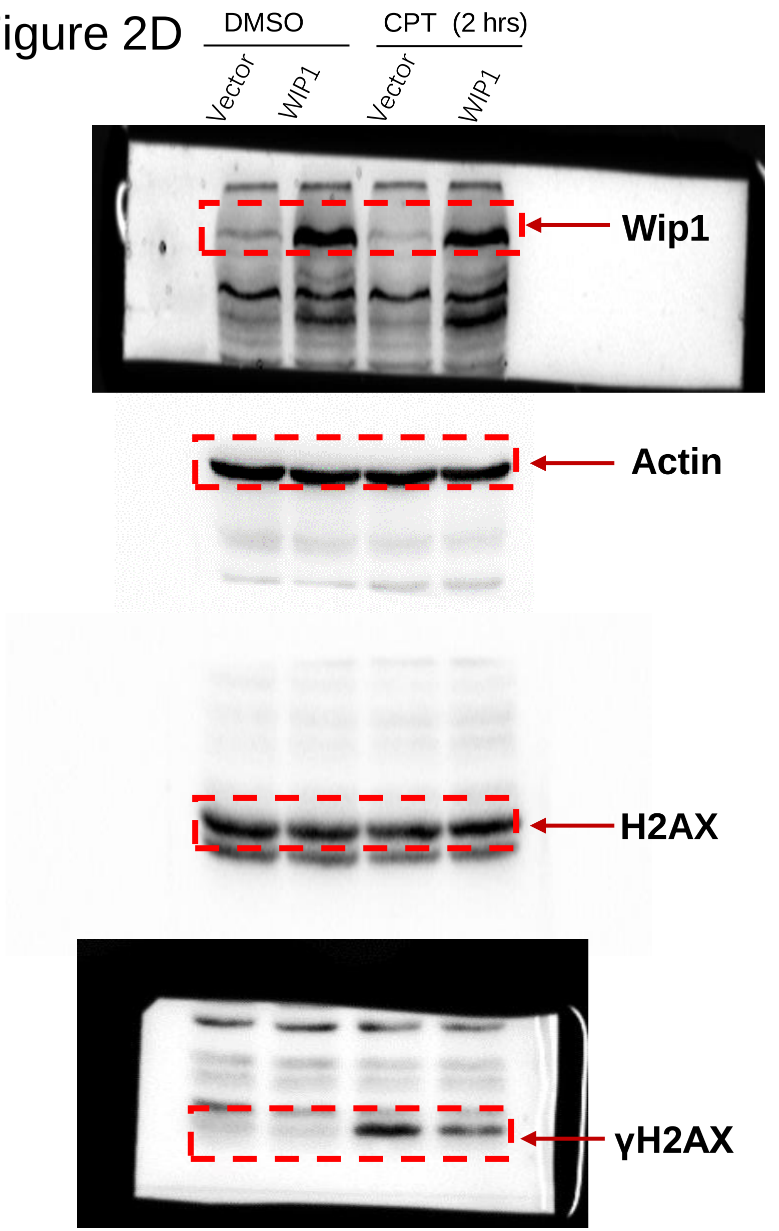

Figure 2G

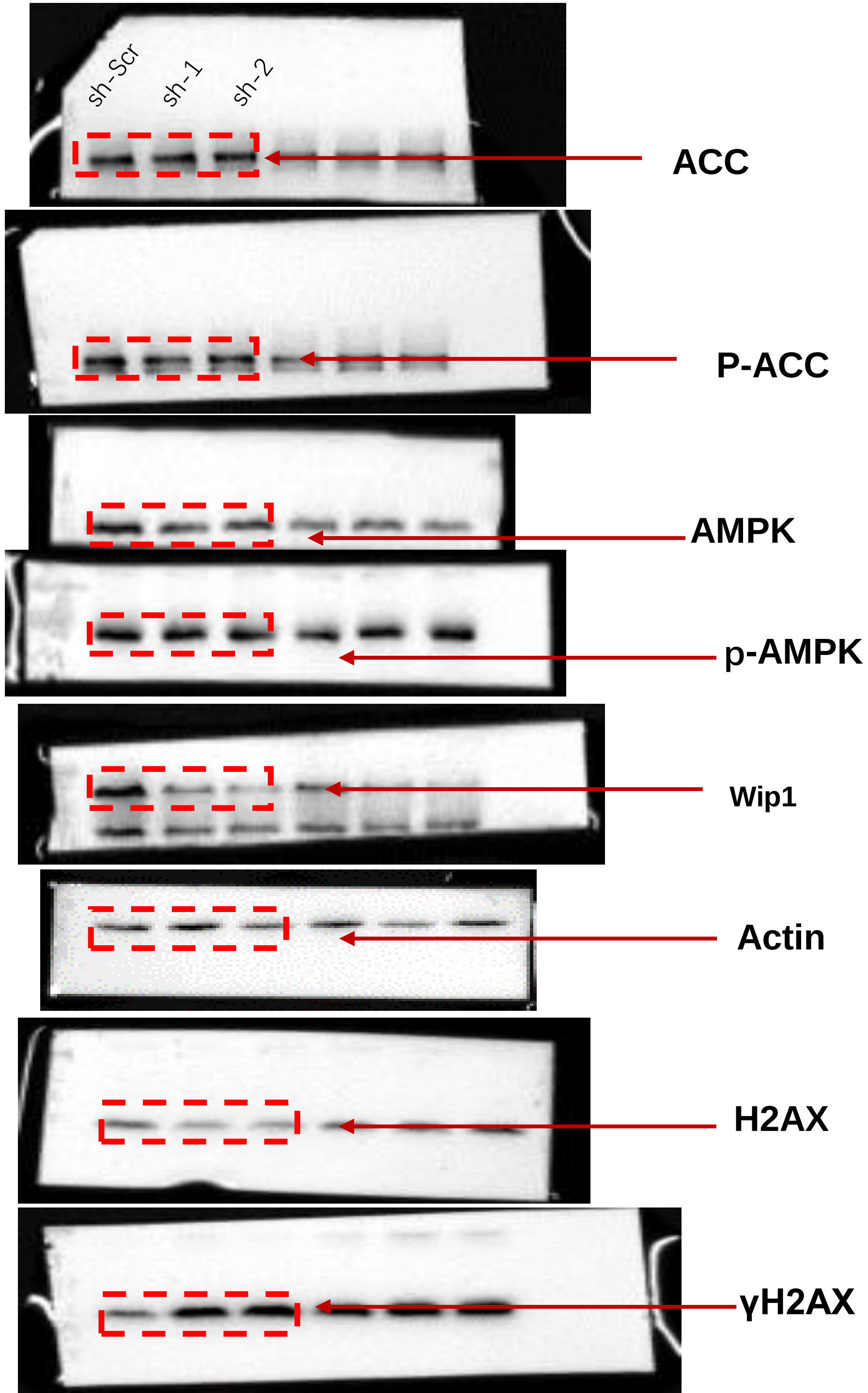

Figure 3A

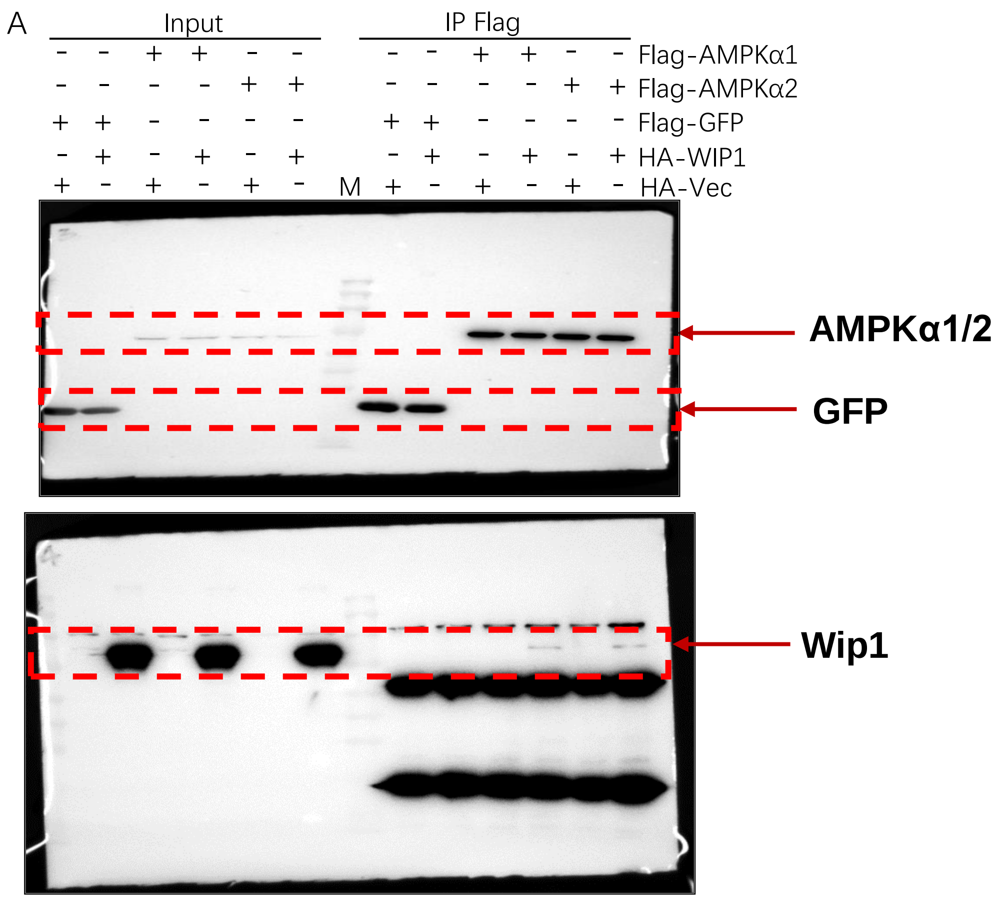

Figure 3B

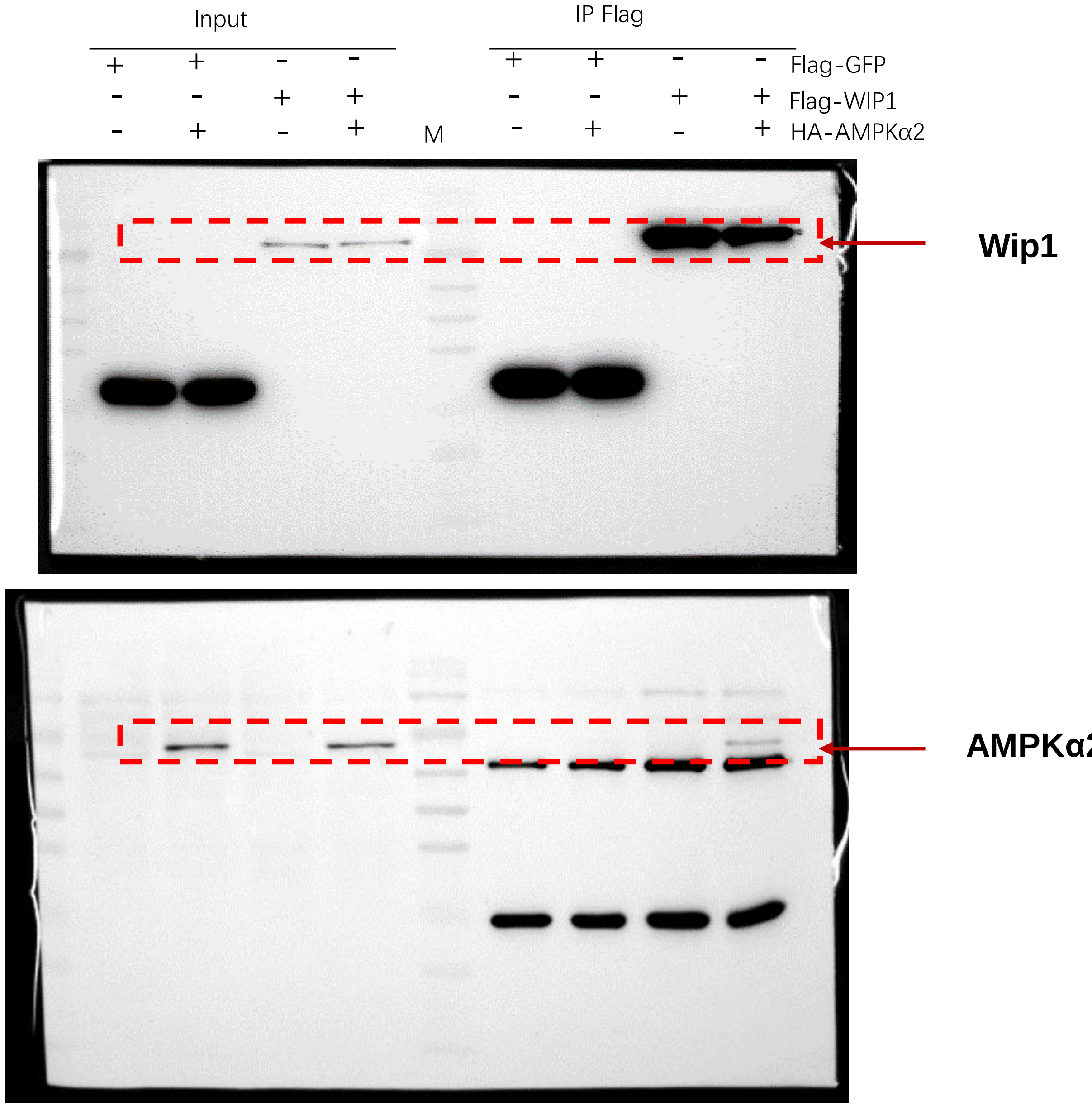

Figure 3C

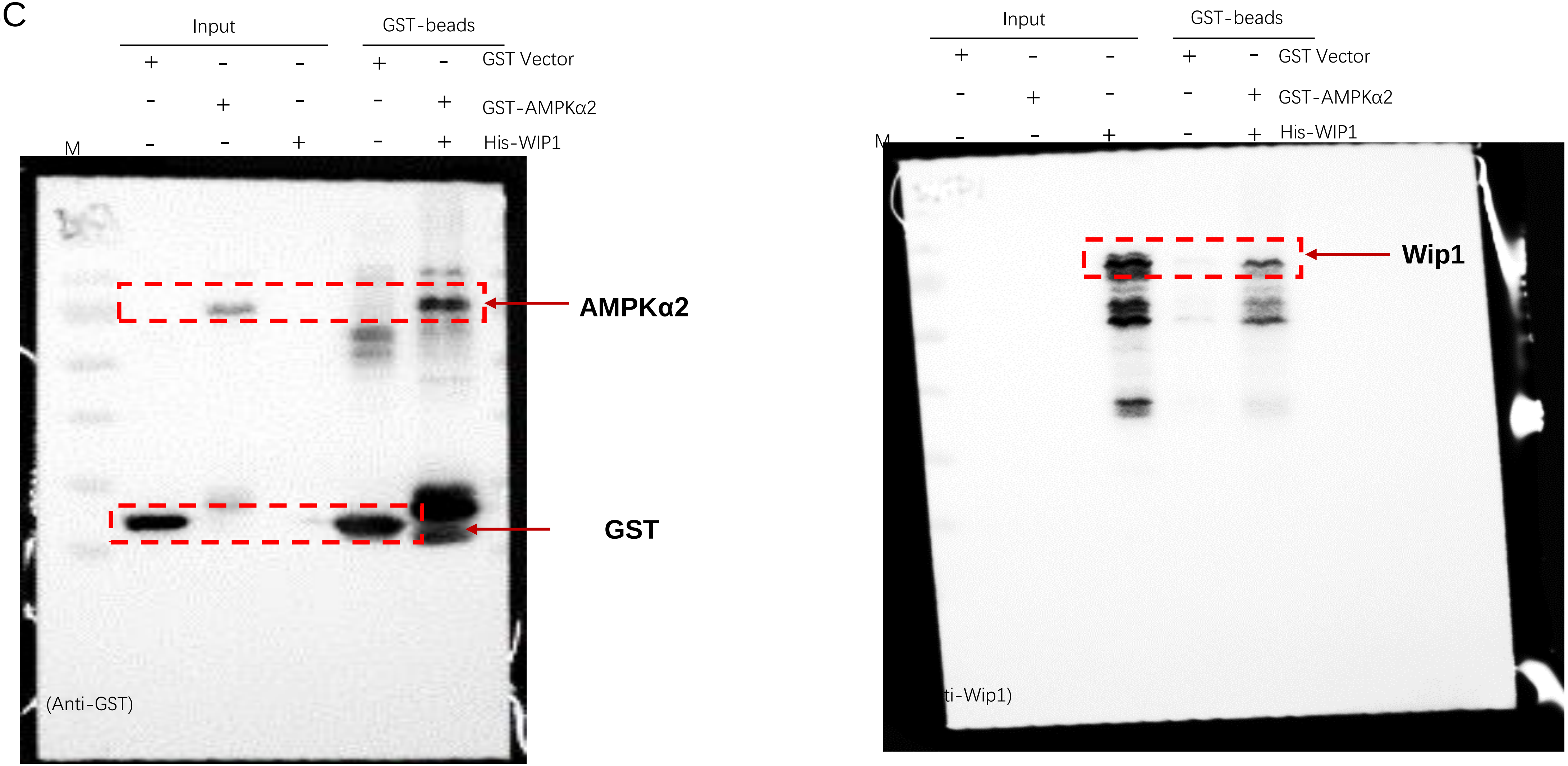
Input
GST-beads
GST Vector
GST-AMPKα2
His-WIP1
M
AMPKα2
GST
(Anti-GST)
Input
GST-beads
GST Vector
GST-AMPKα2
His-WIP1
M
Wip1
ti-Wip1)

Figure 3E

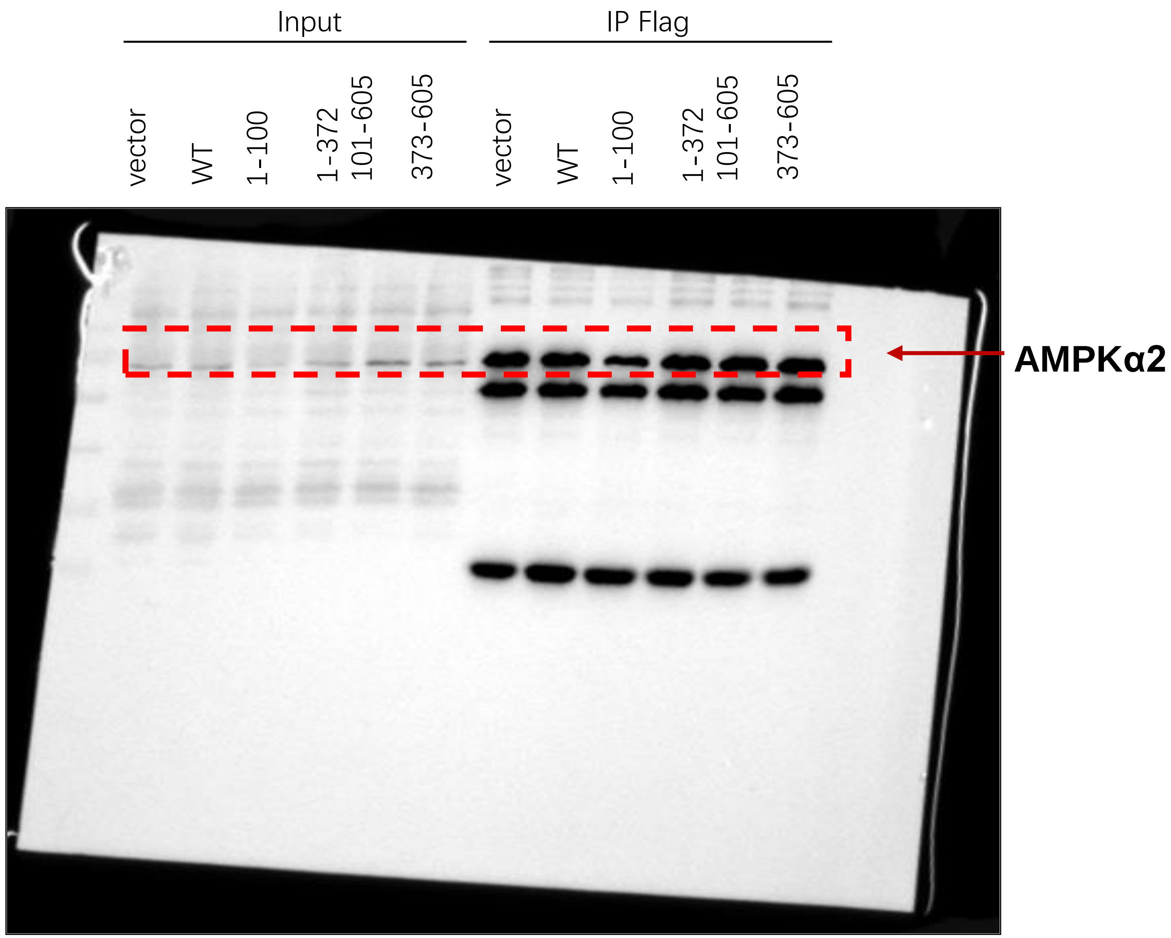

Input
IP Flag
vector
WT
1-100
1-372
101-605
373-605
vector
WT
1-100
1-372
101-605
373-605
AMPKα2

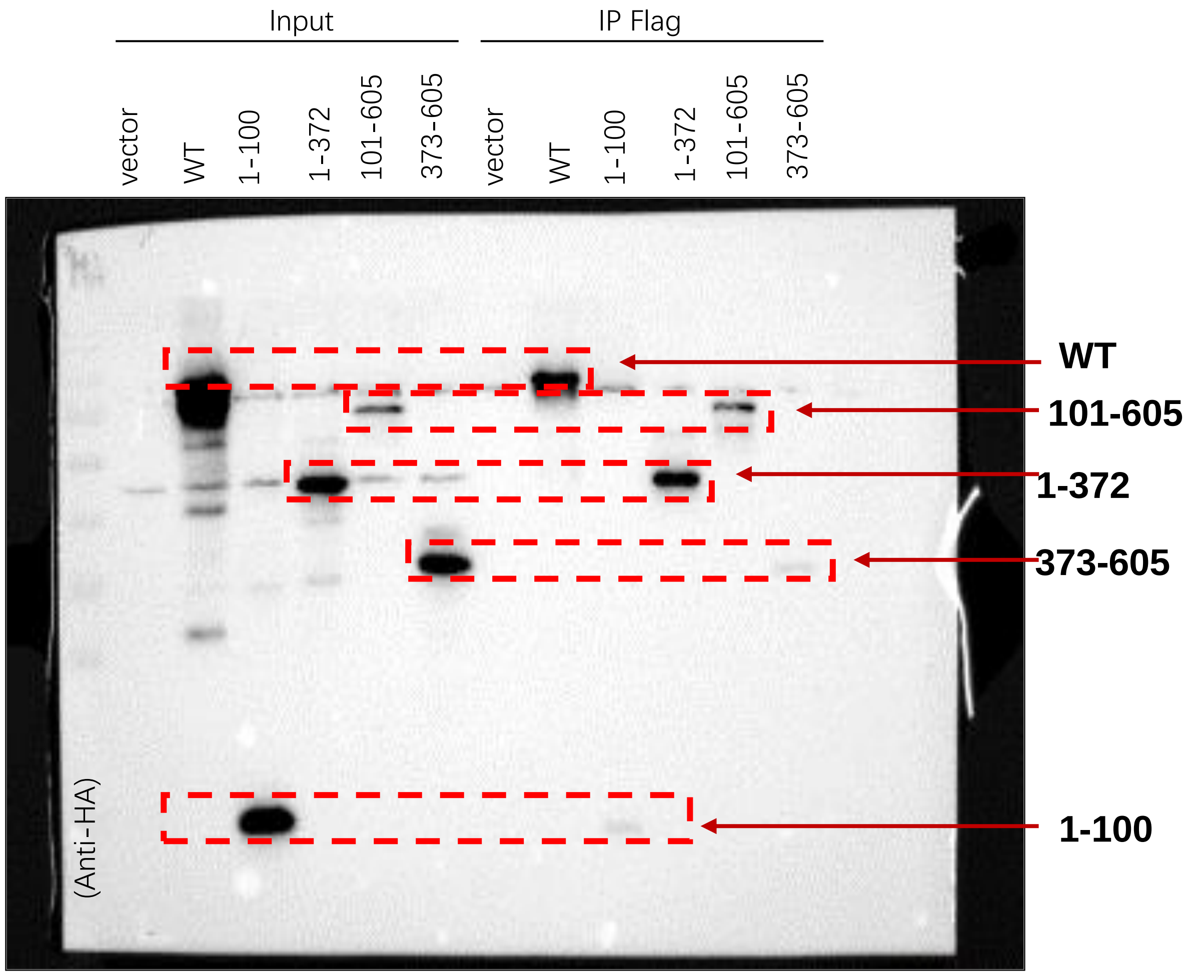

Input
IP Flag
vector
WT
1-100
1-372
101-605
373-605
vector
WT
1-100
1-372
101-605
373-605
(Anti-HA)
WT
101-605
1-372
373-605
1-100

Figure 4A

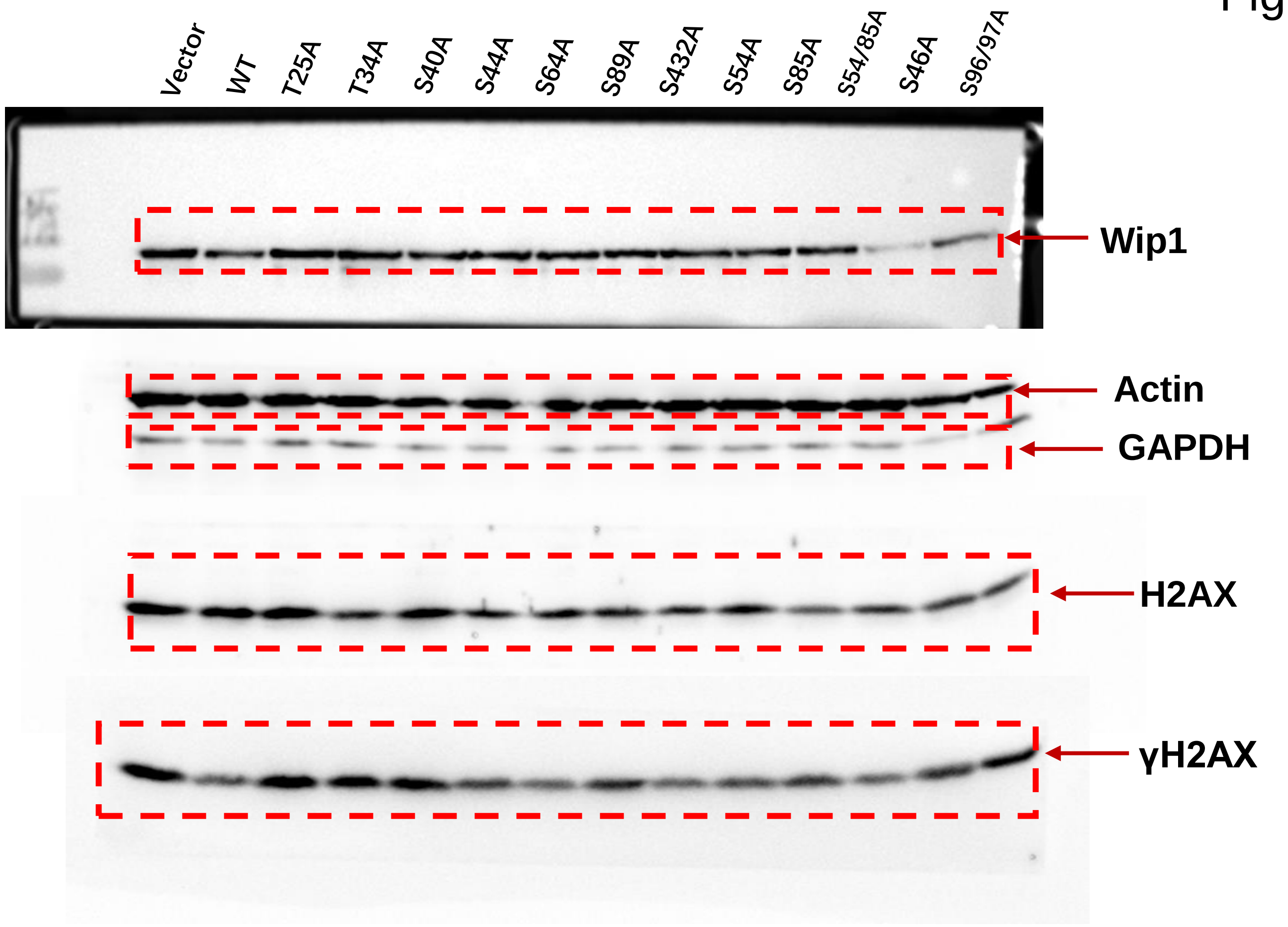

Figure 4E

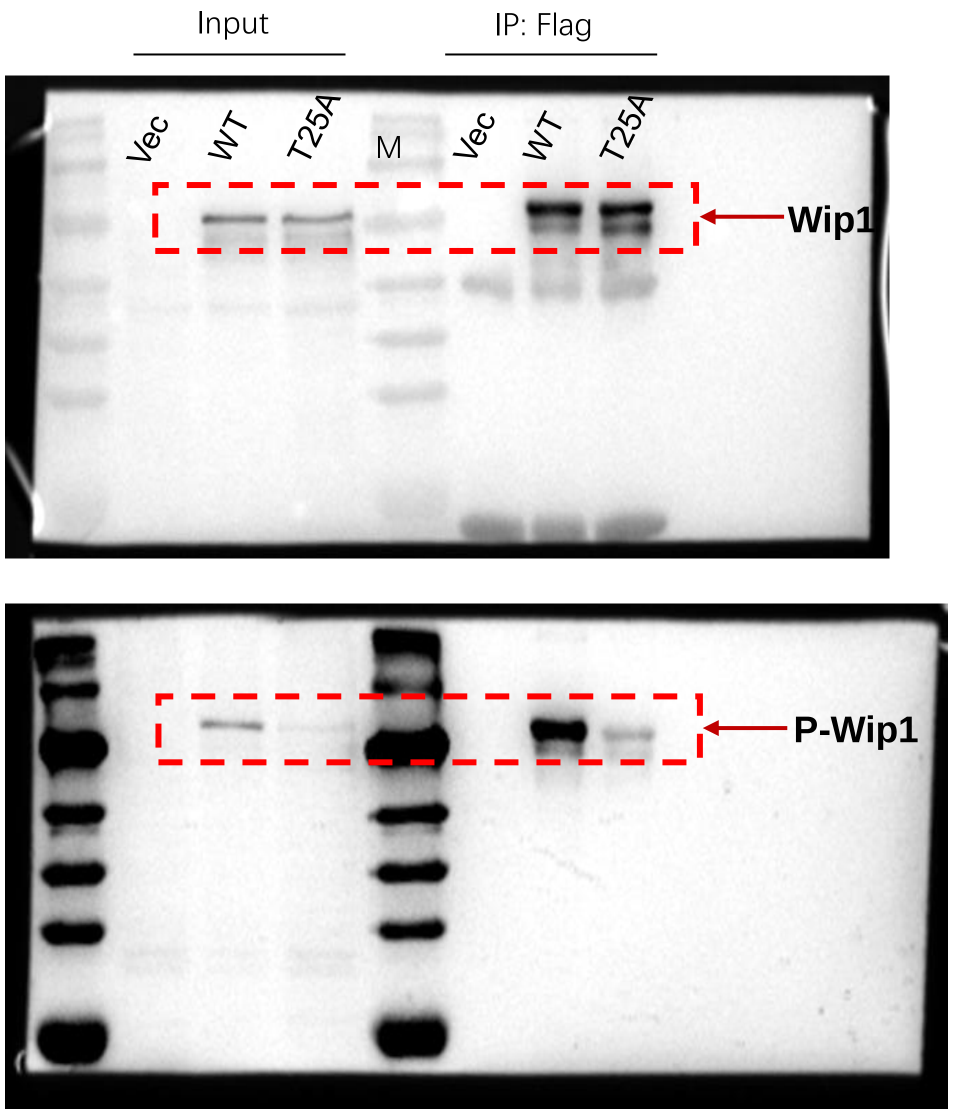

Figure 4F

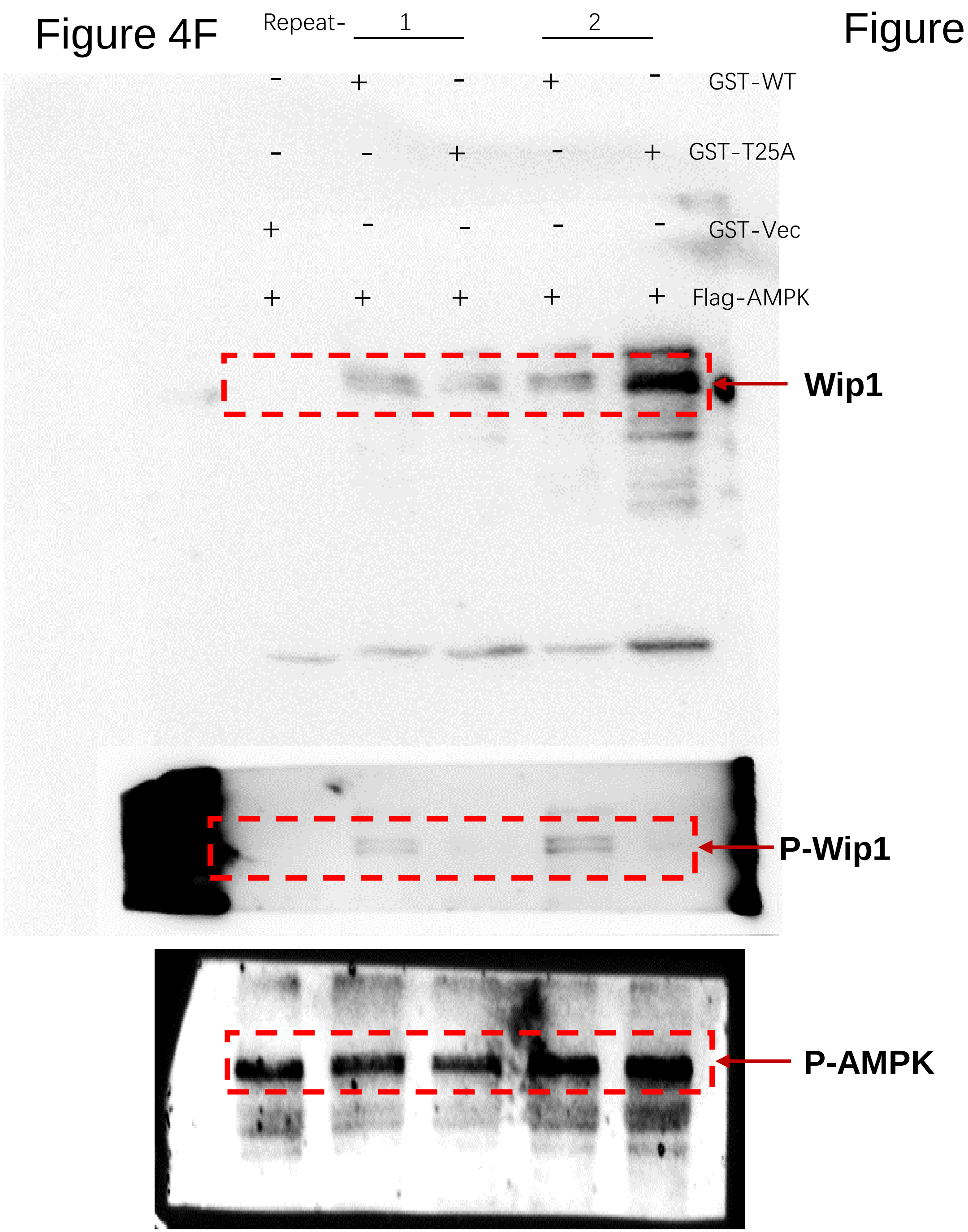

Figure 4G

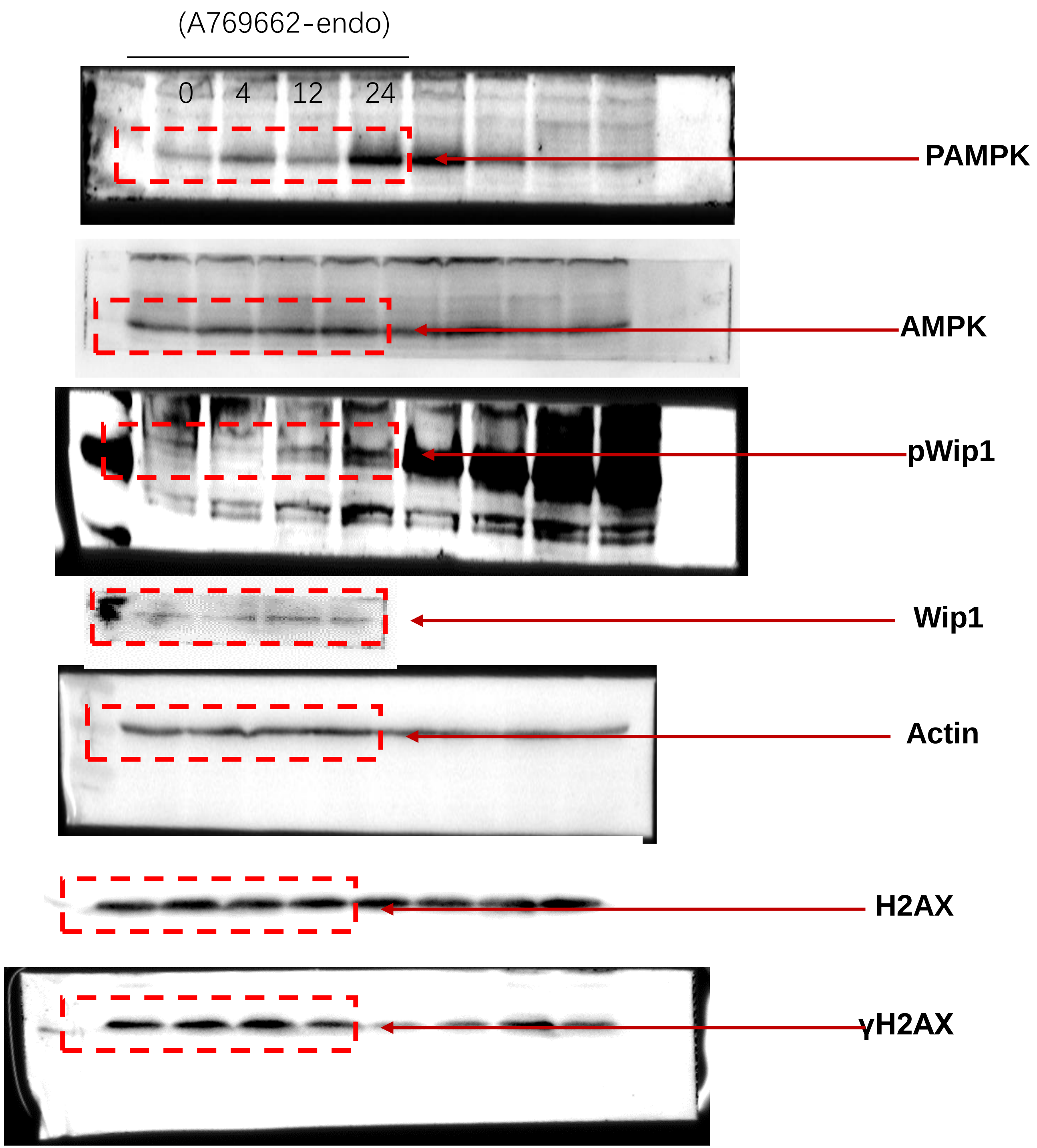

Figure 4H

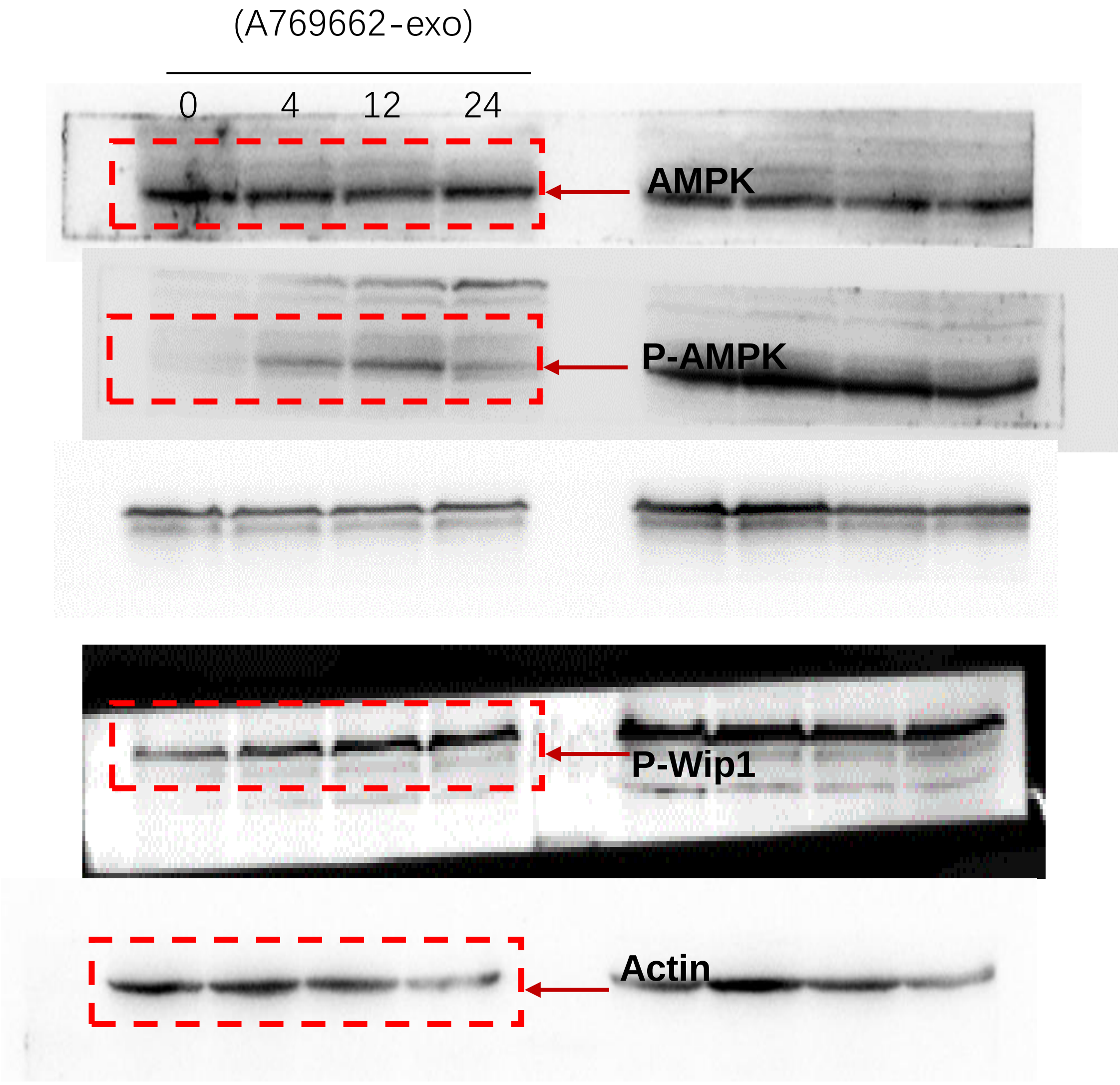

Figure 4I

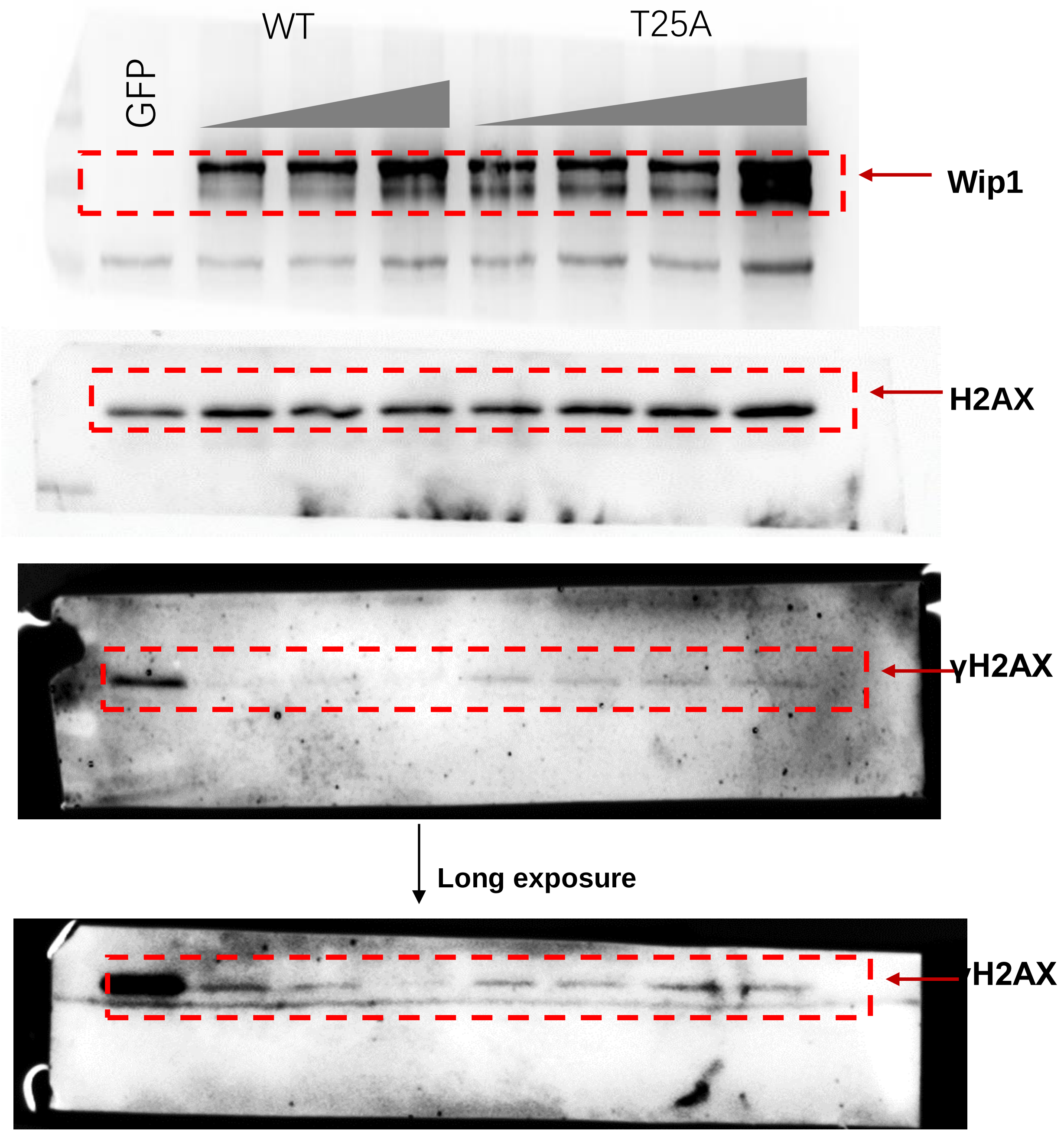

Figure 4J

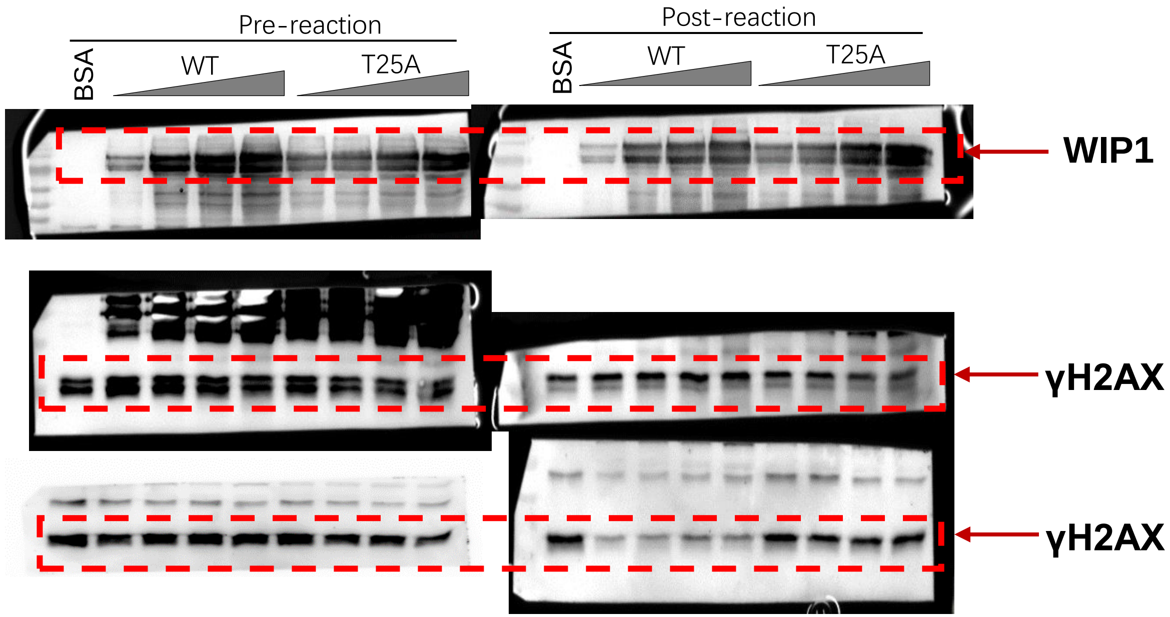

Pre-reaction
BSA
WT
T25A
Post-reaction
BSA
WT
T25A
WIP1
γH2AX
γH2AX

Figure 5A

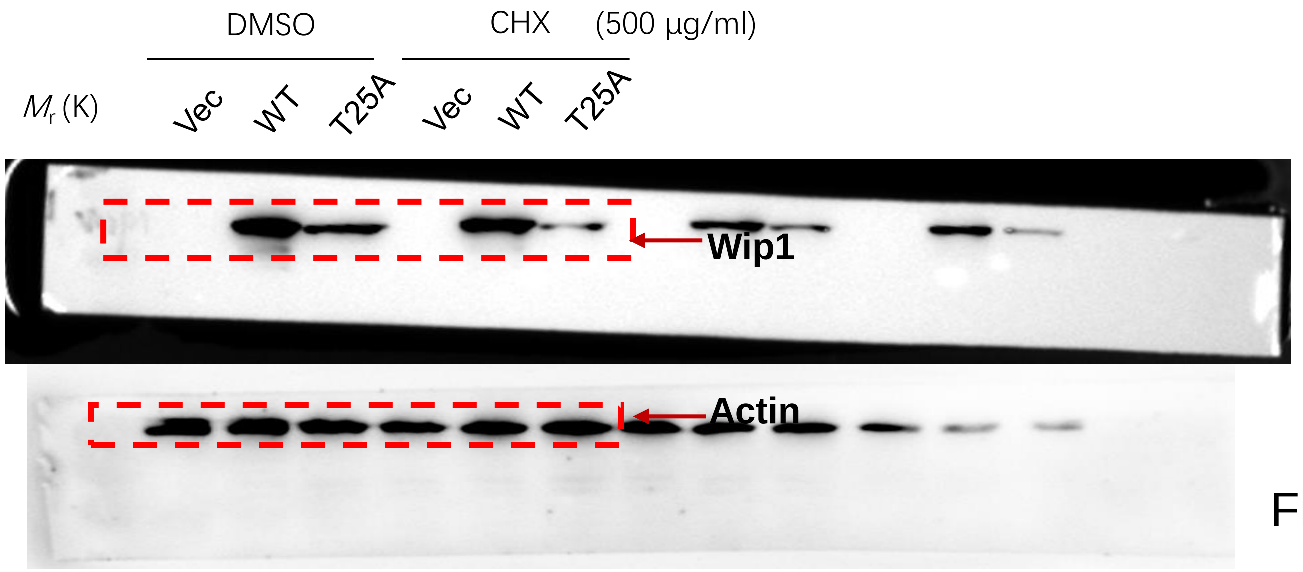

Figure5B

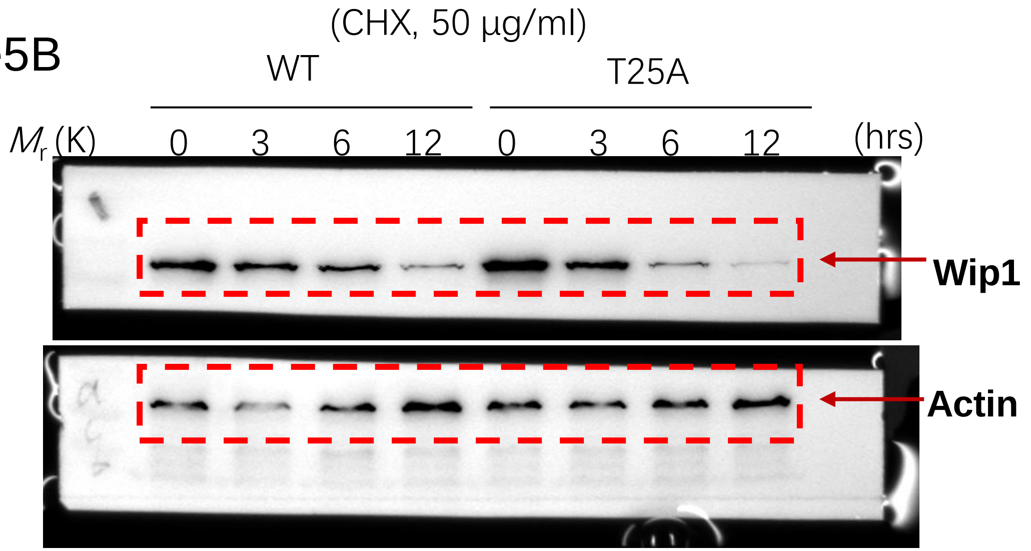

Figure5C

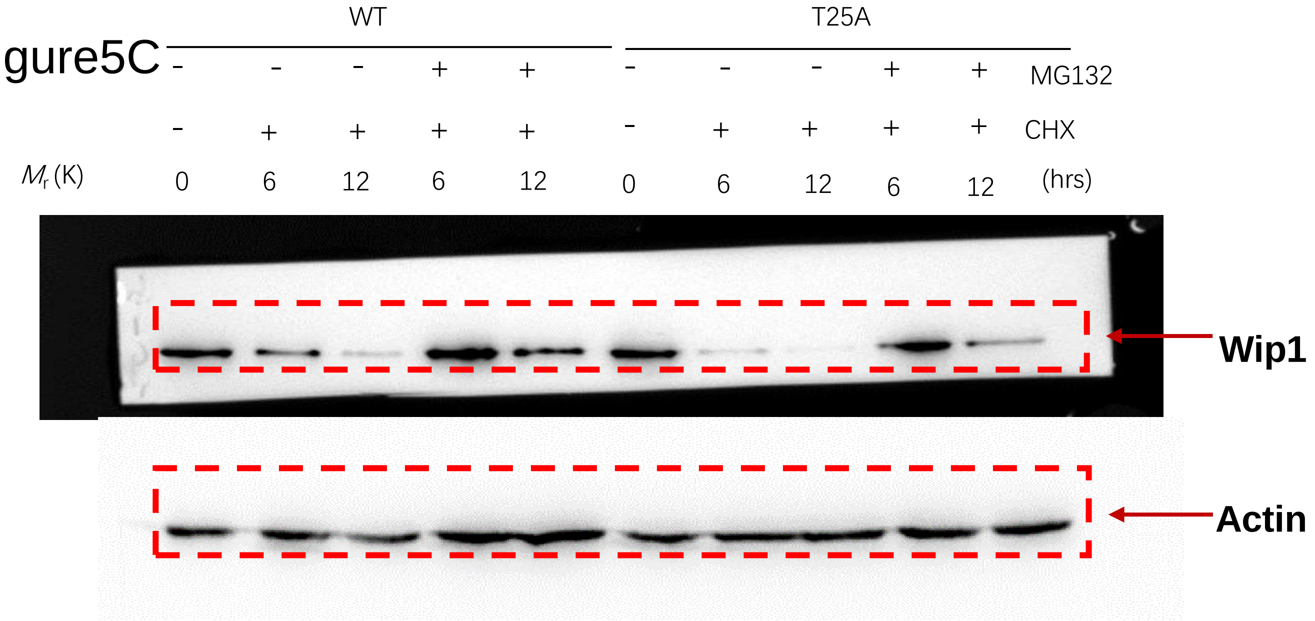

Figure5D

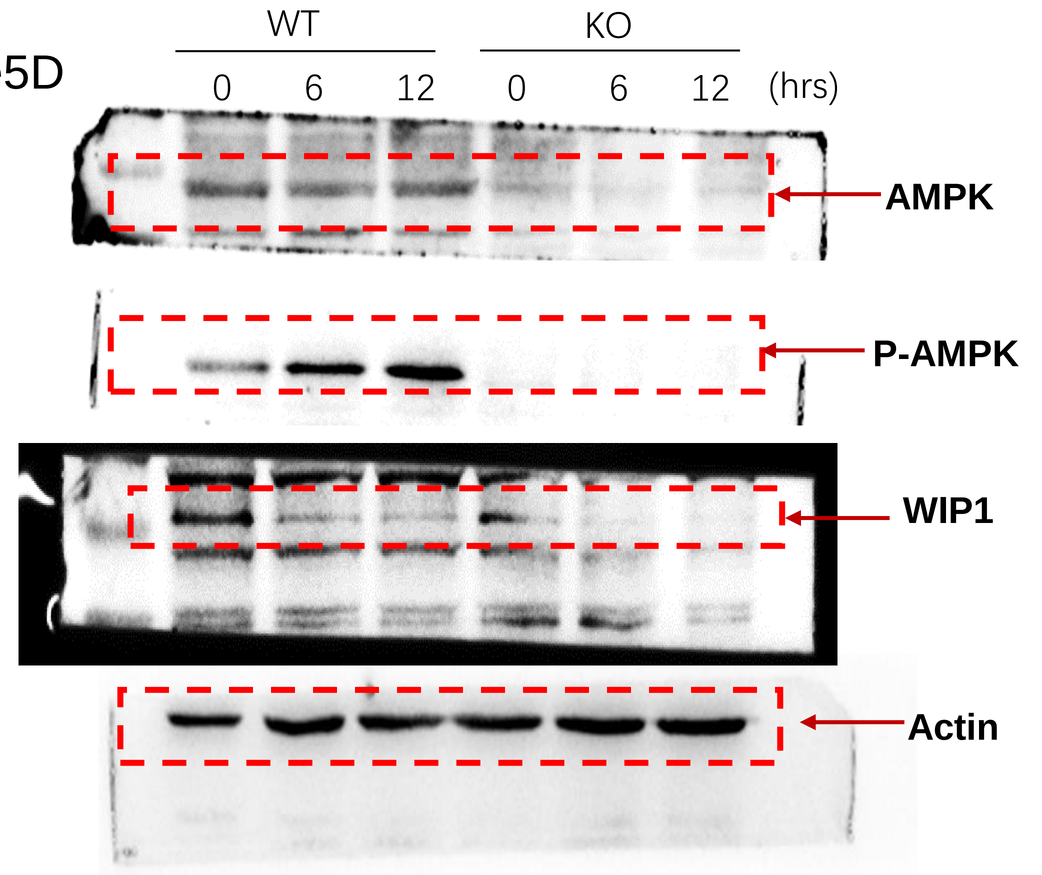

Figure5E

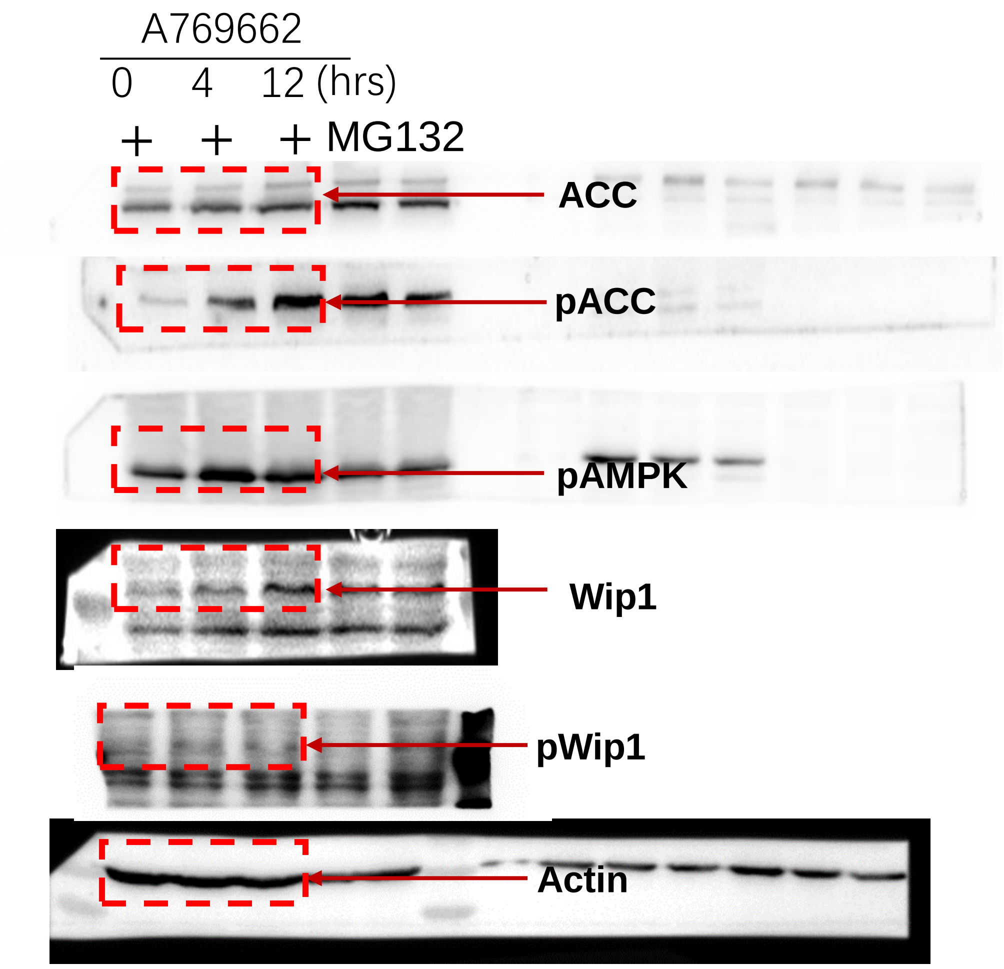

Figure5F

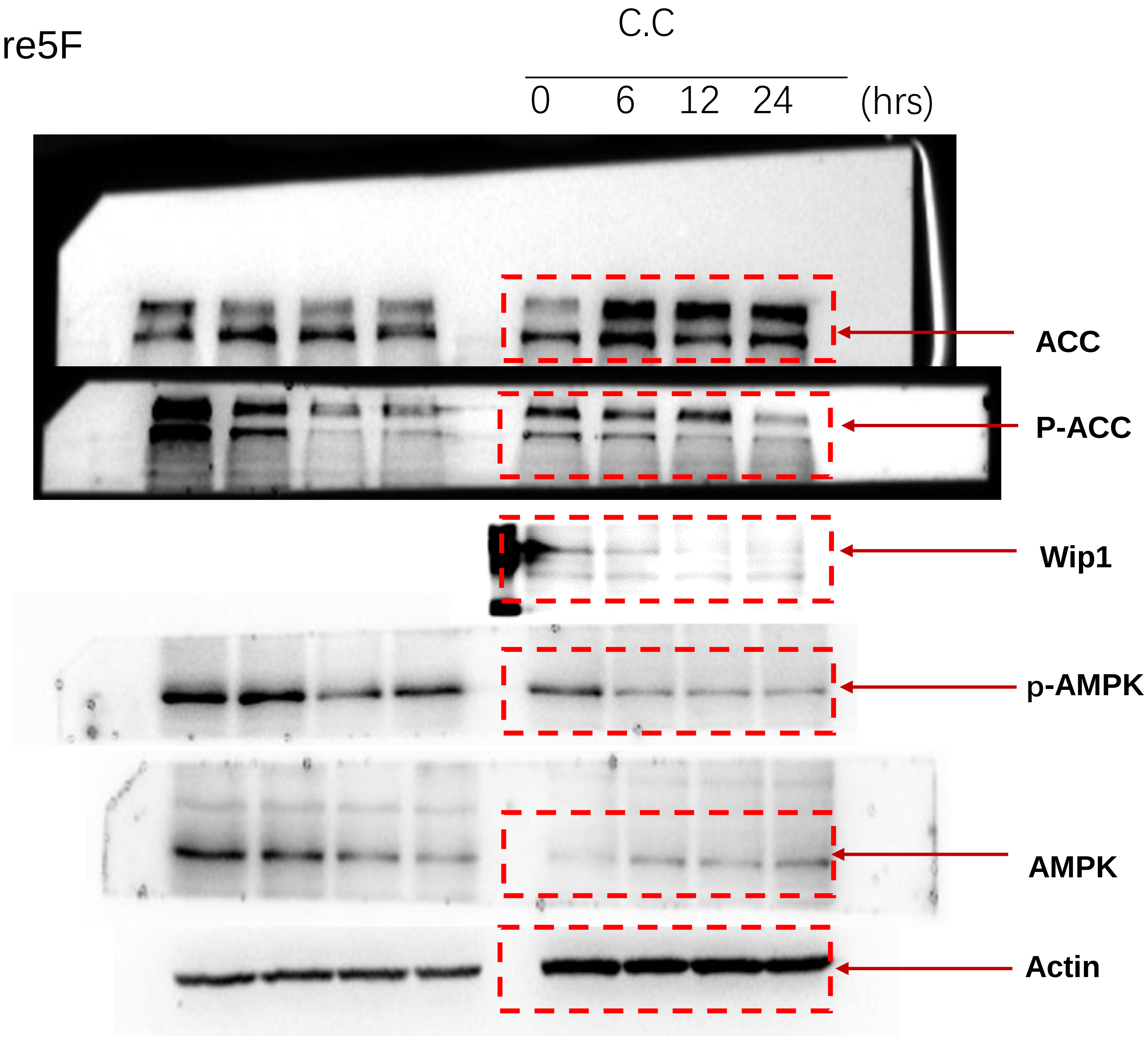

Figure5G

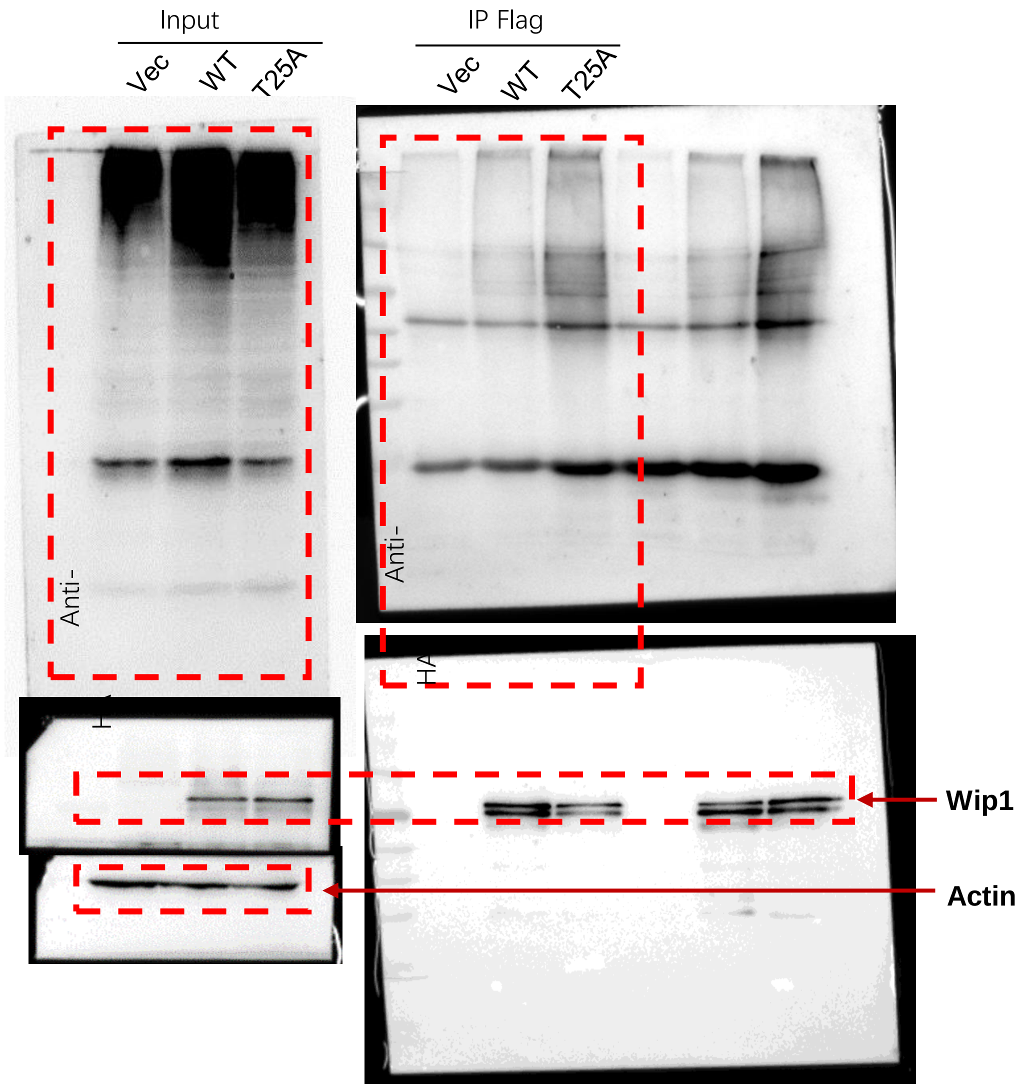

Figure5H

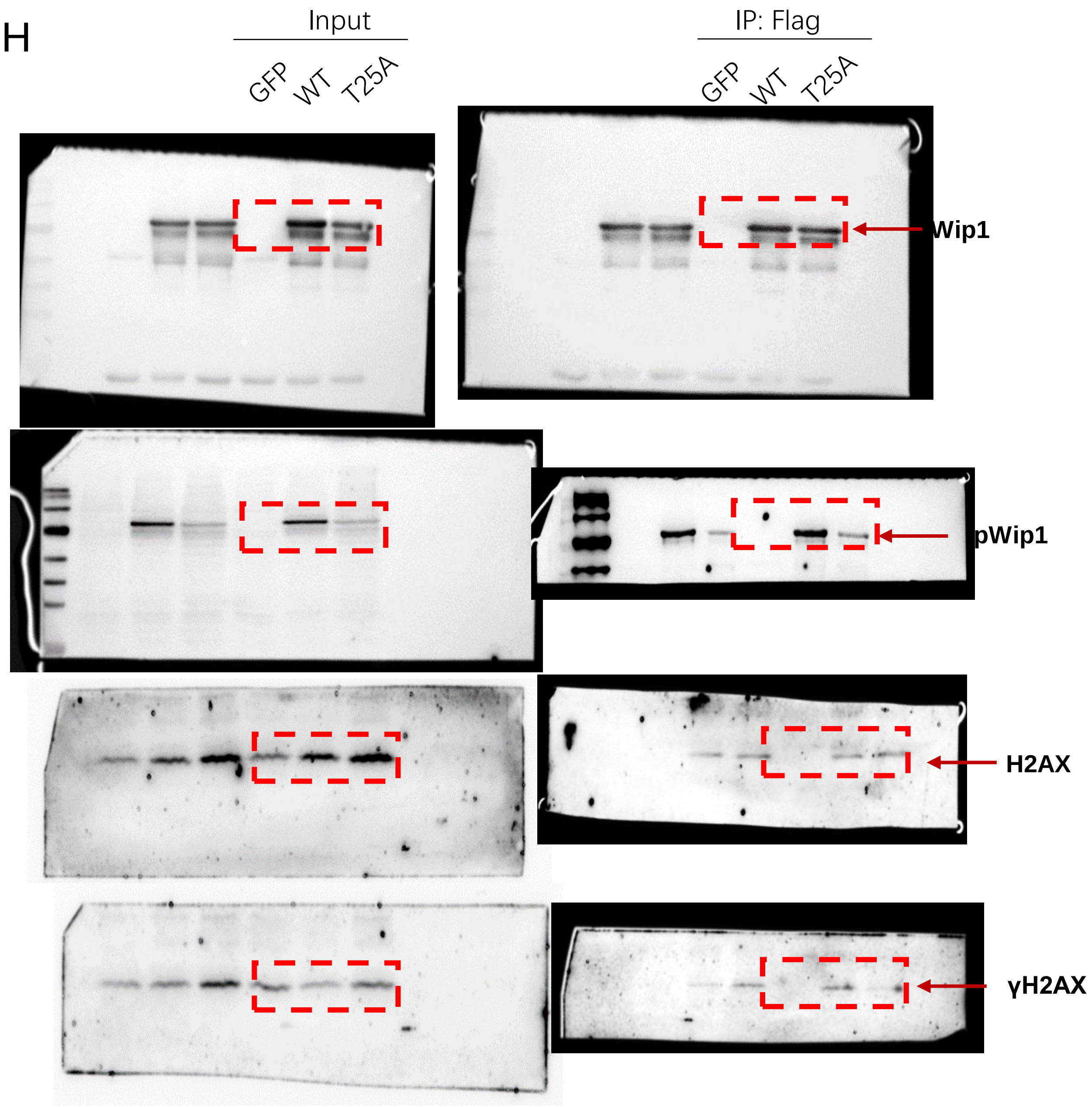

Figure 5I

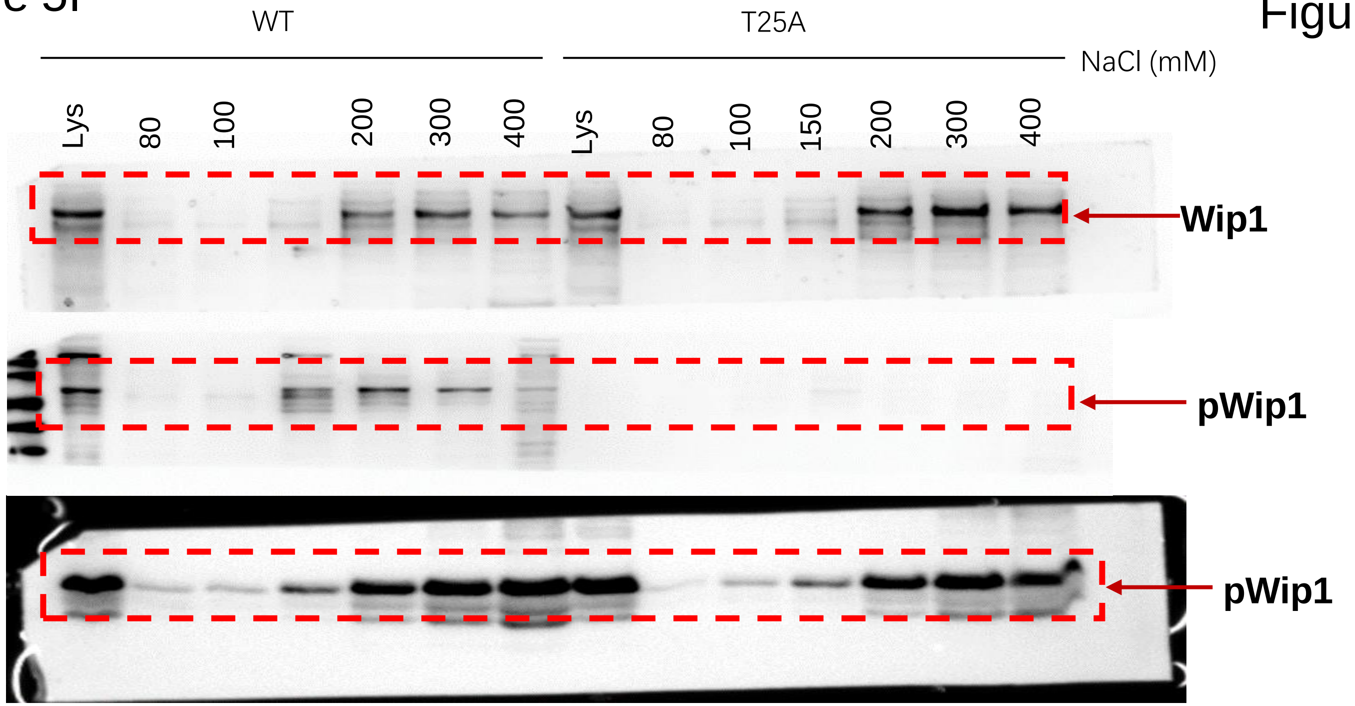

Figure 6D

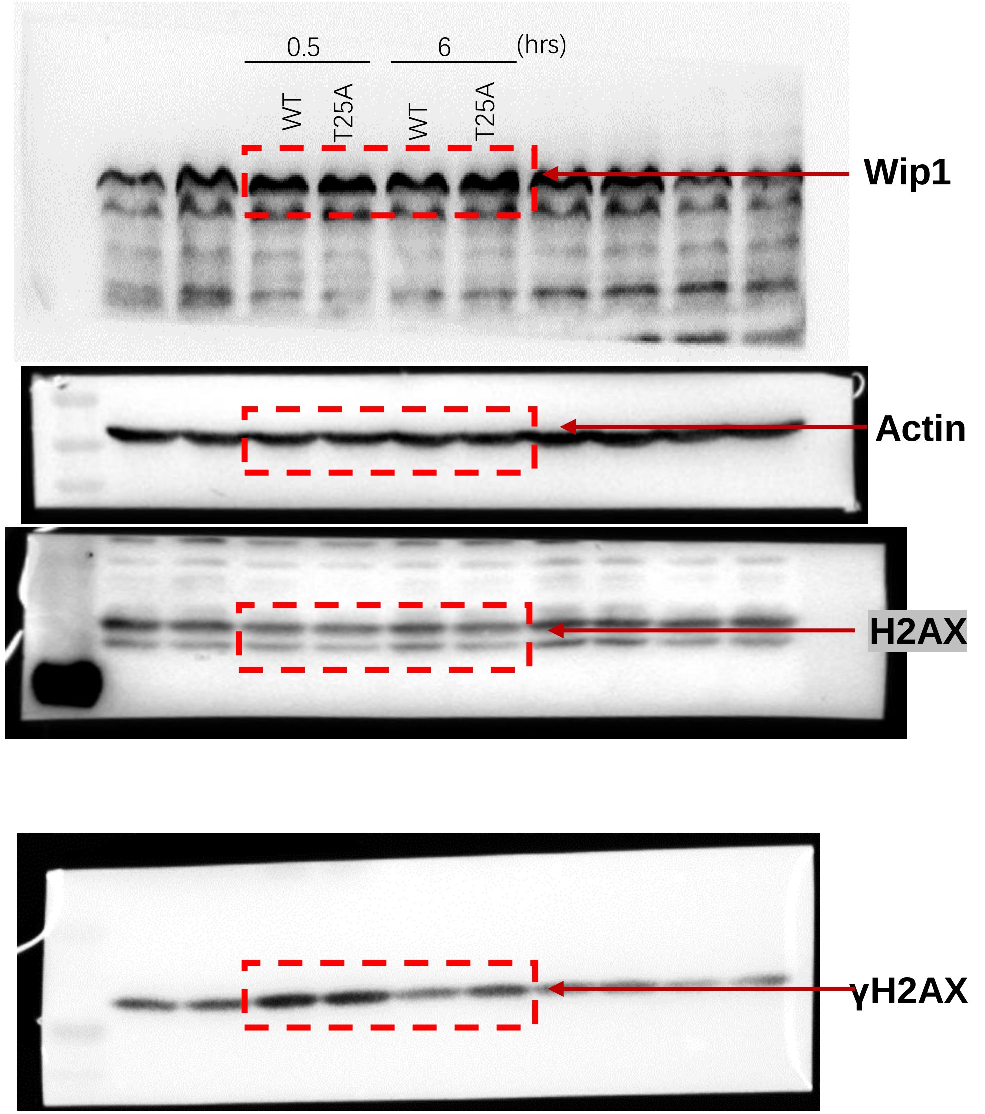

Figure 6A

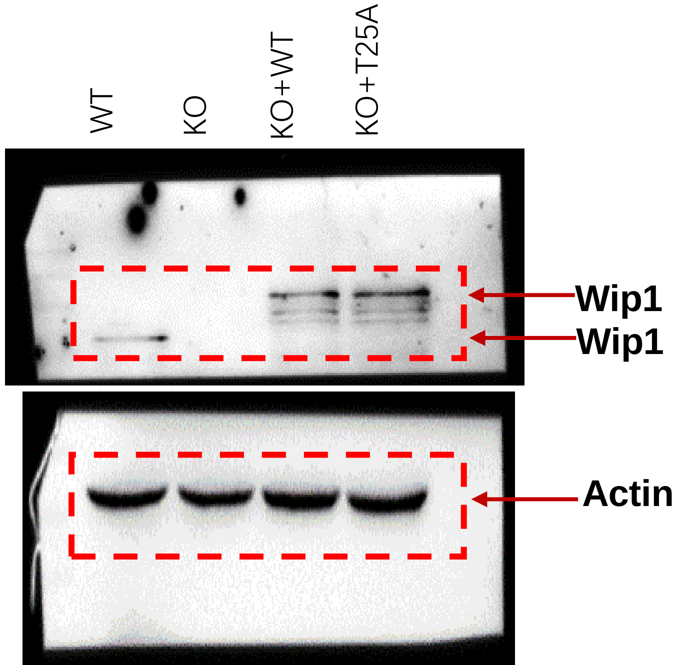

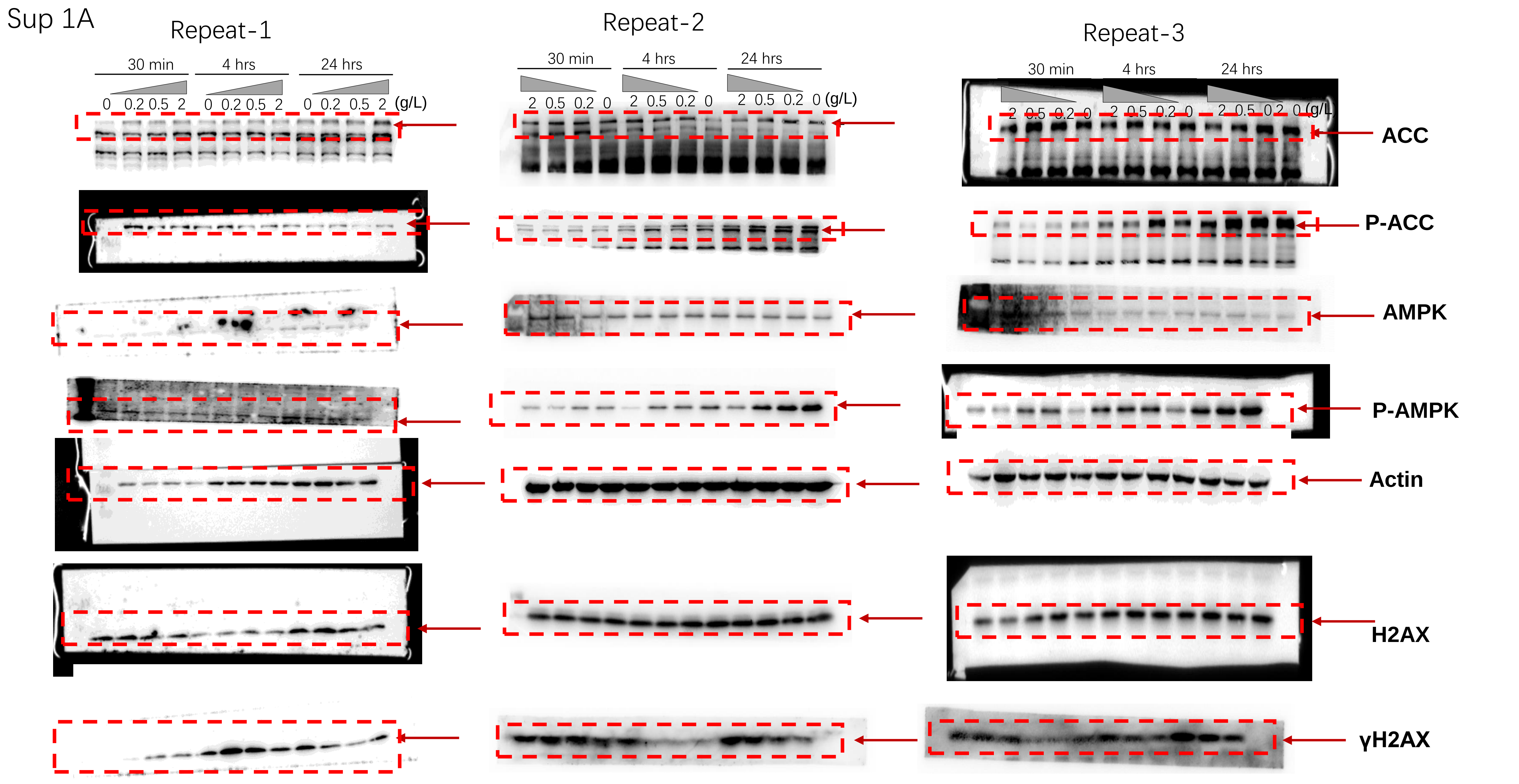
Sup 1A
Repeat-1
30 min
4 hrs
24 hrs
0 0.2 0.5 2 0 0.2 0.5 2 0 0.2 0.5 2 (g/L)
Repeat-2
30 min
4 hrs
24 hrs
2 0.5 0.2 0 2 0.5 0.2 0 2 0.5 0.2 0 (g/L)
Repeat-3
30 min
4 hrs
24 hrs
2 0.5 0.2 0 2 0.5 0.2 0 2 0.5 0.2 0 (g/L)
ACC
P-ACC
AMPK
P-AMPK
Actin
H2AX
γH2AX

Sup 1B

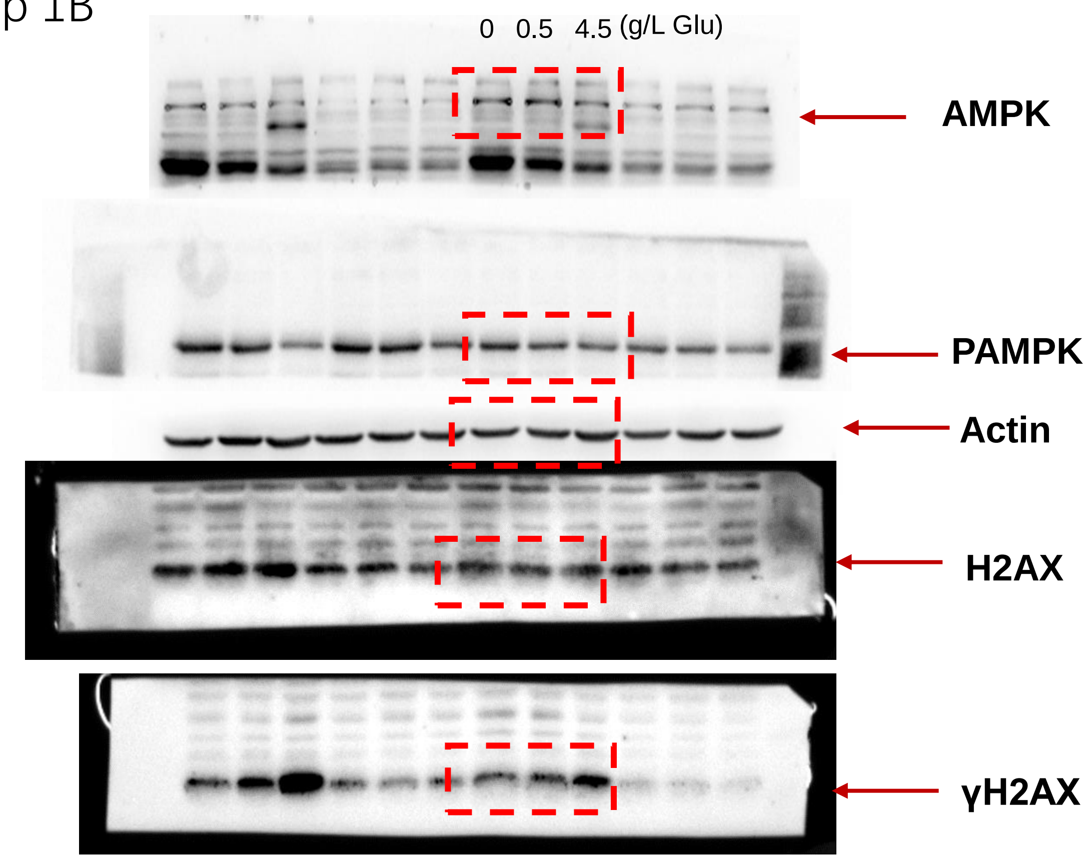

Sup 1D

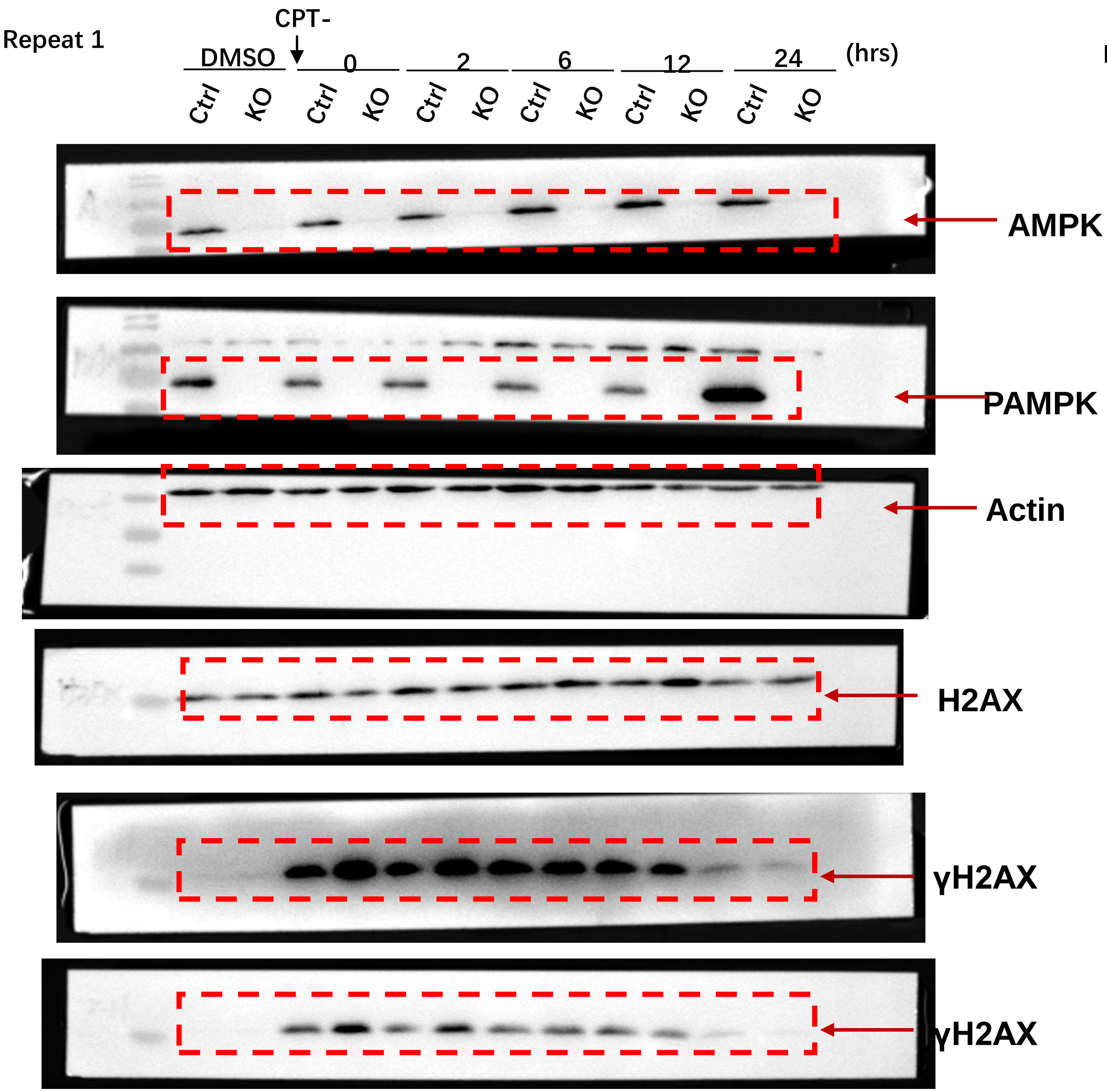

Repeat 1
CPT-
DMSO
0
2
6
12
24
(hrs)
Ctrl
KO
Ctrl
KO
Ctrl
KO
Ctrl
KO
Ctrl
KO
Ctrl
KO
AMPK
PAMPK
Actin
H2AX
γH2AX
γH2AX

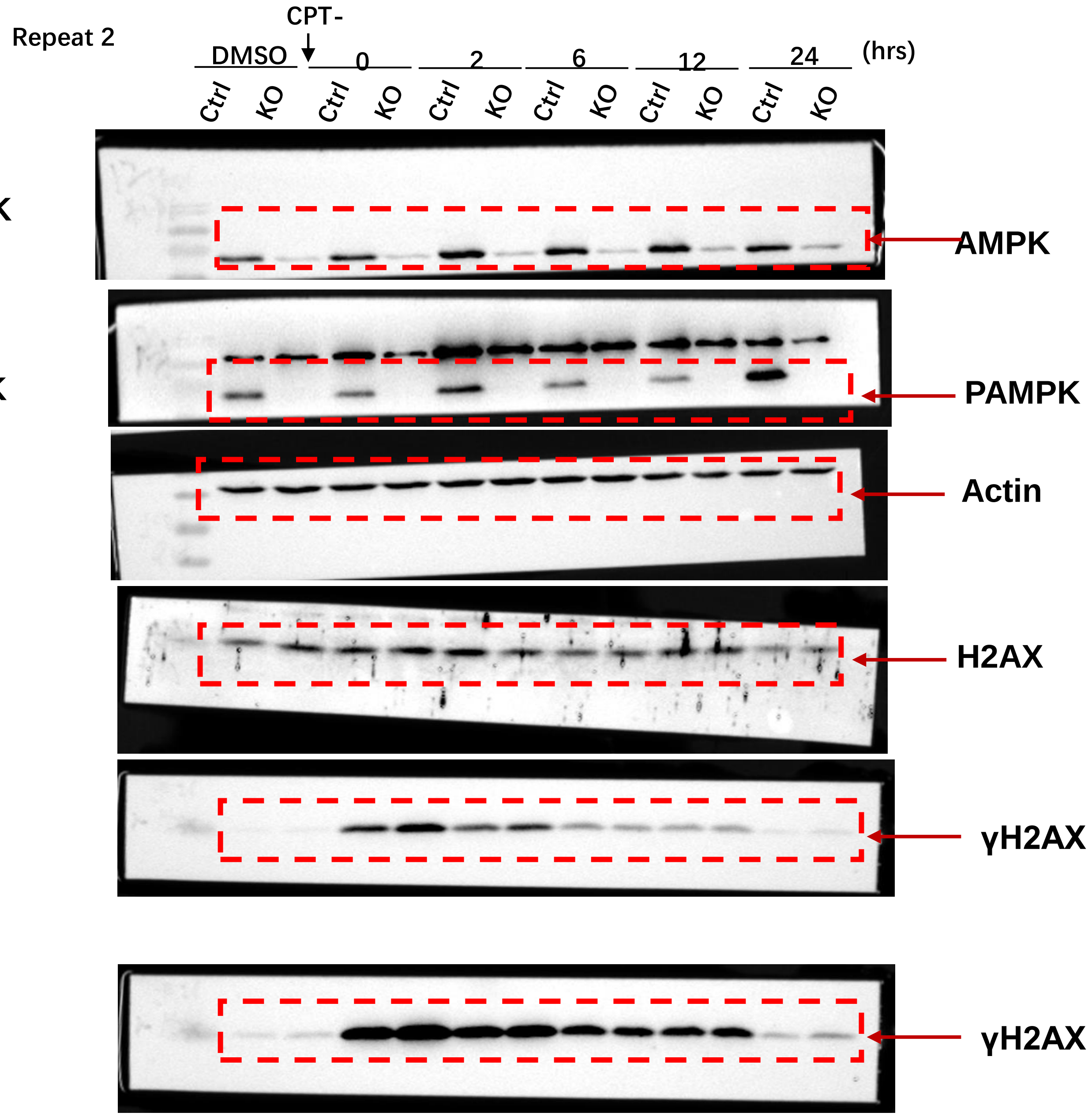

Repeat 2
CPT-
DMSO
0
2
6
12
24
(hrs)
Ctrl
KO
Ctrl
KO
Ctrl
KO
Ctrl
KO
Ctrl
KO
Ctrl
KO
AMPK
PAMPK
Actin
H2AX
γH2AX
γH2AX

Sup 1E

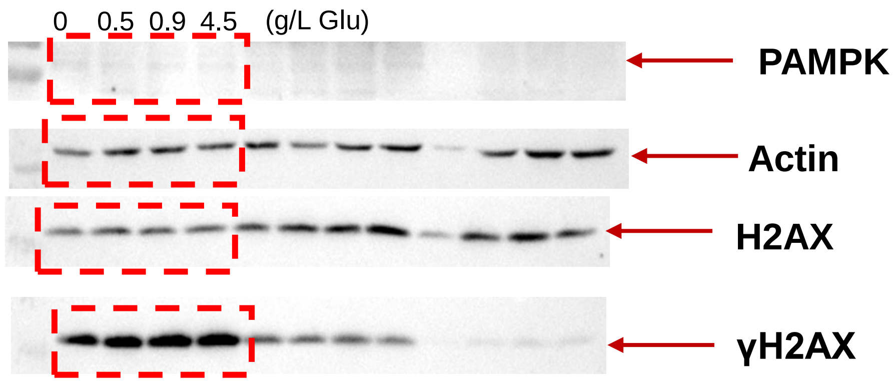
0 0.5 0.9 4.5 (g/L Glu)
PAMPK
Actin
H2AX
γH2AX

Sup 1F

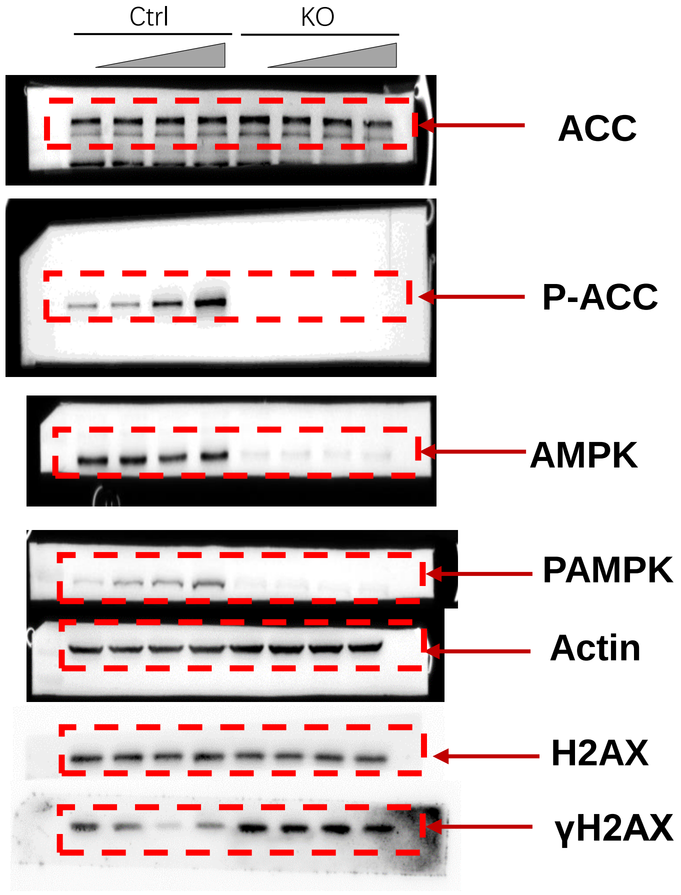
Ctrl
KO
ACC
P-ACC
AMPK
PAMPK
Actin
H2AX
γH2AX

Sup 2A

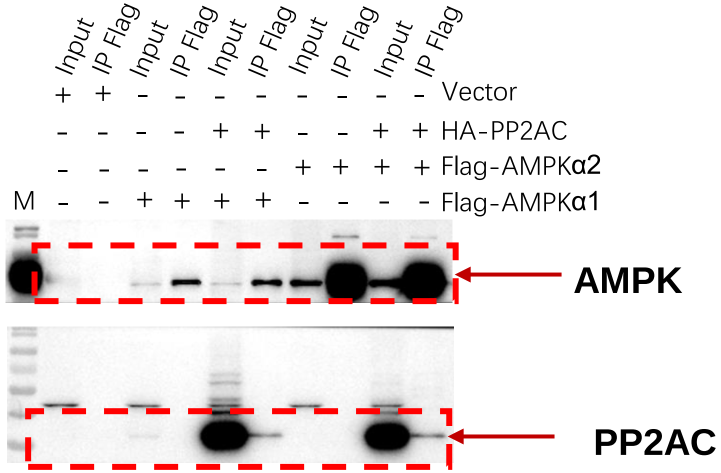

Sup 2B

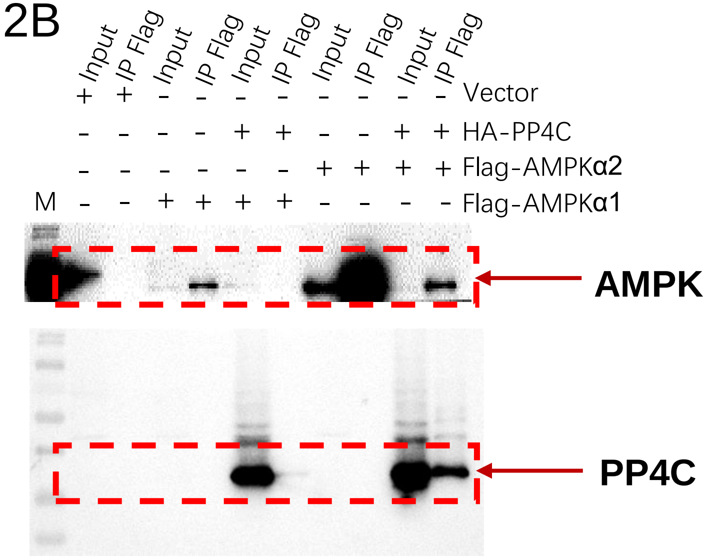

Sup 2C

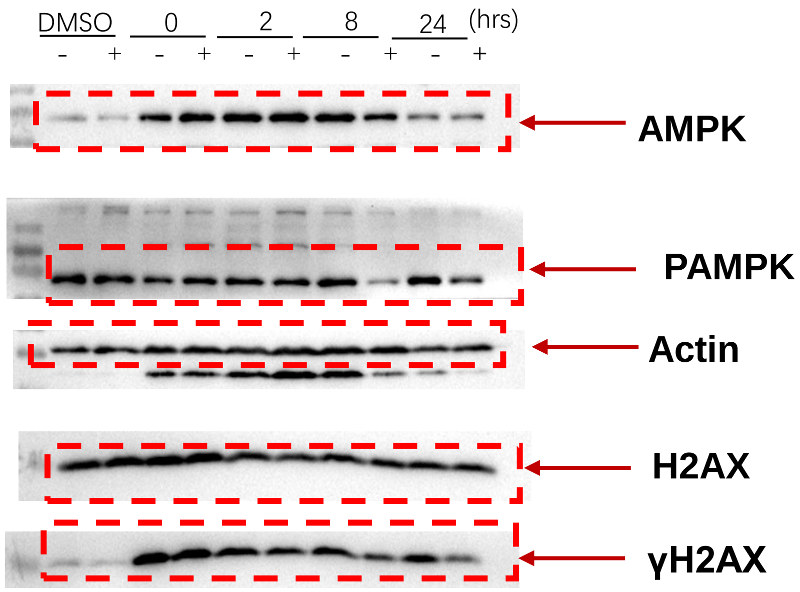

Sup 2D

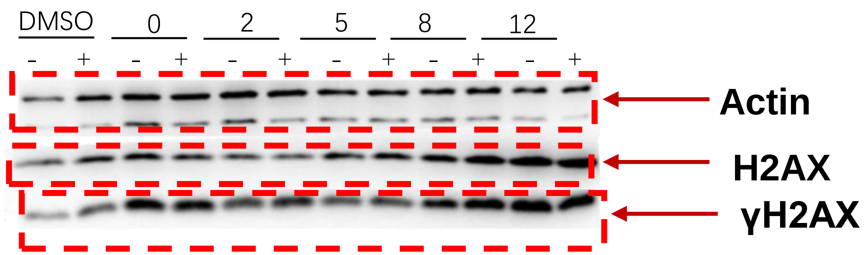

Sup 2E

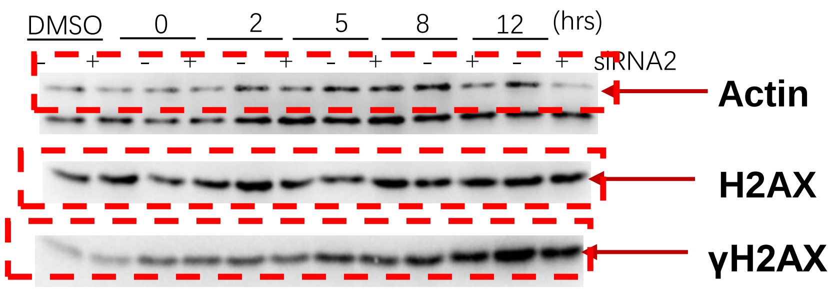

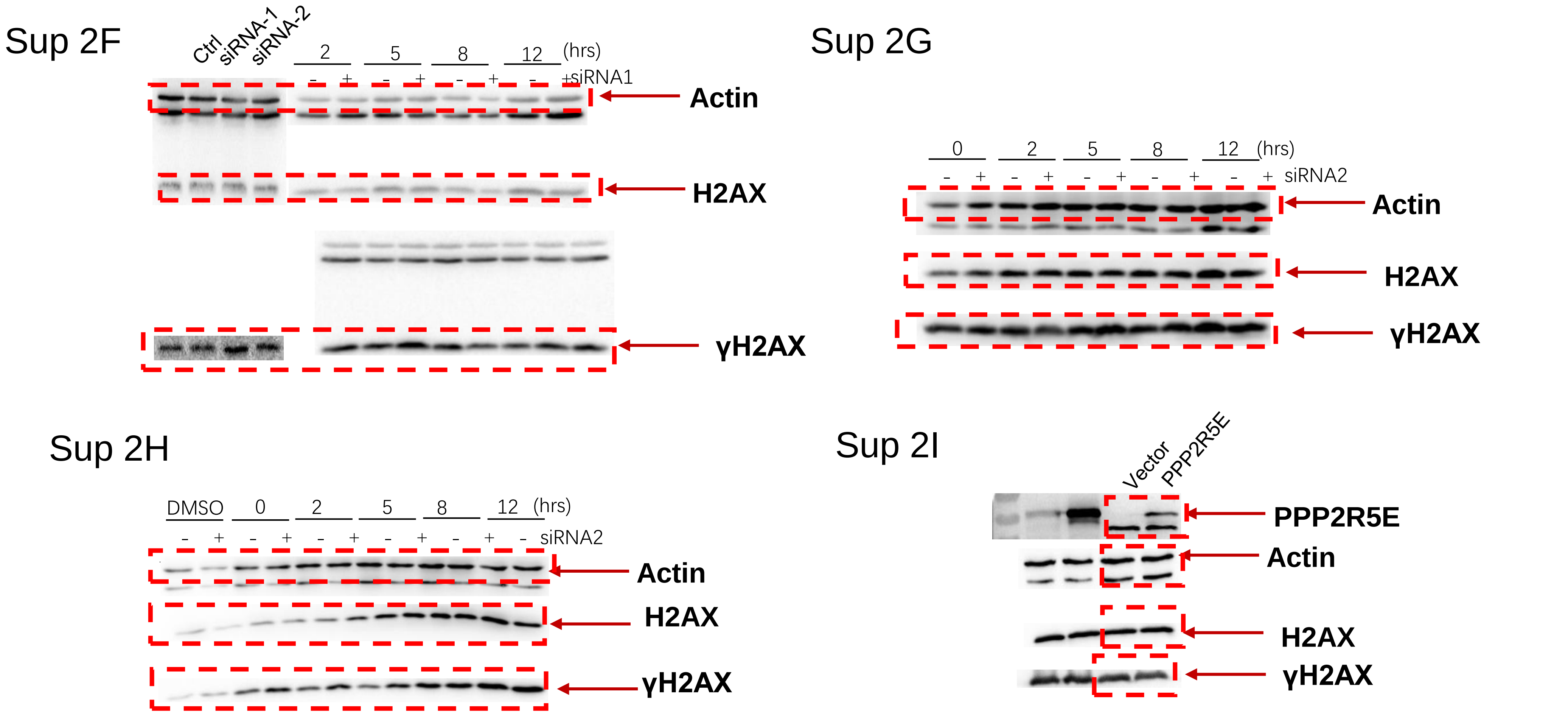
Sup 2F
Ctrl
siRNA-1
siRNA-2
2
5
8
12
(hrs)
- + - + - + - + siRNA1
Actin
H2AX
γH2AX
Sup 2G
0
2
5
8
12
(hrs)
- + - + - + - + - + siRNA2
Actin
H2AX
γH2AX
Sup 2H
DMSO
0
2
5
8
12
(hrs)
- + - + - + - + - + - siRNA2
Actin
H2AX
γH2AX
Sup 2I
Vector
PPP2R5E
PPP2R5E
Actin
H2AX
γH2AX

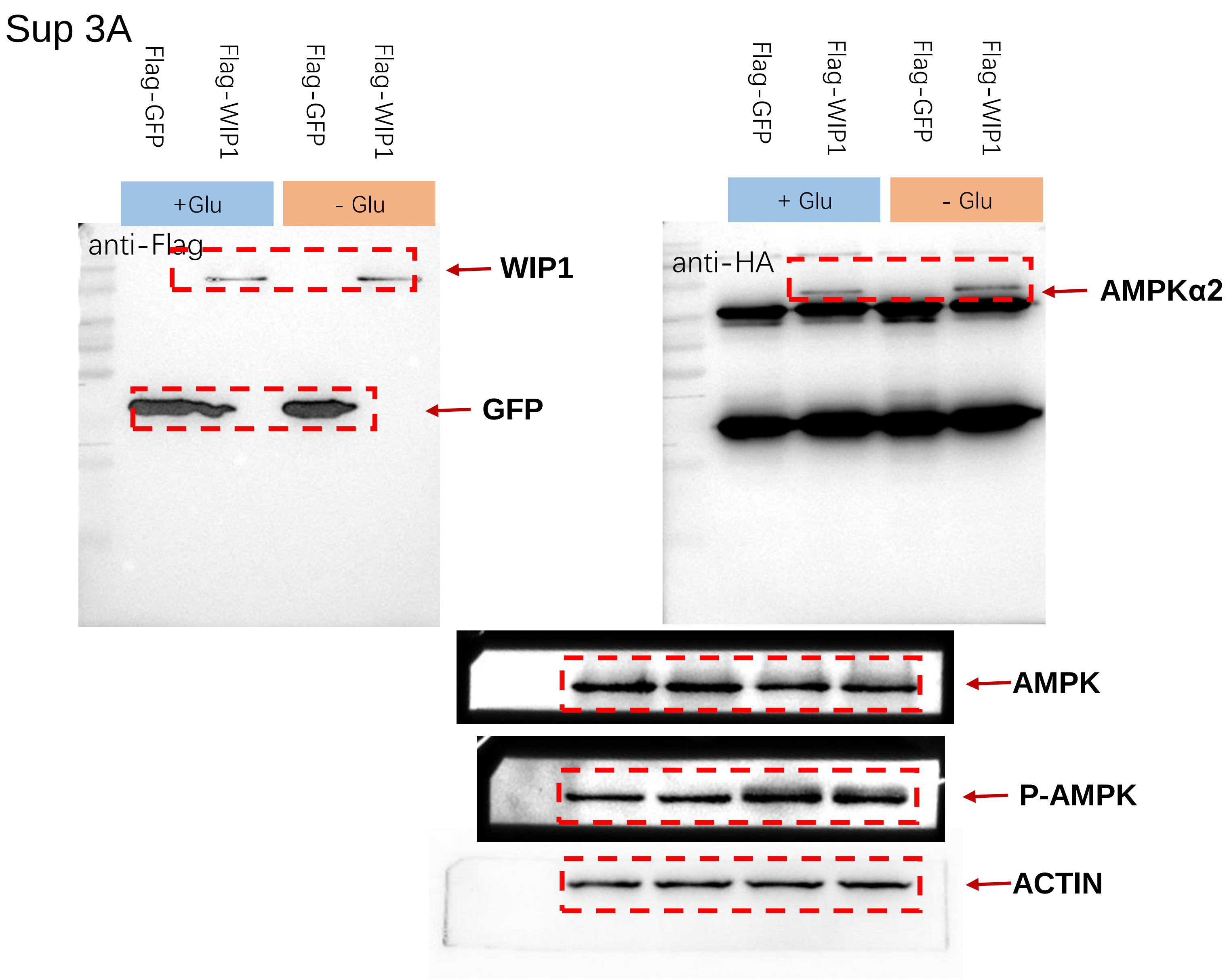
Sup 3A
Flag-GFP
Flag-WIP1
Flag-GFP
Flag-WIP1
+Glu
- Glu
anti-Flag
WIP1
GFP
Flag-GFP
Flag-WIP1
Flag-GFP
Flag-WIP1
+ Glu
- Glu
anti-HA
AMPKα2
AMPK
P-AMPK
ACTIN

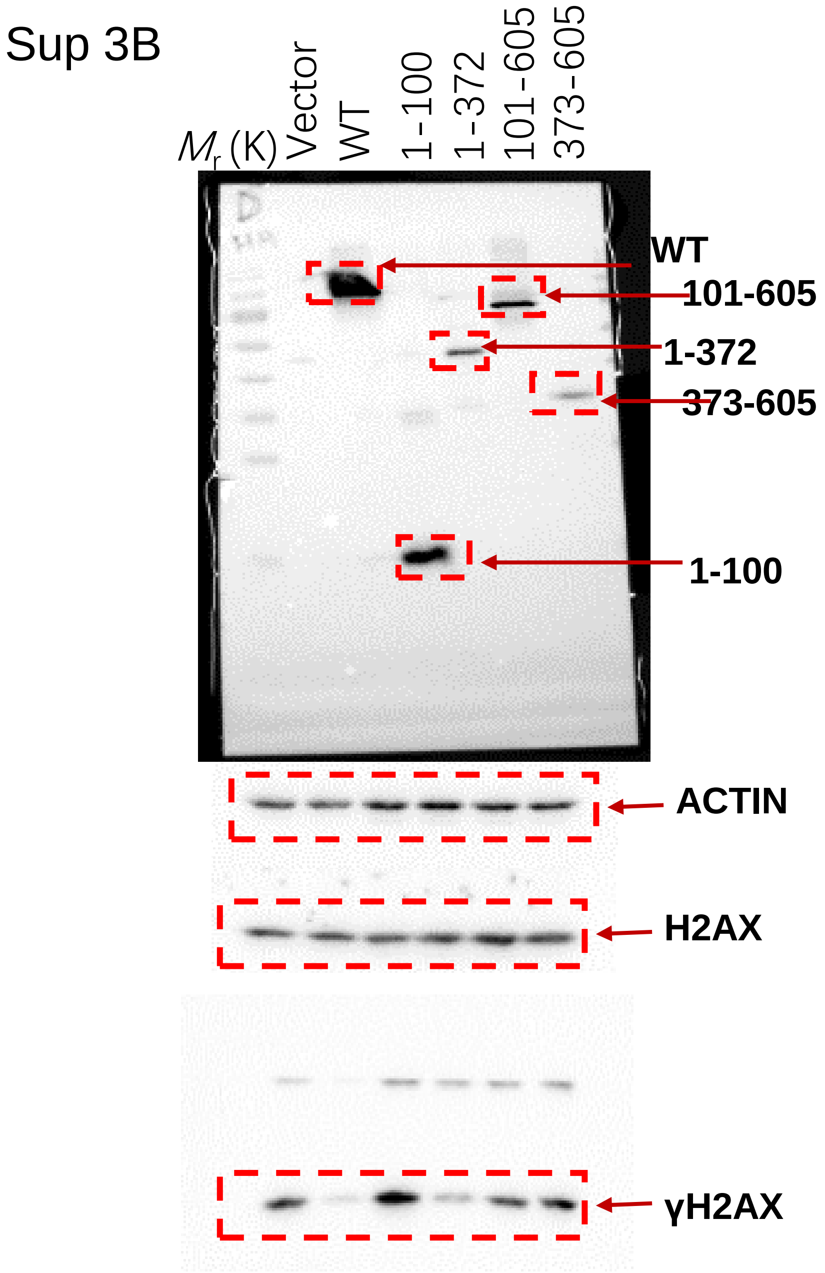
Sup 3B
Mr (K)
Vector
WT
1-100
1-372
101-605
373-605
WT
101-605
1-372
373-605
1-100
ACTIN
H2AX
γH2AX

Sup 4A

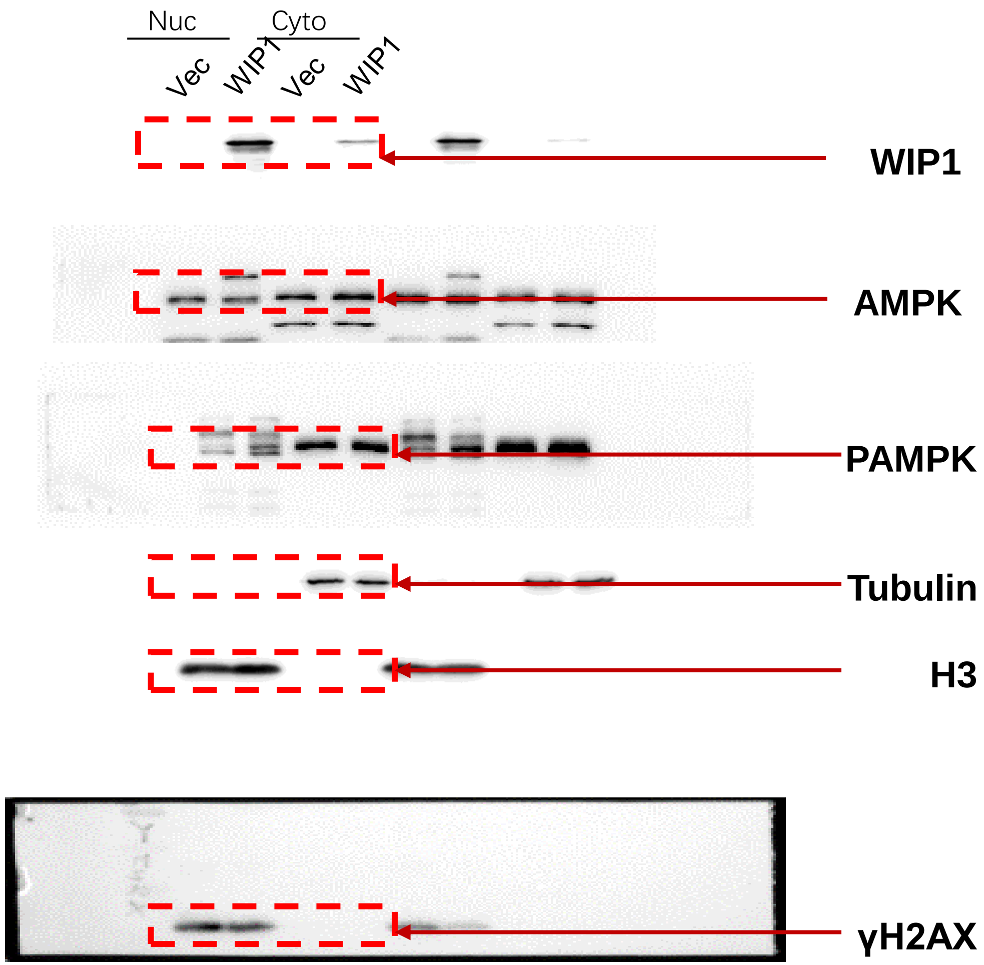

Sup 4B

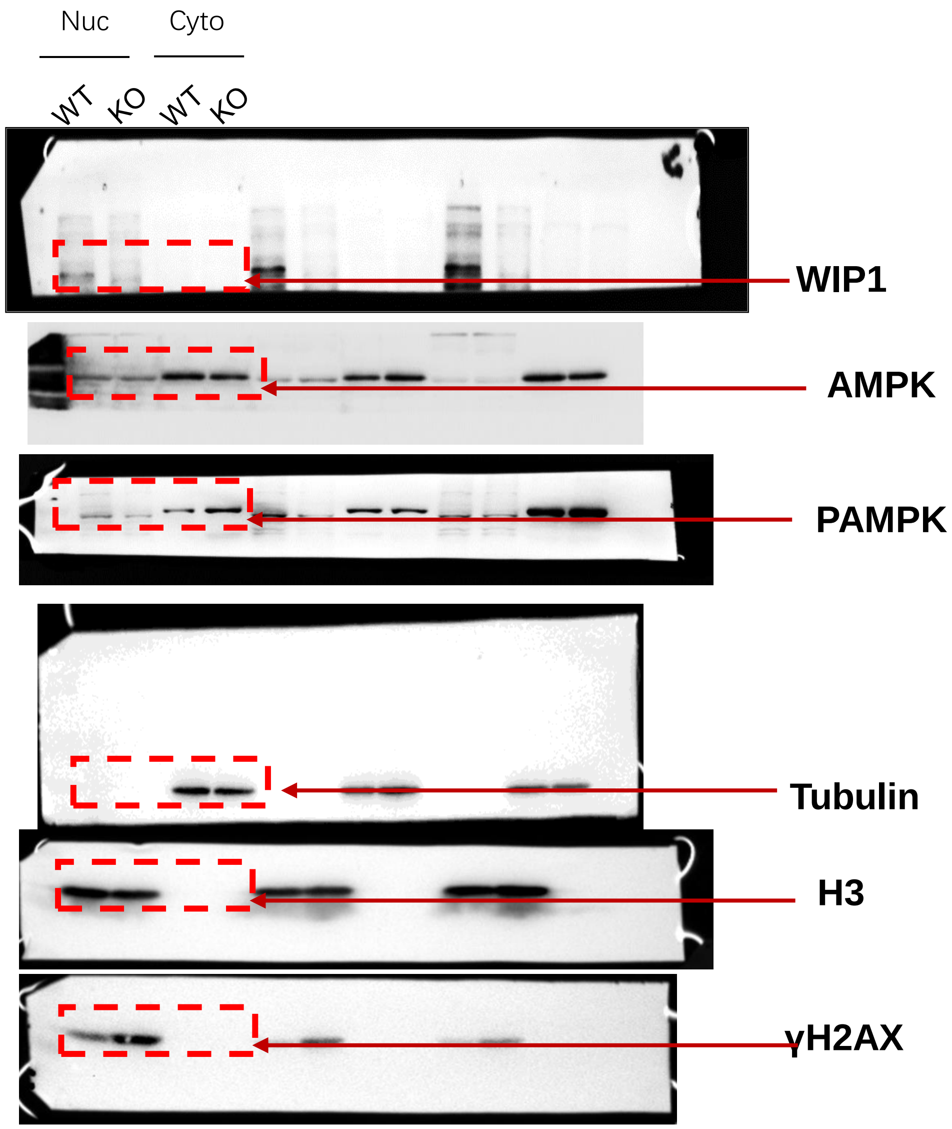

Sup 5A

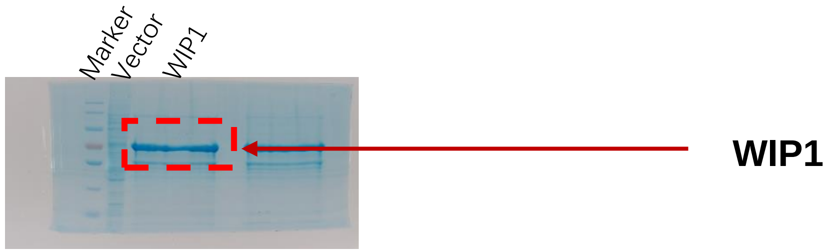

Sup 6A

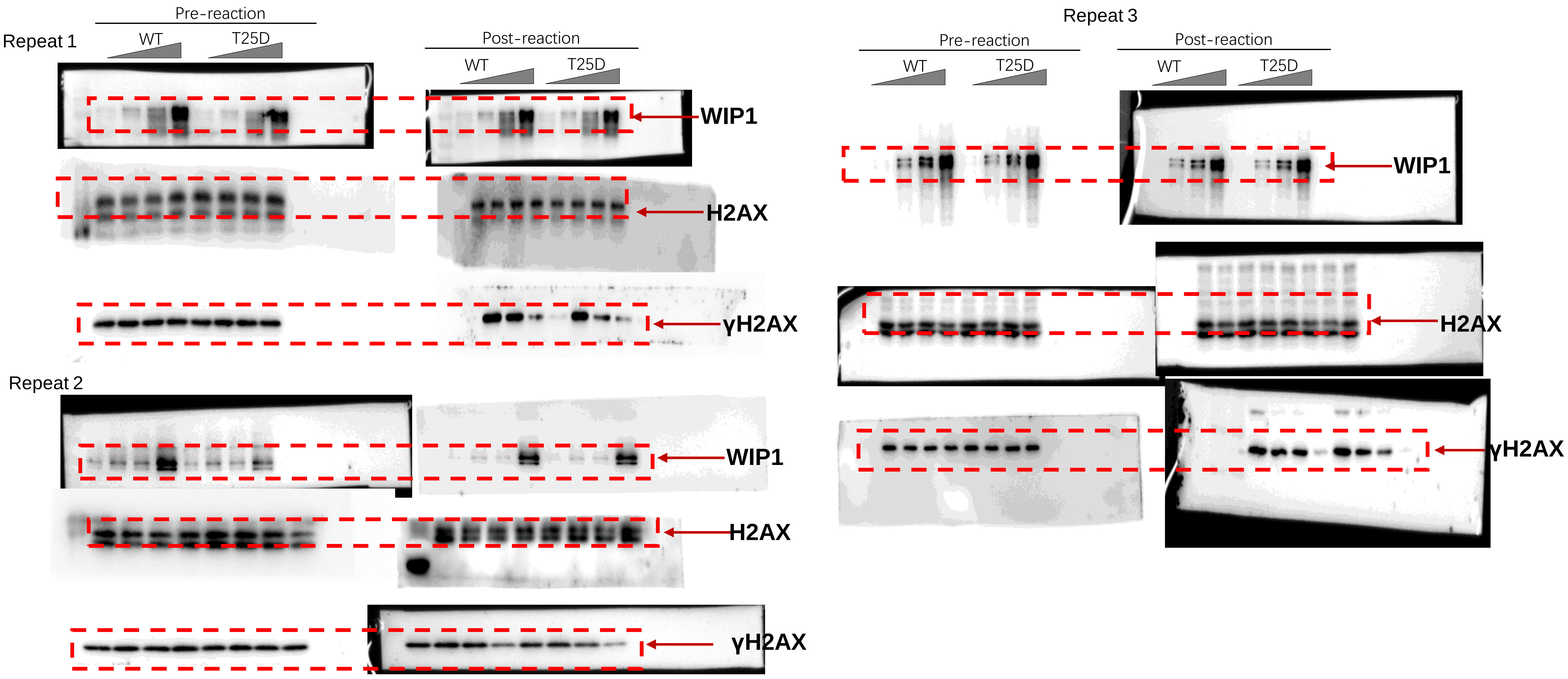
Repeat 1
Pre-reaction
WT
T25D
Post-reaction
WT
T25D
WIP1
H2AX
γH2AX
Repeat 2
WIP1
H2AX
γH2AX
Repeat 3
Pre-reaction
WT
T25D
Post-reaction
WT
T25D
WIP1
H2AX
γH2AX

Sup 6B

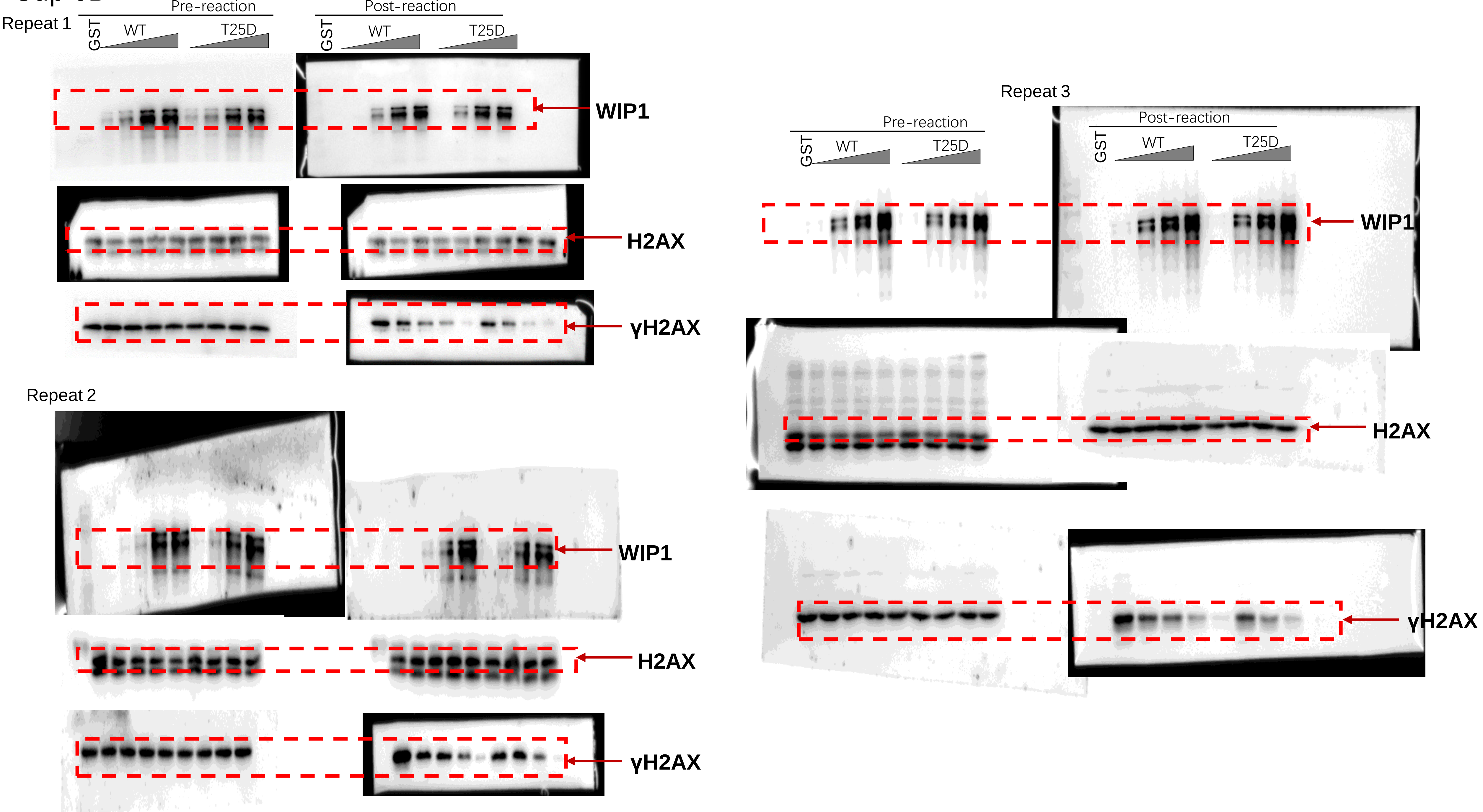
Repeat 1
Pre-reaction
Post-reaction
GST
WT
T25D
WIP1
H2AX
γH2AX
Repeat 2
WIP1
H2AX
γH2AX
Repeat 3
Pre-reaction
Post-reaction
GST
WT
T25D
WIP1
H2AX
γH2AX

Sup 8A

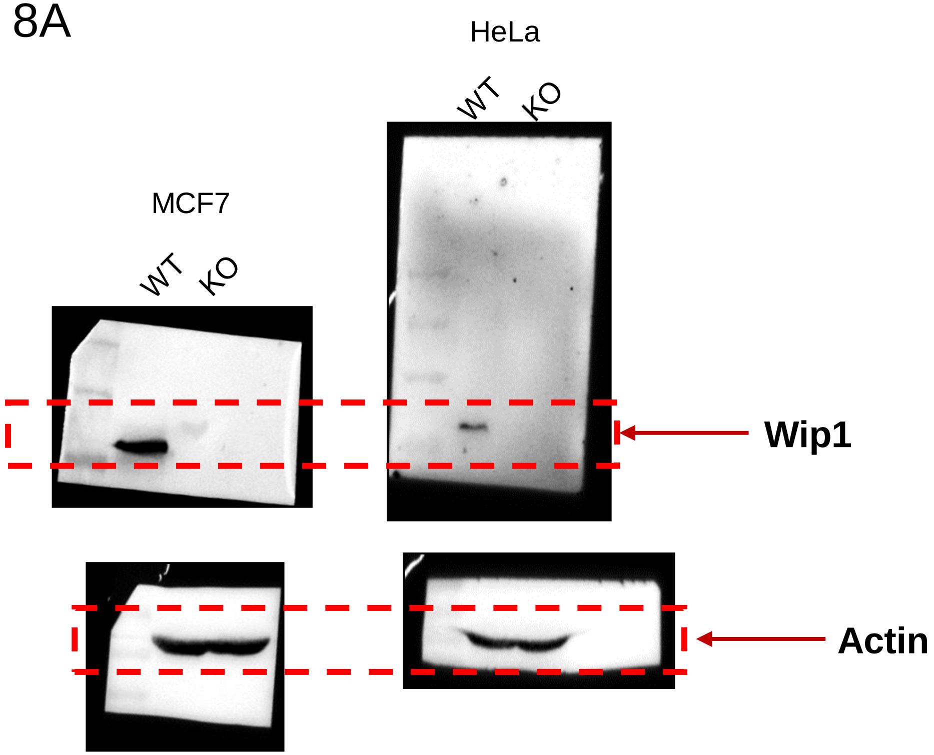

Supplement: Supplementary file 14 — original blots [file 41419_2025_8141_MOESM14_ESM.pdf]
